# Supplementary material for: Analysis of change in patient-reported outcome measures with floor and ceiling effects using the multilevel Tobit model: a simulation study and an example from a National Joint Register using body mass index and the Oxford Hip Score
Source: BMJ Open. 2020 Aug 27;10(8):e033646. doi: 10.1136/bmjopen-2019-033646 (PMC7454239; doi:10.1136/bmjopen-2019-033646)

## Supplementary Figures

*Supplementary.Figure 1: Inclusion / Exclusion Criteria of the NJR study.*

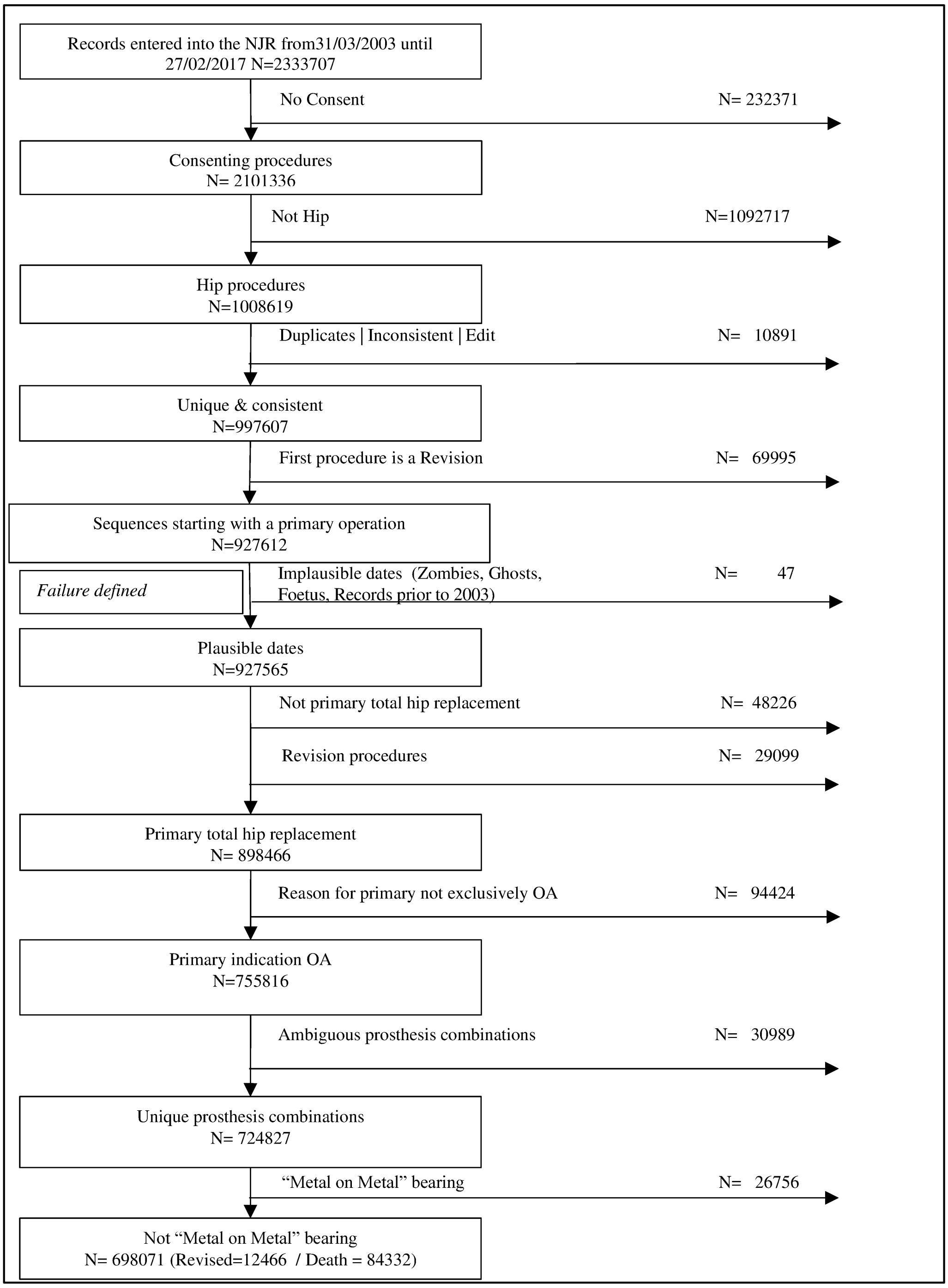

*Supplementary.Figure 2: Description of covariate missing data in eligible data*

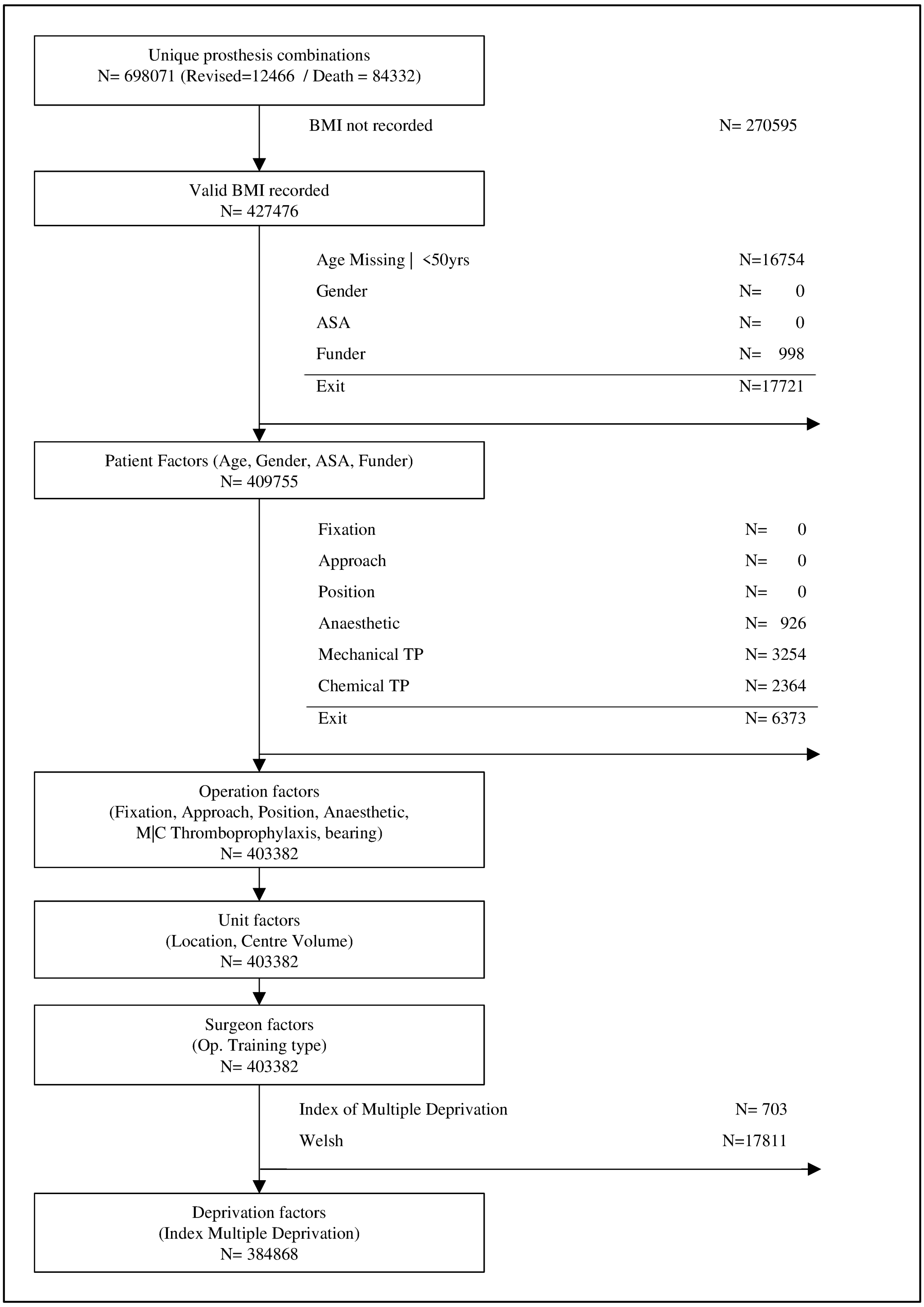

*Supplementary.Figure 3: Description of National PROMS linkage to the NJR*

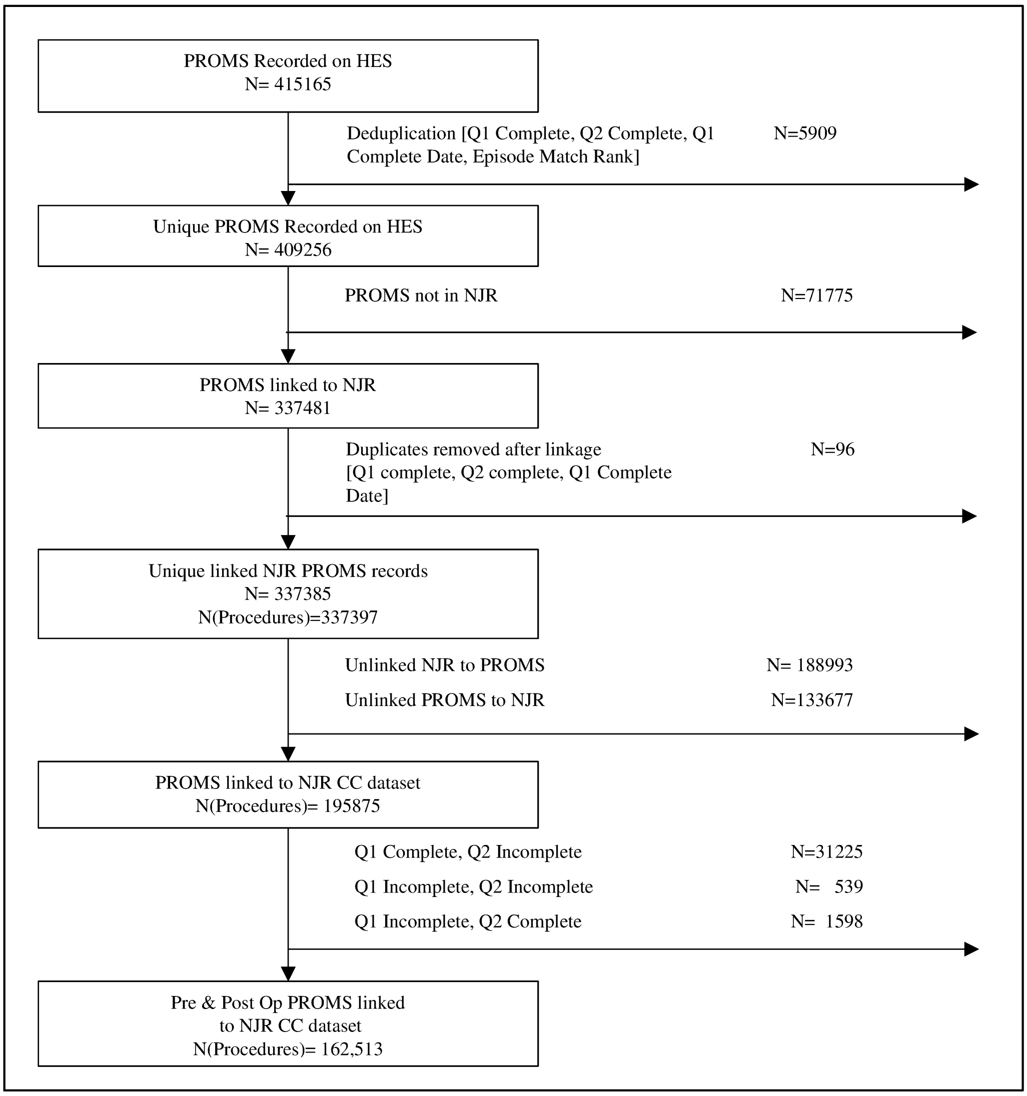

*Supplementary.Figure 4: "Zip Plot" of the 1000 95% Confidence intervals for each method of analysis for DGP 1. The vertical axis is the centile of the two-sided p-value against $H_0$ : $\beta_3$=0 associated with the confidence interval for MLM and ML Tobit models.*

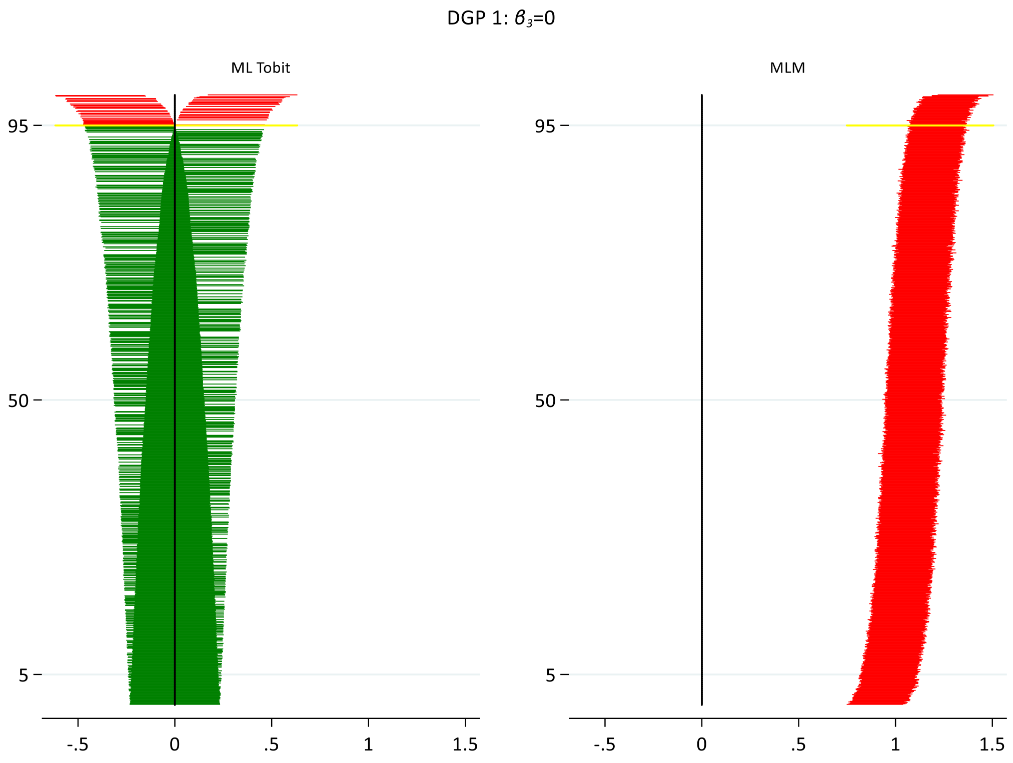

*Supplementary.Figure 5: "Zip Plot" of the 1000 95% Confidence intervals for each method of analysis for DGP 1. The vertical axis is the centile of the two-sided p-value against $H_0$ : $\beta_3$=0 associated with the confidence interval for ML Tobit models with varying constraints of $\sigma_\varepsilon^2$*

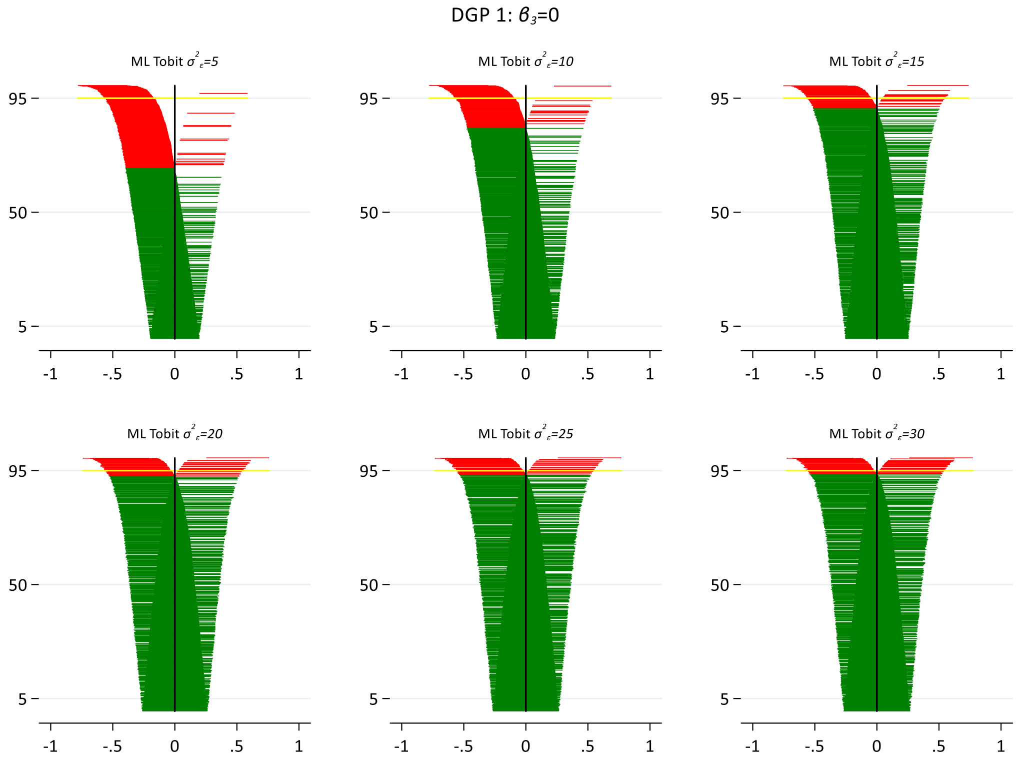

*Supplementary.Figure 6: "Zip Plot" of the 1000 95% Confidence intervals for each method of analysis for DGP 1. The vertical axis is the centile of the two-sided p-value against $H_0$ : $\beta_3=0$ associated with the confidence interval for Single level OLS and Tobit models.*

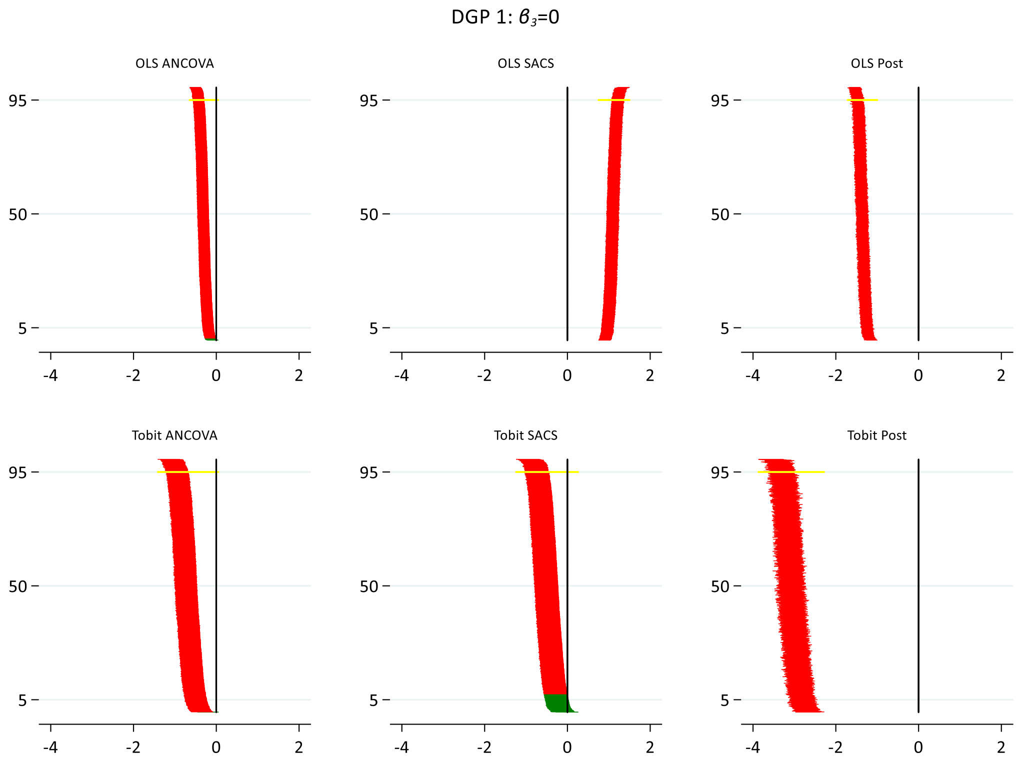

*Supplementary.Figure 7: "Zip Plot" of the 1000 95% Confidence intervals for each method of analysis for DGP 2. The vertical axis is the centile of the two-sided p-value against $H_0$ : $\beta_3$=3 associated with the confidence interval for MLM and ML Tobit models.*

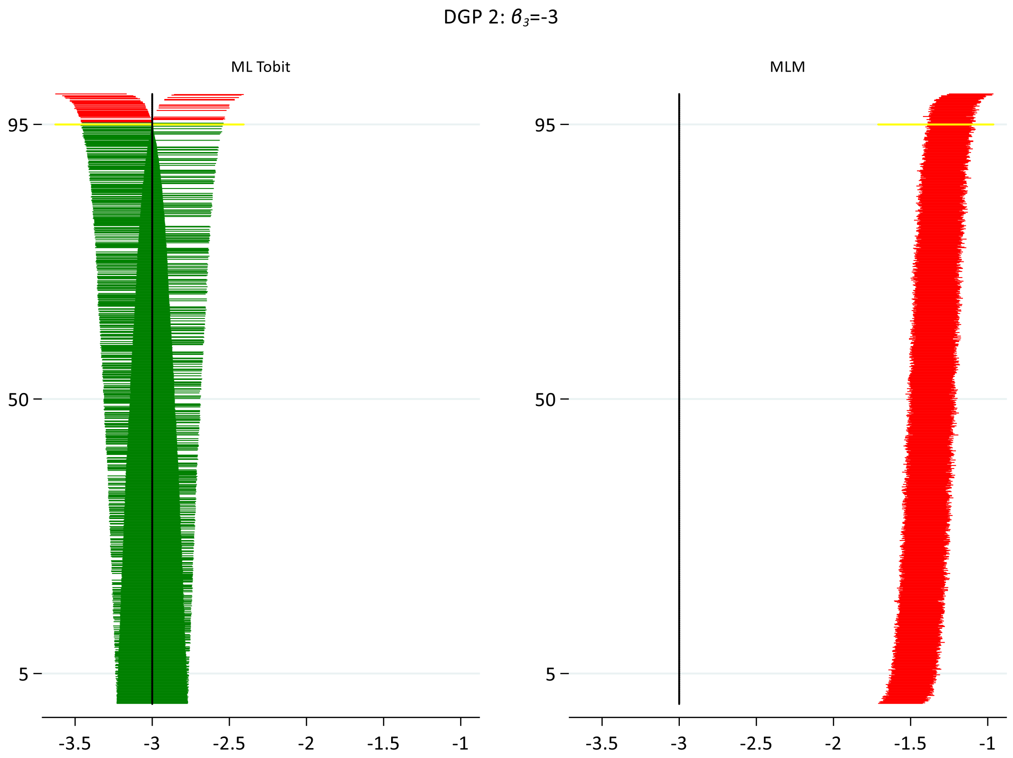

*Supplementary.Figure 8: "Zip Plot" of the 1000 95% Confidence intervals for each method of analysis for DGP 2. The vertical axis is the centile of the two-sided p-value against $H_0$ : $\beta_3$=3 associated with the confidence interval for ML Tobit models with varying constraints of* $\sigma_\varepsilon^2$

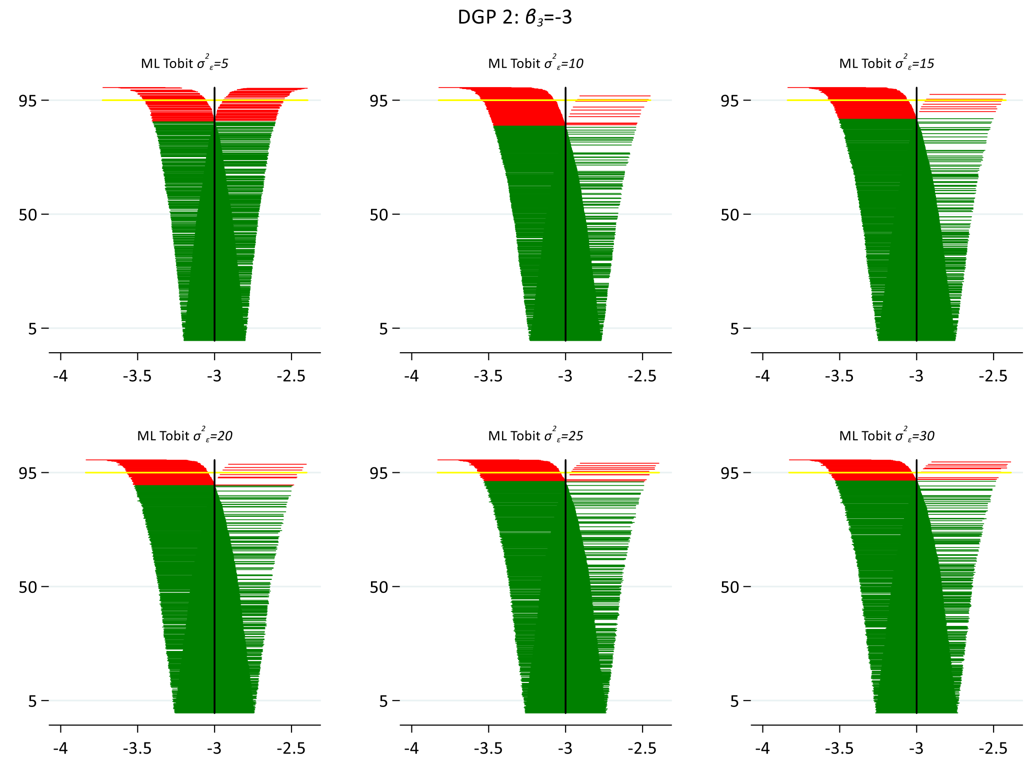

*Supplementary.Figure 9: "Zip Plot" of the 1000 95% Confidence intervals for each method of analysis for DGP 2. The vertical axis is the centile of the two-sided p-value against $H_0$ : $\beta_3$=3 associated with the confidence interval for Single level OLS and Tobit models.*

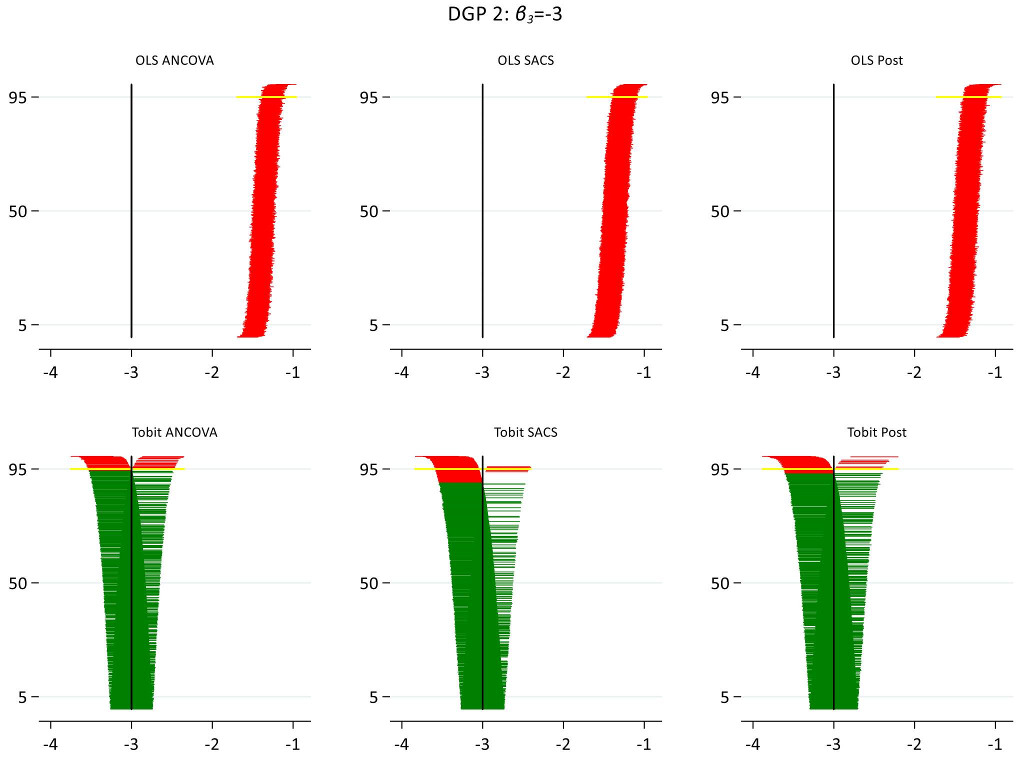

*Supplementary.Figure 10: "Zip Plot" of the 1000 95% Confidence intervals for each method of analysis for DGP 3. The vertical axis is the centile of the two-sided p-value against $H_0$ : $\beta_3$=3 associated with the confidence interval for MLM and ML Tobit models.*

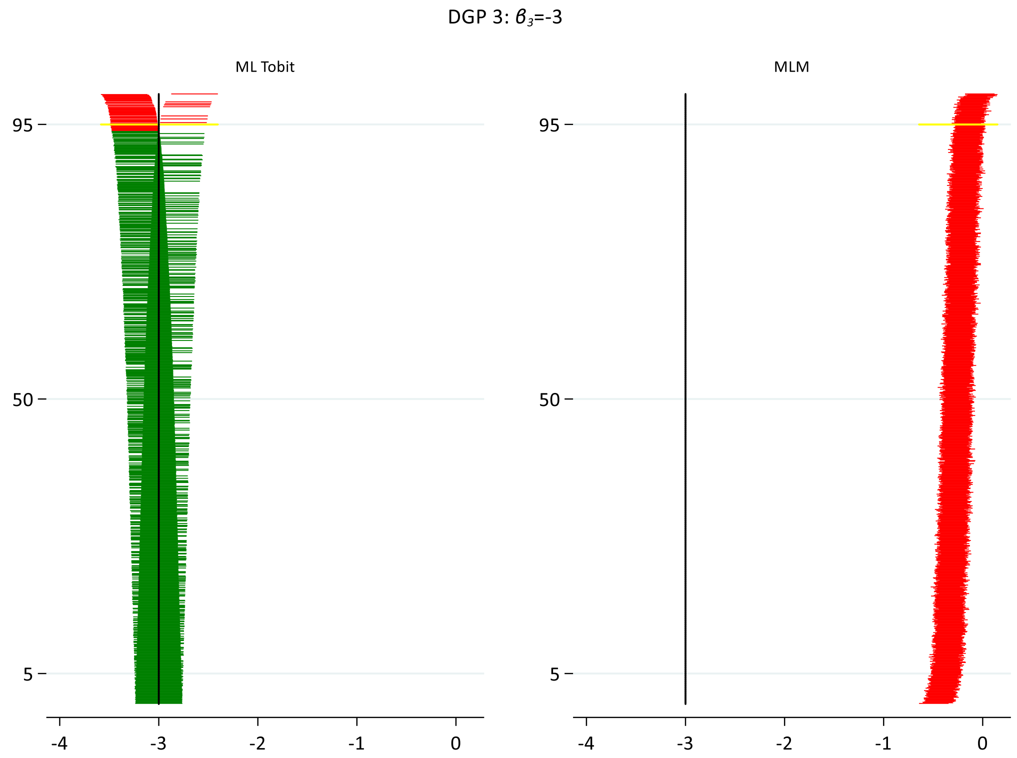

*Supplementary.Figure 11: "Zip Plot" of the 1000 95% Confidence intervals for each method of analysis for DGP 3. The vertical axis is the centile of the two-sided p-value against $H_0$ : $\beta_3$=3 associated with the confidence interval for ML Tobit models with varying constraints of $\sigma_\varepsilon^2$*

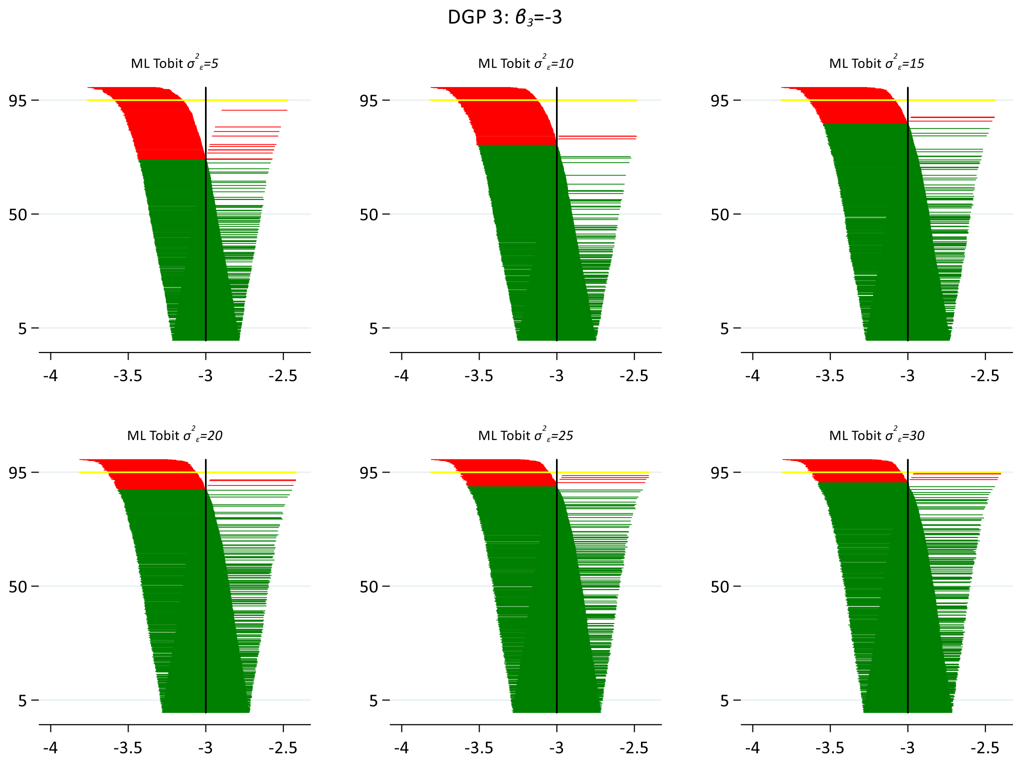

*Supplementary.Figure 12: "Zip Plot" of the 1000 95% Confidence intervals for each method of analysis for DGP 3. The vertical axis is the centile of the two-sided p-value against $H_0$ : $\beta_3$=3 associated with the confidence interval for Single level OLS and Tobit models.*

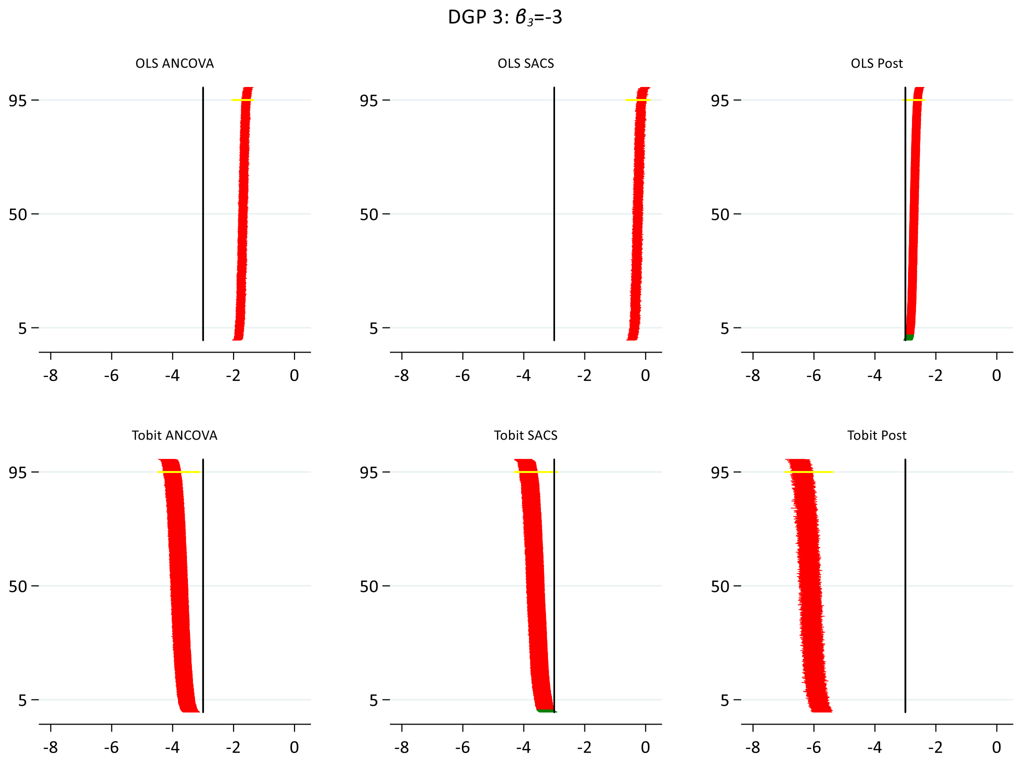

*Supplementary.Figure 13: "Zip Plot" of the 1000 95% Confidence intervals for each method of analysis for DGP 4. The vertical axis is the centile of the two-sided p-value against $H_0$ : $\beta_3$=0 associated with the confidence interval for MLM and ML Tobit models.*

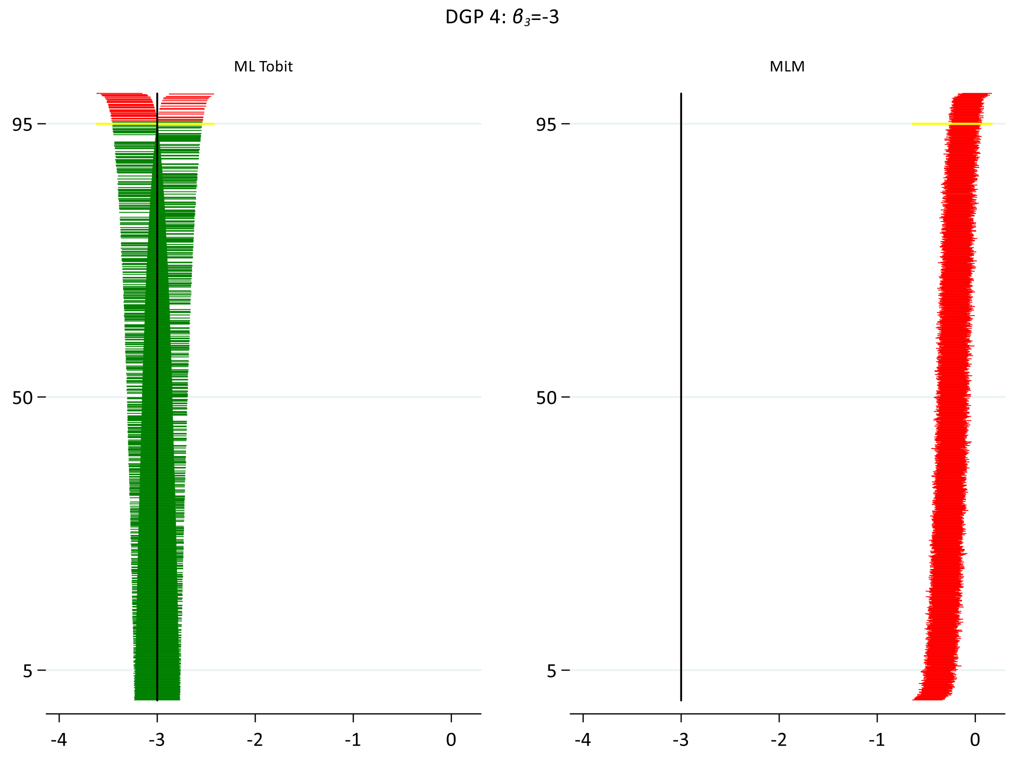

*Supplementary.Figure 14: "Zip Plot" of the 1000 95% Confidence intervals for each method of analysis for DGP 4. The vertical axis is the centile of the two-sided p-value against $H_0$ : $\beta_3$=0 associated with the confidence interval for ML Tobit models with varying constraints of* $\sigma_\varepsilon^2$

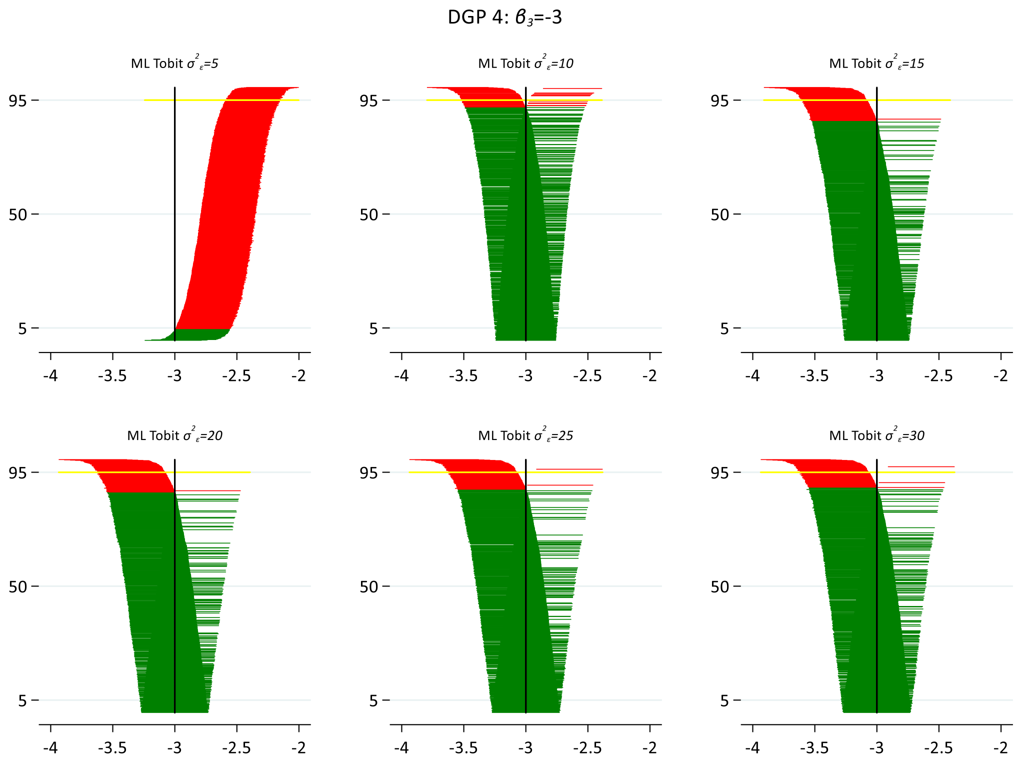

*Supplementary.Figure 15: "Zip Plot" of the 1000 95% Confidence intervals for each method of analysis for DGP 4. The vertical axis is the centile of the two-sided p-value against $H_0$ : $\beta_3$=0 associated with the confidence interval for Single-level OLS and Tobit models.*

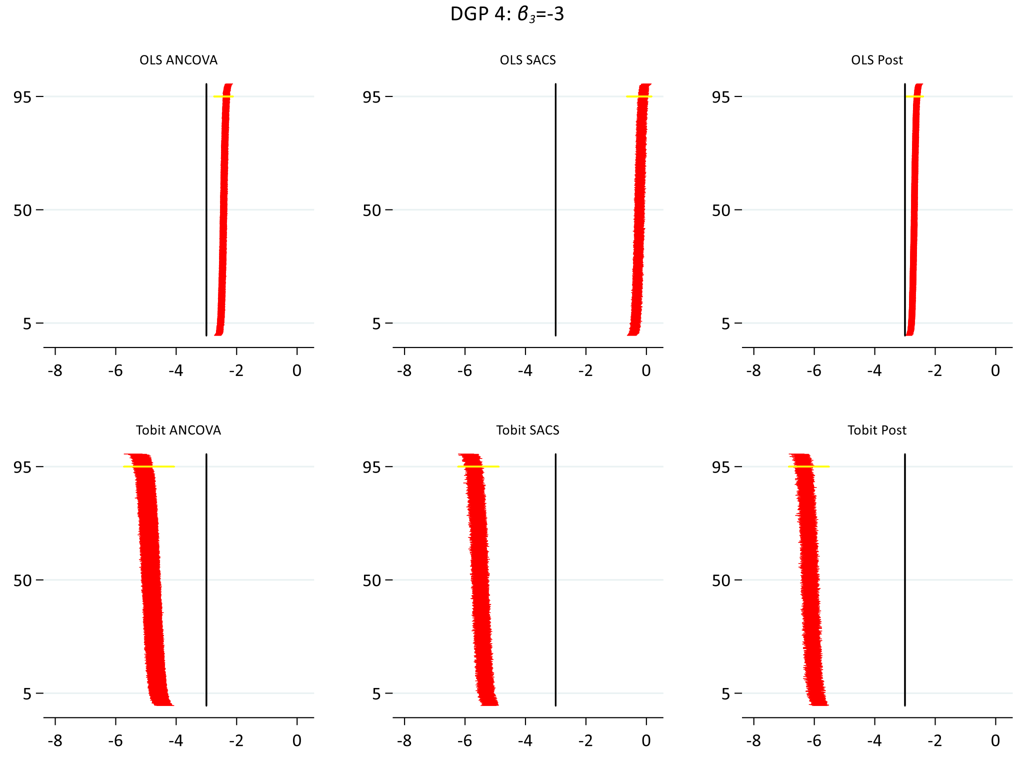

*Supplementary.Figure 16: Marginal effect of level 1 error variance ($\sigma_\varepsilon^2$) constraint on level 2 variance components*

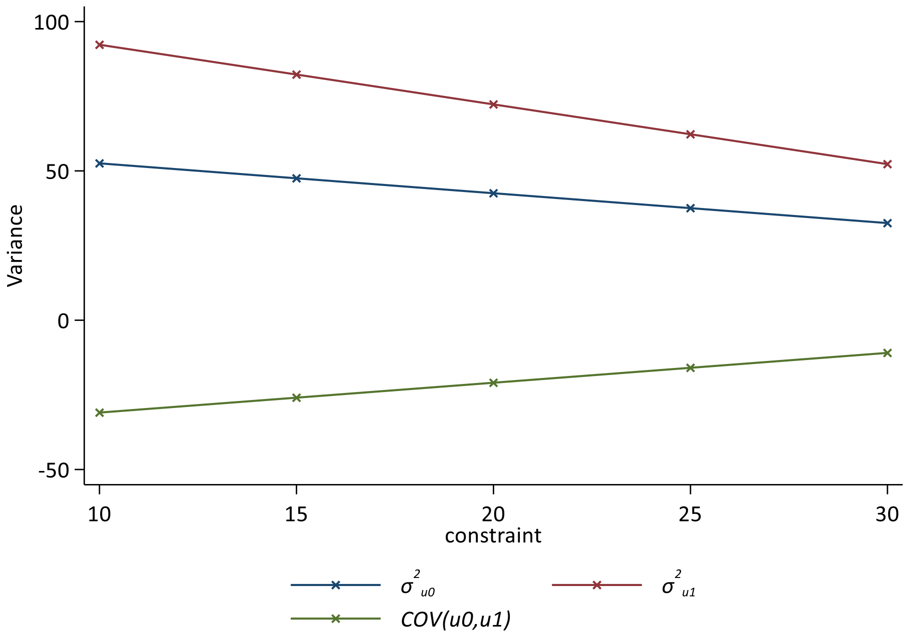

Supplement: Supplementary data [file bmjopen-2019-033646supp002.pdf]
