# Supplementary material for: Analysis of change in patient-reported outcome measures with floor and ceiling effects using the multilevel Tobit model: a simulation study and an example from a National Joint Register using body mass index and the Oxford Hip Score
Source: BMJ Open. 2020 Aug 27;10(8):e033646. doi: 10.1136/bmjopen-2019-033646 (PMC7454239; doi:10.1136/bmjopen-2019-033646)
Supplement: Supplementary data [file bmjopen-2019-033646supp001.pdf]

# Supplementary Tables

*Supplementary.Table 1: Simulation estimates of performance characteristics (Monte Carlo Standard Error in parantheses)*

| | Model | DGP 1: $\beta_3$ =0 | | DGP2 : $\beta_3$ =-3 | | DGP 3: $\beta_3$ =-3 | | DGP 4 : $\beta_3$ =-3 | |
|---|---|---|---|---|---|---|---|---|---|
| Emperical SE | MLM | 0.075 | (0.0017) | 0.072 | (0.0016) | 0.075 | (0.0017) | 0.079 | (0.0018) |
| | ML Tobit | 0.12 | (0.0027) | 0.12 | (0.0026) | 0.12 | (0.0026) | 0.12 | (0.0026) |
| | ML Tobit $\sigma_\varepsilon^2$=5 | 0.14 | (0.0031) | 0.13 | (0.0029) | 0.14 | (0.0032) | 0.12 | (0.0027) |
| | ML Tobit $\sigma_\varepsilon^2$ =10 | 0.14 | (0.0031) | 0.13 | (0.003) | 0.14 | (0.0032) | 0.13 | (0.0029) |
| | ML Tobit $\sigma_\varepsilon^2$=15 | 0.14 | (0.0031) | 0.13 | (0.003) | 0.14 | (0.0032) | 0.14 | (0.003) |
| | ML Tobit $\sigma_\varepsilon^2$=20 | 0.14 | (0.0031) | 0.13 | (0.003) | 0.14 | (0.0032) | 0.14 | (0.0031) |
| | ML Tobit $\sigma_\varepsilon^2$=25 | 0.14 | (0.0031) | 0.13 | (0.003) | 0.14 | (0.0032) | 0.14 | (0.0031) |
| | ML Tobit $\sigma_\varepsilon^2$=30 | 0.14 | (0.0031) | 0.13 | (0.003) | 0.14 | (0.0032) | 0.14 | (0.0031) |
| | OLS SACS | 0.075 | (0.0017) | 0.072 | (0.0016) | 0.075 | (0.0017) | 0.079 | (0.0018) |
| | OLS ANCOVA | 0.07 | (0.0016) | 0.064 | (0.0014) | 0.068 | (0.0015) | 0.059 | (0.0013) |
| | OLS Post | 0.072 | (0.0016) | 0.07 | (0.0016) | 0.071 | (0.0016) | 0.056 | (0.0013) |
| | Tobit SACS | 0.14 | (0.0031) | 0.13 | (0.0029) | 0.14 | (0.0031) | 0.15 | (0.0034) |
| | Tobit ANCOVA | 0.14 | (0.0032) | 0.13 | (0.003) | 0.14 | (0.0032) | 0.13 | (0.003) |
| | Tobit Post | 0.16 | (0.0035) | 0.15 | (0.0034) | 0.16 | (0.0035) | 0.13 | (0.0028) |
| Mean Square Error | MLM | 1.21 | (0.0052) | 2.69 | (0.0075) | 7.54 | (0.013) | 7.66 | (0.014) |
| | ML Tobit | 0.014 | (0.0007) | 0.014 | (0.0006) | 0.015 | (0.0007) | 0.014 | (0.0006) |
| | ML Tobit $\sigma_\varepsilon^2$=5 | 0.036 | (0.0015) | 0.017 | (0.0008) | 0.038 | (0.0015) | 0.2 | (0.0033) |
| | ML Tobit $\sigma_\varepsilon^2$=10 | 0.028 | (0.0012) | 0.025 | (0.0011) | 0.041 | (0.0015) | 0.019 | (0.0009) |
| | ML Tobit $\sigma_\varepsilon^2$=15 | 0.023 | (0.001) | 0.026 | (0.0011) | 0.035 | (0.0013) | 0.032 | (0.0014) |
| | ML Tobit $\sigma_\varepsilon^2$=20 | 0.021 | (0.0009) | 0.024 | (0.001) | 0.032 | (0.0013) | 0.033 | (0.0014) |
| | ML Tobit $\sigma_\varepsilon^2$=25 | 0.02 | (0.0009) | 0.023 | (0.001) | 0.03 | (0.0012) | 0.032 | (0.0014) |
| | ML Tobit $\sigma_\varepsilon^2$=30 | 0.02 | (0.0009) | 0.022 | (0.001) | 0.029 | (0.0012) | 0.031 | (0.0014) |
| | OLS SACS | 1.21 | (0.0052) | 2.69 | (0.0075) | 7.54 | (0.013) | 7.66 | (0.014) |
| | OLS ANCOVA | 0.1 | (0.0014) | 2.68 | (0.0066) | 1.71 | (0.0056) | 0.33 | (0.0021) |
| | OLS Post | 1.86 | (0.0062) | 2.68 | (0.0073) | 0.085 | (0.0013) | 0.097 | (0.0011) |
| | Tobit SACS | 0.54 | (0.0064) | 0.018 | (0.0008) | 0.63 | (0.0069) | 3.37 | (0.018) |
| | Tobit ANCOVA | 0.27 | (0.0046) | 0.027 | (0.0011) | 0.39 | (0.0056) | 6.25 | (0.021) |
| | Tobit Post | 9.42 | (0.03) | 0.027 | (0.0012) | 9.81 | (0.031) | 9.88 | (0.025) |
| Relative Error | MLM | -1.31 | (2.21) | 1.98 | (2.28) | 2.74 | (2.3) | -0.31 | (2.23) |
| | ML Tobit | -0.95 | (2.22) | 0.79 | (2.25) | 2.37 | (2.29) | 1.66 | (2.27) |
| | ML Tobit $\sigma_\varepsilon^2$=5 | -28.5 | (1.6) | -22.8 | (1.73) | -23.8 | (1.71) | -7.01 | (2.08) |
| | ML Tobit $\sigma_\varepsilon^2$=10 | -14.5 | (1.91) | -10.3 | (2.01) | -8.8 | (2.04) | -3.97 | (2.15) |
| | ML Tobit $\sigma_\varepsilon^2$=15 | -7.35 | (2.07) | -3.54 | (2.16) | -2.41 | (2.18) | -2.46 | (2.18) |
| | ML Tobit $\sigma_\varepsilon^2$=20 | -4.29 | (2.14) | -0.57 | (2.22) | 0.26 | (2.24) | -1.4 | (2.21) |
| | ML Tobit $\sigma_\varepsilon^2$=25 | -3.04 | (2.17) | 0.65 | (2.25) | 1.35 | (2.27) | -0.8 | (2.22) |
| | ML Tobit $\sigma_\varepsilon^2$=30 | -2.51 | (2.18) | 1.17 | (2.26) | 1.83 | (2.28) | -0.47 | (2.23) |
| | OLS SACS | -1.3 | (2.21) | 1.99 | (2.28) | 2.75 | (2.3) | -0.3 | (2.23) |
| | OLS ANCOVA | -0.5 | (2.23) | 1.51 | (2.27) | 6.84 | (2.39) | -0.54 | (2.23) |
| | OLS Post | -1.96 | (2.19) | -0.086 | (2.24) | 1.46 | (2.27) | -1.25 | (2.21) |

| | | | | | | | | | |
|---|---|---|---|---|---|---|---|---|---|
| | Tobit SACS | -2.97 | (2.17) | 2.09 | (2.28) | 2.82 | (2.3) | -0.056 | (2.24) |
| | Tobit ANCOVA | -0.19 | (2.23) | 2.21 | (2.29) | 4.01 | (2.33) | -1.35 | (2.21) |
| | Tobit Post | -2.82 | (2.17) | 1.27 | (2.27) | 2.74 | (2.3) | 0.83 | (2.26) |
| Relative Precision | MLM | 247 | (14.1) | 232.3 | (13.9) | 275.4 | (16.9) | 132.5 | (6.66) |
| | ML Tobit | 37.8 | (4.36) | 28.3 | (4.07) | 50.5 | (5.64) | 6.08 | (3.13) |
| | ML Tobit $\sigma_{\varepsilon}^{2}$=5 | | | | | | | | |
| | ML Tobit $\sigma_{\varepsilon}^{2}$=10 | -0.034 | (0.97) | -0.92 | (1.07) | 3.91 | (1.24) | -13.3 | (1.5) |
| | ML Tobit $\sigma_{\varepsilon}^{2}$=15 | 0.62 | (1.22) | -1.66 | (1.37) | 3.24 | (1.52) | -22.5 | (1.68) |
| | ML Tobit $\sigma_{\varepsilon}^{2}$=20 | 1.22 | (1.3) | -1.7 | (1.44) | 2.57 | (1.6) | -26 | (1.7) |
| | ML Tobit $\sigma_{\varepsilon}^{2}$=25 | 1.67 | (1.33) | -1.52 | (1.47) | 2.31 | (1.62) | -27.2 | (1.7) |
| | ML Tobit $\sigma_{\varepsilon}^{2}$=30 | 1.99 | (1.34) | -1.34 | (1.47) | 2.29 | (1.63) | -27.5 | (1.69) |
| | OLS SACS | 247 | (14.1) | 232.3 | (13.9) | 275.4 | (16.9) | 132.5 | (6.66) |
| | OLS ANCOVA | 297.2 | (16) | 327.4 | (14.8) | 357.6 | (20.9) | 310.3 | (18.8) |
| | OLS Post | 281.7 | (15.5) | 250 | (14.8) | 312.9 | (19.2) | 360.1 | (21.2) |
| | Tobit SACS | 2.26 | (1.94) | 1.8 | (1.89) | 7.48 | (2.72) | -38.6 | (2.12) |
| | Tobit ANCOVA | -5.67 | (3.07) | -2.33 | (1.55) | 1.52 | (3.56) | -19.1 | (3.59) |
| | Tobit Post | -19.5 | (2.6) | -22.5 | (2.6) | -15.9 | (2.95) | -10.6 | (4.08) |
