# Supplementary material for: Analysis of change in patient-reported outcome measures with floor and ceiling effects using the multilevel Tobit model: a simulation study and an example from a National Joint Register using body mass index and the Oxford Hip Score
Source: BMJ Open. 2020 Aug 27;10(8):e033646. doi: 10.1136/bmjopen-2019-033646 (PMC7454239; doi:10.1136/bmjopen-2019-033646)
Supplement: Author's manuscript [file bmjopen-2019-033646.draft_revisions.pdf]

BMJ Open is committed to open peer review. As part of this commitment we make the peer review history of every article we publish publicly available.

When an article is published we post the peer reviewers' comments and the authors' responses online. We also post the versions of the paper that were used during peer review. These are the versions that the peer review comments apply to.

The versions of the paper that follow are the versions that were submitted during the peer review process. They are not the versions of record or the final published versions. They should not be cited or distributed as the published version of this manuscript.

BMJ Open is an open access journal and the full, final, typeset and author-corrected version of record of the manuscript is available on our site with no access controls, subscription charges or pay-per-view fees (http://bmjopen.bmj.com).

If you have any questions on BMJ Open's open peer review process please email info.bmjopen@bmj.com

BMJ Open

Analysis of change in patient reported outcome measures with floor and ceiling effects using the multi-level Tobit model: A simulation study and an example from a National Joint Register using body mass index and the Oxford Hip Score.

Journal:	*BMJ Open*
Manuscript ID	bmjopen-2019-033646
Article Type:	Original research
Date Submitted by the Author:	19-Aug-2019
Complete List of Authors:	Sayers, Adrian; University of Bristol, Muscloskeletal Research Unit<br>Whitehouse, Michael; University of Bristol, Musculoskeletal Research Unit<br>Judge, Andrew; University of Bristol, Musculoskeletal Research Unit, Translational Health Sciences, Bristol Medical School, University of Bristol, Learning and Research Building, Level 1, Southmead Hospital, Southmead, BS10 5NB<br>MacGregor, Alex; University of East Anglia, Norwich Medical School<br>Blom, AW; University of Bristol,<br>Ben-Shlomo, Yoav; University of Bristol,
Keywords:	Multi-level Tobit Model, Change Scores, Epidemiologic Methods, Arthroplasty, Patient Reported Outcome Measures, Longitudinal Studies

SCHOLARONE™
Manuscripts

I, the Submitting Author has the right to grant and does grant on behalf of all authors of the Work (as defined in the below author licence), an exclusive licence and/or a non-exclusive licence for contributions from authors who are: i) UK Crown employees; ii) where BMJ has agreed a CC-BY licence shall apply, and/or iii) in accordance with the terms applicable for US Federal Government officers or employees acting as part of their official duties; on a worldwide, perpetual, irrevocable, royalty-free basis to BMJ Publishing Group Ltd ("BMJ") its licensees and where the relevant Journal is co-owned by BMJ to the co-owners of the Journal, to publish the Work in this journal and any other BMJ products and to exploit all rights, as set out in our licence.

The Submitting Author accepts and understands that any supply made under these terms is made by BMJ to the Submitting Author unless you are acting as an employee on behalf of your employer or a postgraduate student of an affiliated institution which is paying any applicable article publishing charge ("APC") for Open Access articles. Where the Submitting Author wishes to make the Work available on an Open Access basis (and intends to pay the relevant APC), the terms of reuse of such Open Access shall be governed by a Creative Commons licence – details of these licences and which Creative Commons licence will apply to this Work are set out in our licence referred to above.

Other than as permitted in any relevant BMJ Author's Self Archiving Policies, I confirm this Work has not been accepted for publication elsewhere, is not being considered for publication elsewhere and does not duplicate material already published. I confirm all authors consent to publication of this Work and authorise the granting of this licence.

For peer review only

Analysis of change in patient reported outcome measures with floor and ceiling effects using the multi-level Tobit model: A simulation study and an example from a National Joint Register using body mass index and the Oxford Hip Score.

Adrian Sayers[1,2], Michael R Whitehouse[1,3], Andrew Judge[1,3], Alexander Macgregor[4], Ashley W Blom[1,3], Yoav Ben-Shlomo[2].

Author affiliations:

1. Musculoskeletal Research Unit, Bristol Medical School, 1st Floor Learning & Research Building, Southmead Hospital, Bristol, BS10 5NB, United Kingdom
2. Population Health Sciences, Bristol Medical School, Canynge Hall, 39 Whatley Road Bristol, BS8 2PS, United Kingdom
3. National Institute for Health Research Bristol Biomedical Research Centre, University of Bristol
4. Norwich Medical School, University of East Anglia, Norwich, United Kingdom

Corresponding Author

Adrian Sayers

Musculoskeletal Research Unit, Bristol Medical School, 1st Floor Learning & Research Building, Southmead Hospital, Bristol, BS10 5NB, United Kingdom

Email: adrian.sayers@bristol.ac.uk Tel: 0117 4147880 Conflict of Interest: The authors have no conflict of interests

For peer review only

Abstract (290 Words)

Objectives

Analysis of pre/post intervention change in observational studies using Patient Reported Outcome Measures (PROMs) is often believed to be a trivial exercise, and guidance for analysis of data from randomised controls trials is often applied. This is often inappropriate, and that analysis of change scores may be preferable. However, it is unclear if this is suitable in outcomes with floor and ceiling effects. We investigate the association between body mass index (BMI) and the efficacy of primary hip replacement.

Design

Simulation study and prospective national medical device register

Setting

National register of joint replacement and medical devices

Methods

Using a Monte-Carlo simulation study and data from a national joint replacement register (162,513 patients with pre/post surgery PROMs) we investigate simple approaches for the analysis of outcomes with floor and ceiling effects that are measured at two occasions: linear and Tobit regression (baseline adjusted ANCOVA, change-score analysis, post-score analysis) in addition to linear and multi-level Tobit models.

Primary outcome

The primary outcome of interest is change in patient reported outcome measures from pre-surgery to 6 months post-surgery.

Results

Analysis of data with floor and ceiling effects with models that fail to account for these features induce substantial bias. Single level Tobit models only correct for floor or ceiling effects when the exposure of interest is not associated with the baseline score. In observational data scenarios, only multi-level Tobit models are capable of providing unbiased inferences.

Conclusions

Inferences from pre/post studies that fail to account for floor and ceiling effects may induce spurious associations with substantial risk of bias. Multi-level Tobit models indicate the efficacy of total hip replacement is independent of BMI. Restricting access to total hip replacement based on a patients BMI can not be supported by the data.

For peer review only

Strengths and limitations of this study

- We use a simulation study and large prospective study set to investigate the effect of floor and ceiling effects in the analysis of change in patient reported outcome measure pre- and post-surgery.
- We demonstrate that mutli-level Tobit models generate unbiased estimates of change in patient reported outcome measures with floor and ceiling effects.
- Simple change score and baseline adjusted ANCOVA generate estimates that are biased in non-randomised experiments.
- We demonstrate the efficiacy of total hip replacement is independent of a patients BMI, and restriction to joint replacement based on a patients BMI can not be supported by the data.

Keywords

Multi-level Tobit Model, Change Scores, Epidemiologic Methods, Arthroplasty, Patient Reported Outcome Measures, Longitudinal Studies

For peer review only

Introduction

In many non-randomised experiments, researchers are interested in assessing how change in health status is associated with a covariate of interest. Whilst there is much guidance available on assessing change in randomised experiments, and extensive discussion with respect to efficiency and bias [1-9], the guidance in non-randomised studies is less clear. The principle difference is that in observational studies we do not expect balance between different levels of an exposure at baseline, in addition to expecting imbalance in other confounding factors. Glymour et al. advocate the use of, simple analysis of, change scores (SACS) without baseline adjustment to achieve unbiased causal effect estimates using causal arguments presented through Directed Acyclic Graphs (DAGS) [10]. They briefly suggest that in settings with floor and/or ceiling effects, that standard change analyses with and without baseline adjustment are both biased, and non-standard analyses based on Tobit models (censored regression) may ameliorate floor and ceiling problems. The degree to which Tobit models ameliorate the problems caused by floor and ceiling effects is unclear. Some authors suggest that using percentage change is one strategy to avoid dealing with floor and ceiling effects, but Twisk highlighted that this simply represents a linear transformation of change [11], and therefore does not deal with the problem of floor and ceiling effects. Twisk also describes the use of a longitudinal (multi-level) Tobit regression model to appropriately account for floor and ceiling effects in studies with repeated measures [12]. However, since its publication in 2009 there have only be a handful of analyses that use multi-level Tobit models[13-16], suggesting that lack of familiarity with these methods or understanding of when they can and should be applied has deterred analysts in their use, or when they can be applied.

Multi-level Tobit models are now incorporated in mainstream statistical software packages, such as Stata. Given their accesibility, they could arguably be used more frequently than they are. This is relavent considering that the use of measurement instruments with floor and ceiling effects are omnipresent in health related research. Examples include outcomes in health related quality of life (e.g. EQ5D, SF-36, SF-12), psychological wellbeing (e.g Hospital Anxiety and Depression Scale (HADS), Edinburgh Postnatal Depression Scale (EPDS)), and disease specific measures of wellbeing (e.g. Western Ontario and McMaster Universities Osteoarthritis Index (WOMAC) and Oxford Hip Score (OHS) as used in patients with osteoarthritis). Despite this, there is very little guidance available with respect to the consequences of using measurement instruments with floor or ceiling effects, when attempting to make inferences about the effect of an exposure on the change (between two time points) of an outcome of interest.

In this paper we use a Monte-Carlo simulation study to compare the performance of multi-level linear and Tobit models, Ordinarily Least Squares (OLS) regression and single-level Tobit regression, with and without adjustment for baseline scores, in the analysis of change in three different non-

For peer review only

randomised experiments, and a randomised experiment. We also demonstrate the use of these models using real world data from a large national joint replacement register.

We motivate the simulation and exemplar data analysis using an example from joint replacement research describing the association between body mass index (BMI) and the change in a disease specific patient reported outcome measure (PROM), the Oxford Hip Score (OHS). The issue is contentious in the UK [17-19] and USA [20] as some organisations suggest restricting joint-replacement to patients based on their BMI, citing an increased risk of revision surgery and lack of efficacy of surgery. The small increase in absolute risk of revision in obese patients, must be balanced against the other benefits of joint replacement, including a reduction in pain and improved physical functioning. Therefore, it is of interest to clinicians, policy makers, and patients to know the relative effect of obesity on the efficacy of total hip replacement compared to "normal weight patients".

For peer review only

Methods

Simulation Study Aims

We investigated the performance of four different methods of analysis, when estimating the effect of an exposure (BMI) on change in response (PROM) before and after total hip replacement with floor and ceiling effects using the Aims, Data Generating Process (DGP), Methods, Estimand, Performance (ADMEP) approach recomended by Morris et al. [21].

Data Generating Process (DGP)

We simulated longitudinal data of "well-being" before and after surgery. We assume that "well-being" is a latent, truly continuous and stable construct which is measured imperfectly by the OHS. Measurement error and floor/ceiling effects are then added to the latent construct to illustrate their consequences.

We assume the response, well-being, is a latent construct (y^*_{ij}) measured at the ith occasion, where i varies from 0 (pre-surgery) to 1 (1 year post-surgery), for the jth individual is modelled as a linear function of time. x_{0j} is mean-centred BMI categories according to WHO criteria i.e. -2 = BMI<18.5 (under weight), -1=18.5<BMI≤25 (normal), 0=25<BMI≤30 (overweight), 1=30<BMI≤35 (obese), and 2= BMI>35 (morbidly obese), i.e. x_{0j}=0 is a patient with a BMI classed as overweight.

$$y^*_{ij} = \beta_0 + u_{0j} + (\beta_1 + u_{1j})t_{ij} + \beta_2 x_{0j} + \beta_3 x_{0j}\, t_{ij}$$
$$\begin{bmatrix} u_{0j} \\ u_{1j} \end{bmatrix} \sim N(0, \Omega_u),\ \Omega_u = \begin{bmatrix} \sigma^2_{u0} & \\ \sigma_{u01} & \sigma^2_{u1} \end{bmatrix} \qquad 1$$

where t_{ij} is the time at which measurement i was taken on individual j, coded as 0 at pre-surgery and 1 post-surgery. $\beta_0$is the baseline population average response for a patient with average BMI, and u_{0j} represents the jth individual difference from the baseline response. The sum of $\beta_0 + u_{0j}$ is the individual baseline response for a patient with average BMI. β_1 represents the population average change per unit increase in time for a patient with average BMI, and u_{1j} represents the jth individual difference from the population average change per unit increase in time. The sum $\beta_1 + u_{1j}$ is the individual average change per unit increase in time for a patient with average BMI. β_2 represents the effect of a 1-unit increase in the exposure (x_{0j}) of interest (BMI) pre-surgery and β_3 represents the effect of a 1-unit increase in BMI (x_{0j}) on the pre-post surgery change in well-being (y^*_{ij}). The variance in individual deviations from the population average response at baseline and the average rate of change are σ^2_{u0} and σ^2_{u1} respectively. The covariance between baseline measurements and rate of change is characterised by σ_{u01} (with correlation ρ_{u01}).

For peer review only

Under the assumption of linear change, data was simulated from a multi-level model with a random intercept and slope, see Figure 1 for an illustration of a patient trajectory with an average BMI.

<Figure 1 Here>

The observed response without floor and ceiling effects (y_{ij}) is simulated by adding measurement error in the linear trajectory, ε_{ij} , to the latent response, where $\varepsilon_{ij} \sim N(0, \sigma_{\varepsilon}^2)$.

$$y_{ij} = y_{ij}^* + \varepsilon_{ij} \qquad 2$$

A response with floor and ceiling effects y_{ij}^{FC} is simulated by restricting the response to lie between 0 and 48.

$$y_{ij}^{FC} = \begin{cases} 0 & \text{if } y_{ij} \leq 0 \\ y_{ij} & \text{if } 0 < y_{ij} < 48 \\ 48 & \text{if } y_{ij} \geq 48 \end{cases} \qquad 3$$

See Figure 2 for a graphical illustration of the trajectory generation: we first simulate y_{ij}^*, then add some measurement error (ε_{ij}) to yield an observed response (y_{ij}), and finally add floor and ceiling effects to obtain the observed truncated response (y_{ij}^{FC}).

<Figure 2 Here>

We compared 4 DGPs to illustrate a range of scenarios by manipulating β_2, β_3, and $\rho_{u01}(\sigma_{u01})$ to influence the association between pre- and post-surgery outcomes. β_0, β_1, σ_{u0}, σ_{u1}, and $\sigma_{\varepsilon ij}$were fixed at 10, 40, 10, 15 and 3 respectively. **DGP 1** is a null model, where there is a baseline effect of the exposure is β_2= -3, but the exposure did not influence change over time (β_3= 0), and there is no correlation between baseline values and subsequent change (ρ_{u01}=0).

DGP 2 replicates a simple randomised trial where there is no difference between levels of the exposure at baseline (β_2= 0), but the exposure did influence change over time (β_3= -3), and there is no correlation between baseline values and subsequent change (ρ_{u01}=0). **DGP 3** and **DGP 4** replicate a cohort study, where there is a difference between levels of the exposure at baseline (β_2=-3), and the exposure also influenced change over time (β_3= -3). **DGP 3** specified no correlation between baseline values and subsequent change (ρ_{u01}=0), whereas **DGP 4** specified a negative correlation between baseline values and change (ρ_{u01}=-0.5), reflecting the fact the joint-replacement surgery has the tendency to normalise an individuals well-being, see Figure 3 for an illustration of the associated trajectories.

<Figure 3 Here>

We conducted a Monte-Carlo simulation with 1000 replicated datasets, each with 10,000 patients. A balanced dataset, i.e. 3 data points for each individual, was simulated to ensure identification of the

For peer review only

linear and Tobit multi-level models occurred, i.e. two data points allows estimation of baseline and change parameters but not measurement error. The middle data point was then dropped to replicate a pre/post design.

Method of analysis

For data sets with 3 measurement occasions, a linear multi-level model and a multi-level Tobit model (MLTM) that reflects the data generating process were fitted to the data, see equation 1.

In datasets with 2 measurement occasions, i.e. a pre-post design, single-level OLS and Tobit models were fitted to the data. Tobit models were only used when floor and ceiling effects had been simulated. Three different models were explored:

1) A simple model for post surgery well-being.

$$y_{1j}^{FC} = \alpha_1 + \alpha_2 x_{0j} + \varepsilon_j \quad (4)$$

2) A Simple Analysis of Change Score (SACS).

$$\left(y_{1j}^{FC} - y_{0j}^{FC}\right) = \alpha_6 + \alpha_7 x_{0j} + \varepsilon_j \quad (5)$$

3) A model for change adjusted for baseline i.e. baseline adjusted ANCOVA. This model is equivalent to a model for the post score adjusted for baseline ANCOVA, with the exception of the interpretation of the intercept.

$$\left(y_{1j}^{FC} - y_{0j}^{FC}\right) = \alpha_8 + \alpha_9 x_{0j} + \alpha_{10} y_{0j}^{FC} + \varepsilon_j \quad (6)$$

In addition, an under-identified MLTM model, equivalent to equation 1with constrained error variance σ_ε^2 was fitted in the spirit of a sensitivity analysis, where σ_ε^2 was constrained to a value from 5, 10, 15, 20, 25 and 30.

Estimand

The estimand of interest is the population average effect of the interaction between the exposure and change in slope i.e. β_3 the pre-post surgery change in well-being. We test whether the exposure modifies the improvement post-surgery (i.e. the null hypothesis that $\beta_3 = 0$).

Performance

The performance of each method was explored in terms of bias, coverage, empirical standard error, model based standard error, mean square error, relative error and relative precision.

National Joint Registry of England, Wales, Northern Ireland, and the Isle of Man (NJR)

For peer review only

Using data from the NJR, we investigated the association between BMI and a patient reported outcome measure, the OHS, in patients undergoing elective total hip replacement (THR) between 1st April 2003 and 22nd February 2017.

Patient and public involvement

Patient representatives sit on the committee structure of the National Joint Registry. The research priorities of the National Joint Registry are identified by this committee structure and approved by the patient representatives. Patients were not involved in the setting of the research question or the outcome measures, nor were they involved in designing or implementing this work or interpretation of the results. We are unable to disseminate results of this study directly to study participants due to the anonymous nature of the data. We plan to disseminate our findings to the National Joint Registry, via their communications team, to relevant to the provision of joint replacement and to the general population through the local and national press.

Data source

The NJR commenced data collection in April 2003; at inception it was mandatory for all THRs conducted in the private sector to be entered into the NJR, and from 2011 all THR procedures in the public and private sector were required to be entered into the NJR. A recent national audit of data entered into the NJR between 2014 and 2015 estimated data capture of 95% for primary THR and 91% for revision THR.

Inclusion/exclusion criteria

All consenting patients undergoing THR were eligible to be included in the analysis. Patients were included if their patient history was unique and consistent, i.e. contained no duplicates, revision prior to primary, or currently held in query by the submitting unit. Due to the requirement for reliable date information, patients who were indicated to have died prior to undergoing a procedure, were more than 110 years of age, had undergone a procedure prior to their date of birth, or received a procedure prior to 2003 were excluded from the analysis. Only primary THRs, where the primary indication for operation was osteoarthritis (OA) with unique prosthesis combinations were included in the analysis. All THRs with metal-on-metal bearing combinations were excluded from the analysis due to the exceptionally high failure rate in this group[22, 23]. Patients who were less than 50 years of age at the date of the index THR were also excluded, due to the high likelihood that these cases are due to OA secondary to other pathology.

See Supplementary.Figure 1 and Supplementary.Figure 2 for a detailed breakdown of inclusion criteria and missing data.

For peer review only

Primary outcome

The primary outcome of interest in this study is change in OHS after surgery. Linked National PROMs were first available in 2009, see Supplementary.Figure 3 for details of linkage.

Primary exposure

The primary exposure of interest in this study is BMI. BMI was introduced into the second "Minimal Data Set" in 2004. Patients with BMI between 10 and 60 were included in the analysis. BMI measures were excluded as implausible if height and weight measures were less than 130cm and weight less than 30kg respectively. See Supplementary.Figure 2.

Confounding factors

Pre-operative confounding factors were thematically organised into groups: 1) Patient factors included sex, American Society of Anesthesiologists (ASA) grade, and operation funder. 2) Operation factors included fixation, approach, patient position during surgery, anaesthetic type, thromboprophylaxis regime, bearing, and year of primary THR. 3) The setting of the treatment episode (i.e. private or NHS hospital). 4) Consultant based factors included the training status of the primary surgeon performing the operation. 5) Deprivation factors were based on the English indices of multiple deprivation (an area based index of deprivation).

Statistical analyses

Means, standard deviations and interquartile points were used to describe continuous variables. Frequencies and percentages were used to describe categorical variables.

The association between change in PROMS score was investigated using the same single-level methods and the ML Tobit model with constrained error variances described in the simulation study as an exemplar. In addition, we conducted more comprehensive analyses using restricted cubic splines to model the BMI association in the ML Tobit model with constrained error variance, single-level linear and Tobit SACS, ANCOVA, and Post score models. In the ML Tobit model, BMI was modelled with restricted cubic splines at baseline and its interaction with time. Correspondingly, we adjusted OHS for patient and deprivation confounding factors at baseline and operation, setting and confounding factors with an interaction with time i.e. operative factors and settings influence the change in outcome but not the baseline response. In single-level models, the effect of BMI was modelled using restricted cubic splines and adjusted for confounding factors using standard regression approaches.

Missing data

Due to the method of data collection in the national PROMS program, item non-response is masked. Defacto mean imputation of up to two missing items in the OHS occurred automatically. In addition,

For peer review only

despite valid values appearing with individual OHS items, if the questionnaire was marked as "not complete", implausible overall scores were obtained. For simplicity only patients with complete pre-operative and post-operative PROMS were used in the analysis. BMI is missing in a substantial proportion of the cohort. Patients prior to 2004 did not have BMI recorded, and the proportion of patients with missing BMI in 2004 is large. In 2009 ~40% of patients did not have BMI recorded; this reduced year on year and in 2016 was ~18% of eligible patients.

For pedagogic simplicity we use complete-case analyses throughout.

Results

Simulation Study

Figure 4 illustrate the results from the MC simulation for each DGP. It is clear that MLM, OLS methods, and in DGP's 1, 3, and 4 (observational scenarios) single-level Tobit models all exhibit substantial bias. Only the ML Tobit with 3 datapoints provides unbiased estimates in all scenarios. Constrained ML Tobit models are close to being unbiased, but slightly over estimate the effect size, see Table 1, Single-level Tobit models also provide unbiased estimates for DGP 2 (the randomised trial).

Figure 5 illustrates the spread of model based standard errors (SE) for each method by DGP. It is clear that the variation and absolute magnitude of SE in MLM with 3 data points per person is less than that of ML Tobit Models. Similarly, model based SEs from OLS methods are smaller and less variable than single-level Tobit methods. In DGP 2, the randomised trial, it is interesting to note that the SE from Tobit ANCOVA models are marginally smaller than for Tobit SACS. Whilst there is little difference in terms of bias from the constrained ML Tobit models, see Figure 4, the size and variability of estimated SEs increased with increasing value of the constrained of σ^2_ε.

Supplementary.Figure 4, 5, 6, 7, 8, 9, 10, 11, 12, 13, 14, and 15, illustrate the coverage of 95% confidence intervals in each DGP. Unsurprisingly, coverage of methods which demonstrate bias is very poor, whilst coverage is at nominal levels for the ML Tobit model with three data points. The results from constrained ML Tobit indicate coverage less than the nominal levels. Coverage less than the advertised levels is principally due to the bias in estimate. However, when the estimates from the model are unbiased, as in DGP 2 with σ^2_ε=5, coverage is poor, suggesting bias in model based SE, i.e. they are too small.

National Joint Registry of England, Wales, Northern Ireland, and the Isle of Man.

Following application of inclusion and exclusion criteria, there were 162,513 patients with pre and post-operative OHS available for analysis. Figure 6 illustrates the results of the exemplar dataset using

For peer review only

different approaches whilst attempting to estimate the effect of BMI category on the efficacy of surgery, whereas Figure 7 and Figure 8 illustrate the use of restricted cubic splines to assess the same question.

Exemplar Analysis

A single-level OLS SACS appoach suggests a positive association between BMI and change in OHS i.e. patients with greater BMIs have greater gains in well-being, whereas OLS ANCOVA and OLS post score models suggest a negative association. The single-level Post model score is approximately 50% greater than the ANCOVA model. All single-level Tobit models suggest a negative association between BMI and OHS. The Tobit SACS model is the smallest, with both the Tobit ANCOVA and Post models estimating substantially larger effects. The constrained ML Tobit models all provide equivalent (to 2 decimal places) results, suggesting there is no effect of BMI on the change in OHS pre and post surgery, see Figure 6.

Restricted Cubic Spline Approach

Crude analyses, which model BMI using restricted cubic splines, illustrate a complex association between BMI and pre-operative OHS. A ~4.5 point reduction in OHS is observed as BMI increases between 20 and 50 $kg.m^{-2}$. However, the change in OHS between pre- and post- surgery is very weakly associated with pre-operative BMI, with individuals with BMI's <25 $kg.m^{-2}$ and >45 $kg.m^{-2}$ receiving modestly greater gains than those patients with an average BMI of 28 $kg.m^{-2}$. However, with less than ½ a unit variation across the range of BMI observed in the cohort, the difference falls well below anything that could be considered clinically meaningful, see Figure 7. Following adjustment for patient factors, operation factors, centre factors, consultant factors, and deprivation there was little difference in the pattern of change compared to crude results, see Figure 7. Single-level approaches are illustrated in Figure 8, with OLS and Tobit models giving similar patterns of results. ANCOVA and the post model specification suggest a strong inverse association with BMI, with obese individuals receiving less improvement following surgery. OLS SACS indicate that obesity is associated with greater gains in OHS following surgery. Conversely, Tobit SACS models indicate that obesity is associated with smaller gains in OHS following surgery.

Discussion

The results of the simulation study clearly illustrate that, in the presence of floor and ceiling effects, neither baseline adjustment, or simple analysis of change scores (SACS) will yield unbiased estimates of the effect of an exposure on the outcome of interest. Single-level modifications to account for floor and ceiling effects such as the Tobit model only work in the context of a randomised trial, i.e. when there is no difference between baseline values by BMI. Importantly, single-level methods, OLS and

For peer review only

Tobit models, induce significant bias, with negligible coverage, when $\beta_3 = 0$ i.e. there is no change in the pre- post- surgergy well being by BMI. Fully identified MLTM with three measurement occasions, return unbiased estimates with coverage close to advertised levels. In pre- post- designs with two measurement occasions ML Tobit models, with constrained level 1 variances, return estimates very close to being unbiased, but coverage is less than advertised indicating bias in the model based standard errors.

The simulation study is consistent with a lay intuition with respect to analyses of floor and ceiling effects. Assuming we accept that either the MLM and OLS change analyses are appropriate in the absence of floor and ceiling effects, DGP 1 illustrates that when there is no effect of obesity on the efficacy of surgery, the addition of an artificial ceiling compresses the gain of individuals towards the top of the distribution. Due to the baseline association between obesity and well-being, underweight individuals tend to have gains that are more compressed compared to obese individuals. This inevitably induces bias, and provides evidence of a change in pre- post- surgery wellbeing by BMI, where none actually exist. Similarly, in DGP 2 (no baseline differences) where there is truly an interaction effect, will also lead to biased estimates. The DGP used in the simulation assumes underweight individuals benefit more from surgery than heavier individuals, which results in a fanning out of the trajectories. Underweight individuals have truly greater gains than obese individuals, but these gains are underestimated due to the ceiling effect, resulting in bias towards the null. In DGPs 3 and 4 (baseline differences in BMI, and interaction between BMI and change) we see a more extreme pattern of results compared to DGP 2, but overall consistency with the expected response of compressing individual gains which have initially higher starting values.

In the exemplar analysis of NJR data, the pattern of results is very similar to that of DGP 1 of the simulation, suggesting that results of the simulation are likely to be replicated in real world datasets. The more comprehensive analysis of the NJR data, using RCS to reflect the continuous nature of BMI, aptly illustrate where the effects from mis-specified single-level models are arising from. The ML Tobit model illustrates a strong negative association between BMI and pre-operative OHS, and failing to account for these baseline differences appropriately when attempting to estimate change leads to variation at baseline being incorporated in the estimate of change. Furthermore, the ability to adjust both baseline and post-surgical OHS for their pronounced floor and ceiling effects respectively, leads to unbiased estimates of the effect of interest. Unfortunately, due to the constraints on the level 1 variance, interpretation of the random effects are difficult, as they depend on the magnitude of the variance applied in the constraint, see Supplementary.Figure 16. However, the models clearly illustrate that change in PROMS following THR do not depend on BMI, and surgery appears to be effective for patients regardless of their BMI.

Conclusion

For peer review only

Floors and ceilings in PROM instruments have somewhat predictable effects on estimated coefficients from standard OLS models that do not adjust for floor or ceiling effects, assuming the true underlying association is known. As this is rarely the case, it is important to consider a variety of different data generating processes to explore the likely impact on an analysis. It is important to consider the validity of the assumptions underpinning the Tobit model, i.e. that the latent response is truly continuous and that there is a true ceiling just beyond the range of the measurement being used.

Single-level Tobit models do not ameliorate floor and ceiling effects in simple analysis of change scores. However, ML Tobit models appear to recover the effects of interest under specific assumptions. The analysis of pre- post- designs require further constraints to ensure models are fully identified. The difference between analytical approaches can profoundly alter the interepretation of the model parameters, and this may have serious consequences if used to generate policy inappropriately. For example, inappropriate analyses that fail to consider data generating process appropriately may lead to the restriction of joint replacement for overweight or obese patients.

When designing a study to investigate the effect of an exposure on change in health status, it would be preferable to use a measurement instrument that does not have floor or ceiling effects as inference is less complicated, and design trumps analysis in most scenarios. If the use of measurement instrument with floor and ceiling effects is unavoidable, it is preferable to collect data at 3 time points which ensure models are fully identified, alleviating the need to constrain level 1 variance in order to identify models, again design trumps analysis. If retrospective analysis of pre-post data sets are required, it appears that using ML Tobit model with constrained level 1 error variance would be preferable to single-level approaches.

Broadly speaking the analyses of this simulation are in agreement with the work of Glymour et al., that analysis of change and its interaction with an exposure at baseline, should not be adjusted for baseline measurements in observational data. The presence of floor and ceiling effects in data requires additional assumptions which makes things marginally more complex.

For peer review only

Author Contributions

AS, MRW, AJ, AM, AWB, and YBS were responsible for the study design, AS conducted the data analysis. AS, MRW, AJ, AM, AWB, and YBS were responsible for interpreting the data. AS, MRW, AJ, AM, AWB, and YBS prepared and edited and approved the final manuscript.

Competing interests statement

We declare no competing interests

Funding

AS is funded by an MRC Strategic Skills Fellowship MR/L01226X/1

This study was supported by the NIHR Biomedical Research Centre at University Hospitals Bristol NHS Foundation Trust and the University of Bristol. The views expressed in this publication are those of the author(s) and not necessarily those of the NHS, the National Institute for Health Research or the Department of Health and Social Care.

Data Sharing Statement

Access to the data can be made via research requests to the National Joint Registry of England, Wales, Northern Ireland and the Isle of Man. Full details can be found at http://www.njrcentre.org.uk/njrcentre/Research/Research-requests

For peer review only

Tables

Table 1:

Model	DGP 1: β_3 =0		DGP2 : β_3 =-3		DGP 3: β_3=-3		DGP 4 : β_3 =-3	
Estimate								
MLM	1.1	(0.0024)	-1.36	(0.0023)	-0.26	(0.0024)	-0.23	(0.0025)
ML Tobit	-0.0056	(0.0038)	-3.03	(0.0037)	-3.04	(0.0037)	-3.01	(0.0037)
ML Tobit σ_ϵ^2=5	-0.13	(0.0044)	-3.01	(0.0042)	-3.13	(0.0046)	-2.57	(0.0038)
ML Tobit σ_ϵ^2=10	-0.093	(0.0044)	-3.09	(0.0042)	-3.14	(0.0045)	-3.05	(0.0041)
ML Tobit σ_ϵ^2=15	-0.057	(0.0044)	-3.09	(0.0042)	-3.12	(0.0045)	-3.11	(0.0043)
ML Tobit σ_ϵ^2=20	-0.04	(0.0044)	-3.08	(0.0042)	-3.11	(0.0045)	-3.12	(0.0044)
ML Tobit σ_ϵ^2=25	-0.031	(0.0044)	-3.07	(0.0042)	-3.1	(0.0045)	-3.11	(0.0044)
ML Tobit σ_ϵ^2=30	-0.026	(0.0044)	-3.07	(0.0042)	-3.09	(0.0045)	-3.1	(0.0045)
OLS SACS	1.1	(0.0024)	-1.36	(0.0023)	-0.26	(0.0024)	-0.23	(0.0025)
OLS ANCOVA	-0.31	(0.0022)	-1.36	(0.002)	-1.69	(0.0021)	-2.43	(0.0019)
OLS Post	-1.36	(0.0023)	-1.36	(0.0022)	-2.72	(0.0023)	-2.69	(0.0018)
Tobit SACS	-0.72	(0.0044)	-3.04	(0.0041)	-3.78	(0.0044)	-4.83	(0.0048)
Tobit ANCOVA	-0.5	(0.0046)	-3.09	(0.0042)	-3.61	(0.0045)	-5.5	(0.0042)
Tobit Post	-3.07	(0.0049)	-3.06	(0.0047)	-6.13	(0.005)	-6.14	(0.004)
Coverage								
MLM	0	(0)	0	(0)	0	(0)	0	(0)
ML Tobit	94.8	(0.7)	95.3	(0.67)	93.9	(0.76)	95.4	(0.66)
ML Tobit σ_ϵ^2=5	67.5	(1.48)	86.7	(1.07)	71.6	(1.43)	4.6	(0.66)
ML Tobit σ_ϵ^2=10	83.5	(1.17)	85	(1.13)	77.4	(1.32)	92.2	(0.85)
ML Tobit σ_ϵ^2=15	91.1	(0.9)	87.7	(1.04)	85.7	(1.11)	86.8	(1.07)
ML Tobit σ_ϵ^2=20	92.7	(0.82)	90	(0.95)	88.2	(1.02)	87.1	(1.06)
ML Tobit σ_ϵ^2=25	93.4	(0.79)	91.6	(0.88)	89.5	(0.97)	88.2	(1.02)
ML Tobit σ_ϵ^2=30	93.6	(0.77)	91.9	(0.86)	91.1	(0.9)	88.9	(0.99)
OLS SACS	0	(0)	0	(0)	0	(0)	0	(0)
OLS ANCOVA	0.8	(0.28)	0	(0)	0	(0)	0	(0)
OLS Post	0	(0)	0	(0)	2.4	(0.48)	0	(0)
Tobit SACS	0.1	(0.1)	94.4	(0.73)	0	(0)	0	(0)
Tobit ANCOVA	7.2	(0.82)	89.7	(0.96)	1.2	(0.34)	0	(0)
Tobit Post	0	(0)	93.3	(0.79)	0	(0)	0	(0)
Model SE								
MLM	0.074	(2E-05)	0.074	(2E-05)	0.077	(2E-05)	0.078	(2E-05)
ML Tobit	0.12	(3E-05)	0.12	(3E-05)	0.12	(3E-05)	0.12	(3E-05)
ML Tobit σ_ϵ^2=5	0.1	(3E-05)	0.1	(3E-05)	0.11	(3E-05)	0.11	(4E-05)
ML Tobit σ_ϵ^2=10	0.12	(3E-05)	0.12	(3E-05)	0.13	(4E-05)	0.12	(3E-05)
ML Tobit σ_ϵ^2=15	0.13	(4E-05)	0.13	(4E-05)	0.14	(4E-05)	0.13	(3E-05)
ML Tobit σ_ϵ^2=20	0.13	(5E-05)	0.13	(5E-05)	0.14	(5E-05)	0.14	(4E-05)
ML Tobit σ_ϵ^2=25	0.14	(5E-05)	0.13	(5E-05)	0.14	(6E-05)	0.14	(5E-05)
ML Tobit σ_ϵ^2=30	0.14	(5E-05)	0.13	(5E-05)	0.15	(6E-05)	0.14	(5E-05)
OLS SACS	0.074	(2E-05)	0.074	(2E-05)	0.077	(2E-05)	0.078	(2E-05)
OLS ANCOVA	0.07	(2E-05)	0.065	(2E-05)	0.072	(2E-05)	0.059	(2E-05)
OLS Post	0.07	(2E-05)	0.07	(2E-05)	0.072	(2E-05)	0.055	(2E-05)
Tobit SACS	0.13	(5E-05)	0.13	(5E-05)	0.14	(6E-05)	0.15	(6E-05)
Tobit ANCOVA	0.14	(6E-05)	0.14	(6E-05)	0.15	(6E-05)	0.13	(6E-05)
Tobit Post	0.15	(7E-05)	0.15	(6E-05)	0.16	(7E-05)	0.13	(6E-05)

For peer review only

For peer review only

Figure Legends

Figure 1: Graphical illustration of a multi-level random intercept and slope model used to generate data for a individual with average BMI.

Figure 2: Graphical illustration of the data generating process of the latent, measured, and measured response with floor and ceiling effects. The Latent Response is y_{ij}^{*}, the measured response is y_{ij}, and the measured response with censoring is y_{ij}^{FC} .

Figure 3: Graphical Illustration of the four Data Generating Processes used to investigate the effect of floor and ceiling effects on analysis of pre-post surgery change with BMI as an exposure. Horizontal red lines at 0 and 48 indicate floor and ceilings of the measurement instrument.

Figure 4: Plot of 1000 estimates by each DGP, for each method of analysis. Within each method, the vertical axis is the repetition number of each simulated dataset. The white pipe symbol is the average of the estimates.

Figure 5: Plot of 1000 estimated Standard Errors by each DGP, for each method of analysis. Within each method, the vertical axis is the repetition number of each simulated dataset. The white pipe symbol is the average of the standard errors.

Figure 6: Estimate and 95% Confidence Intervals of constrained ML Tobit, Single-level OLS and Tobit: ANCOVA, SACS, and Post models.

Figure 7: Estimates and 95% confidence intervals of baseline and change in Oxford Hip Score (OHS) pre and post surgery and its association with Body Mass Index (BMI) adjusted for confounding.

Figure 8: Estimates and 95% confidence intervals of single-level approaches to the analysis of change in Oxford Hip Score (OHS) pre and post surgery and its association with Body Mass Index (BMI) adjusted for confounding.

For peer review only

References

1. Frison L, Pocock SJ. Repeated measures in clinical trials: analysis using mean summary statistics and its implications for design. Stat Med. 1992;11(13):1685-704.
2. Goldstein H. Tutorial in biostatistics-longitudinal data analysis (repeated measures) in clinical trials. Stat Med. 2000;19(13):1821.
3. Kaiser L. Adjusting for baseline: change or percentage change? Stat Med. 1989;8(10):1183-90.
4. Matthews JN, Campbell MJ. Adjusting for baseline: change or percentage change. Stat Med. 1992;11(12):1624-6.
5. Senn S. Repeated measures in clinical trials: analysis using mean summary statistics and its implications for design. Stat Med. 1994;13(2):197-8.
6. Senn S. Change from baseline and analysis of covariance revisited. Stat Med. 2006;25(24):4334-44. doi:10.1002/sim.2682.
7. Senn S, Stevens L, Chaturvedi N. Repeated measures in clinical trials: simple strategies for analysis using summary measures. Stat Med. 2000;19(6):861-77.
8. Vickers AJ. The use of percentage change from baseline as an outcome in a controlled trial is statistically inefficient: a simulation study. BMC Med Res Methodol. 2001;1:6.
9. Vickers AJ, Altman DG. Statistics notes: Analysing controlled trials with baseline and follow up measurements. BMJ. 2001;323(7321):1123-4.
10. Glymour MM, Weuve J, Berkman LF, Kawachi I, Robins JM. When is baseline adjustment useful in analyses of change? An example with education and cognitive change. Am J Epidemiol. 2005;162(3):267-78. doi:10.1093/aje/kwi187.
11. Twisk J. Applied longitudinal data analysis for epidemiology : a practical guide. Cambridge: Cambride University Press; 2004.
12. Twisk J, Rijmen F. Longitudinal tobit regression: a new approach to analyze outcome variables with floor or ceiling effects. J Clin Epidemiol. 2009;62(9):953-8. doi:10.1016/j.jclinepi.2008.10.003.
13. Holla JFM, van Beers-Tas MH, van de Stadt LA, Landewe R, Twisk JWR, Dekker J et al. Depressive mood and low social support are not associated with arthritis development in patients with seropositive arthralgia, although they predict increased musculoskeletal symptoms. RMD Open. 2018;4(1):e000653. doi:10.1136/rmdopen-2018-000653.
14. Moran LJ, Fraser LM, Sundernathan T, Deussen AR, Louise J, Yelland LN et al. The effect of an antenatal lifestyle intervention in overweight and obese women on circulating cardiometabolic and inflammatory biomarkers: secondary analyses from the LIMIT randomised trial. BMC Med. 2017;15(1):32. doi:10.1186/s12916-017-0790-z.
15. Ravona-Springer R, Moshier E, Schmeidler J, Godbold J, Akrivos J, Rapp M et al. Changes in glycemic control are associated with changes in cognition in non-diabetic elderly. J Alzheimers Dis. 2012;30(2):299-309. doi:10.3233/JAD-2012-120106.
16. Zhu L, Gonzalez J. Modeling Floor Effects in Standardized Vocabulary Test Scores in a Sample of Low SES Hispanic Preschool Children under the Multilevel Structural Equation Modeling Framework. Front Psychol. 2017;8:2146. doi:10.3389/fpsyg.2017.02146.
17. Coombes R. Rationing of joint replacements raises fears of further cuts. BMJ. 2005;331(7528):1290. doi:10.1136/bmj.331.7528.1290.
18. Finer N. Rationing joint replacements: trust's decision seems to be based on prejudice or attributing blame. BMJ. 2005;331(7530):1472. doi:10.1136/bmj.331.7530.1472-a.
19. McNicol MW. Rationing joint replacements: ...and is false economy resulting in overall damage. BMJ. 2005;331(7530):1473. doi:10.1136/bmj.331.7530.1473.
20. Workgroup of the American Association of H, Knee Surgeons Evidence Based C. Obesity and total joint arthroplasty: a literature based review. J Arthroplasty. 2013;28(5):714-21. doi:10.1016/j.arth.2013.02.011.
21. Morris T, White I, Crowther M. Using simulation studies to evaluate statistical methods. Stat Med. 2019. doi:https://doi.org/10.1002/sim.8086.
22. Smith AJ, Dieppe P, Howard PW, Blom AW, National Joint Registry for E, Wales. Failure rates of metal-on-metal hip resurfacings: analysis of data from the National Joint Registry for England and Wales. Lancet. 2012;380(9855):1759-66. doi:10.1016/S0140-6736(12)60989-1.

For peer review only

23. Smith AJ, Dieppe P, Vernon K, Porter M, Blom AW, National Joint Registry of E et al. Failure rates of stemmed metal-on-metal hip replacements: analysis of data from the National Joint Registry of England and Wales. Lancet. 2012;379(9822):1199-204. doi:10.1016/S0140-6736(12)60353-5.

For peer review only

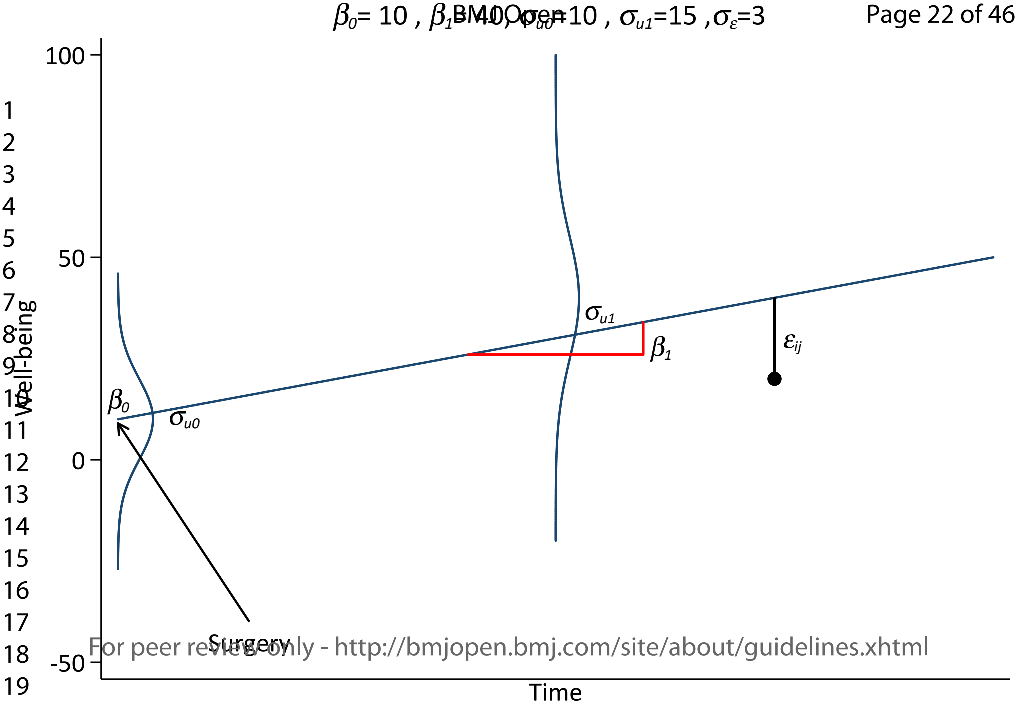

For peer review only - http://bmjopen.bmj.com/site/about/guidelines.xhtml

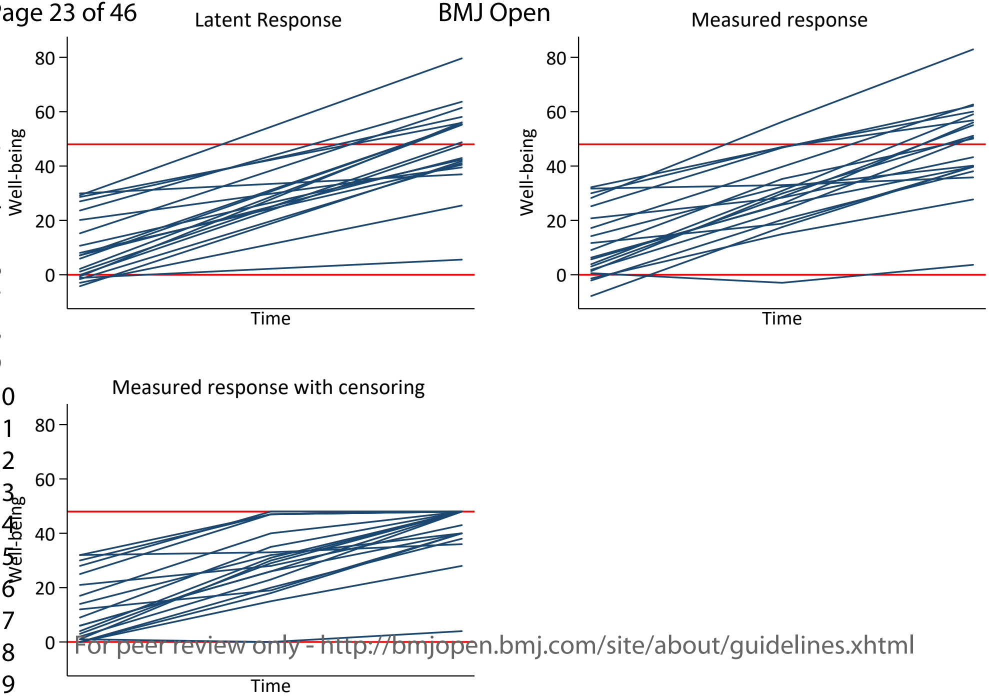

For peer review only - http://bmjopen.bmj.com/site/about/guidelines.xhtml

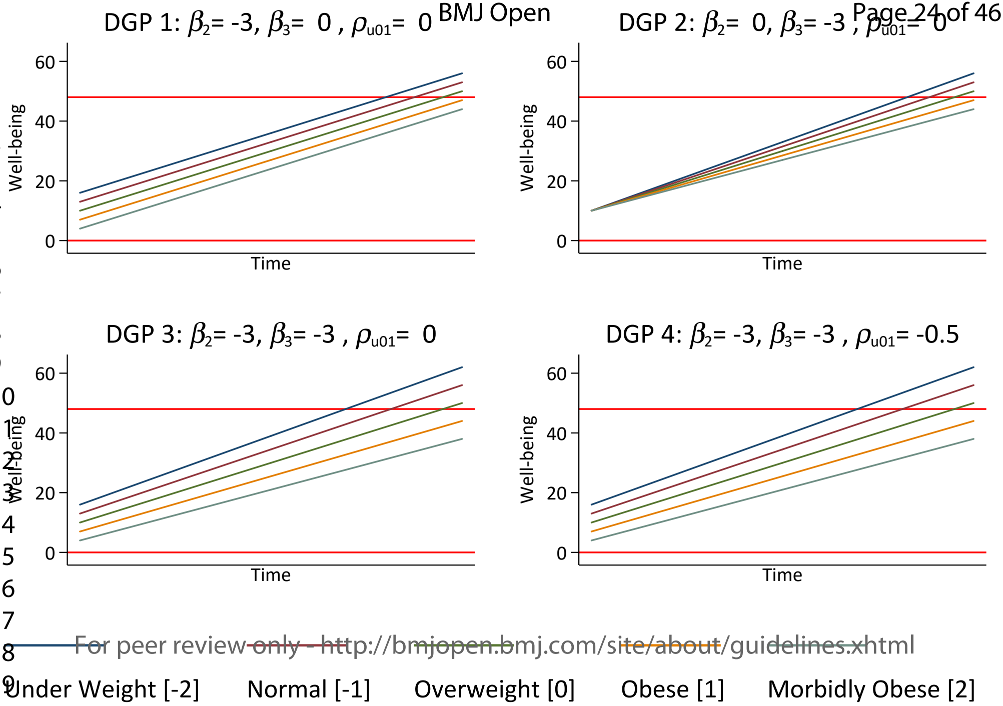

For peer review only - http://bmjopen.bmj.com/site/about/guidelines.xhtml

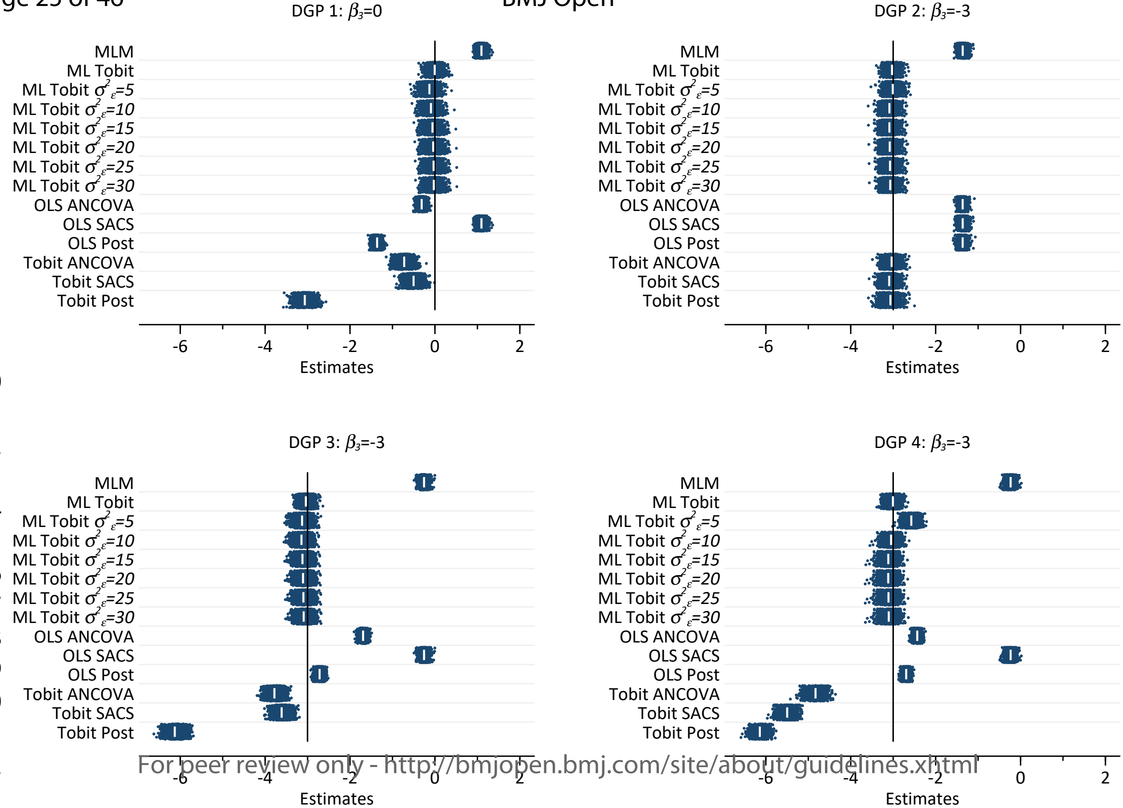

For peer review only - http://bmjopen.bmj.com/site/about/guidelines.xhtml

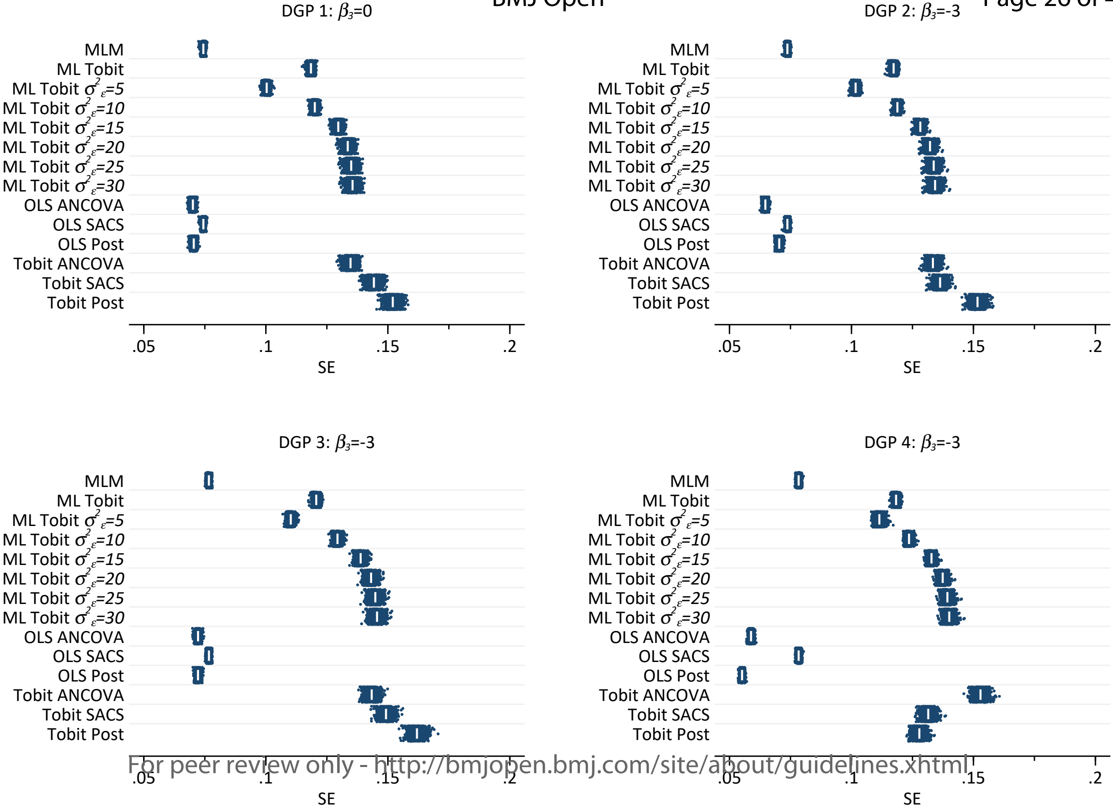

For peer review only - http://bmjopen.bmj.com/site/about/guidelines.xhtml

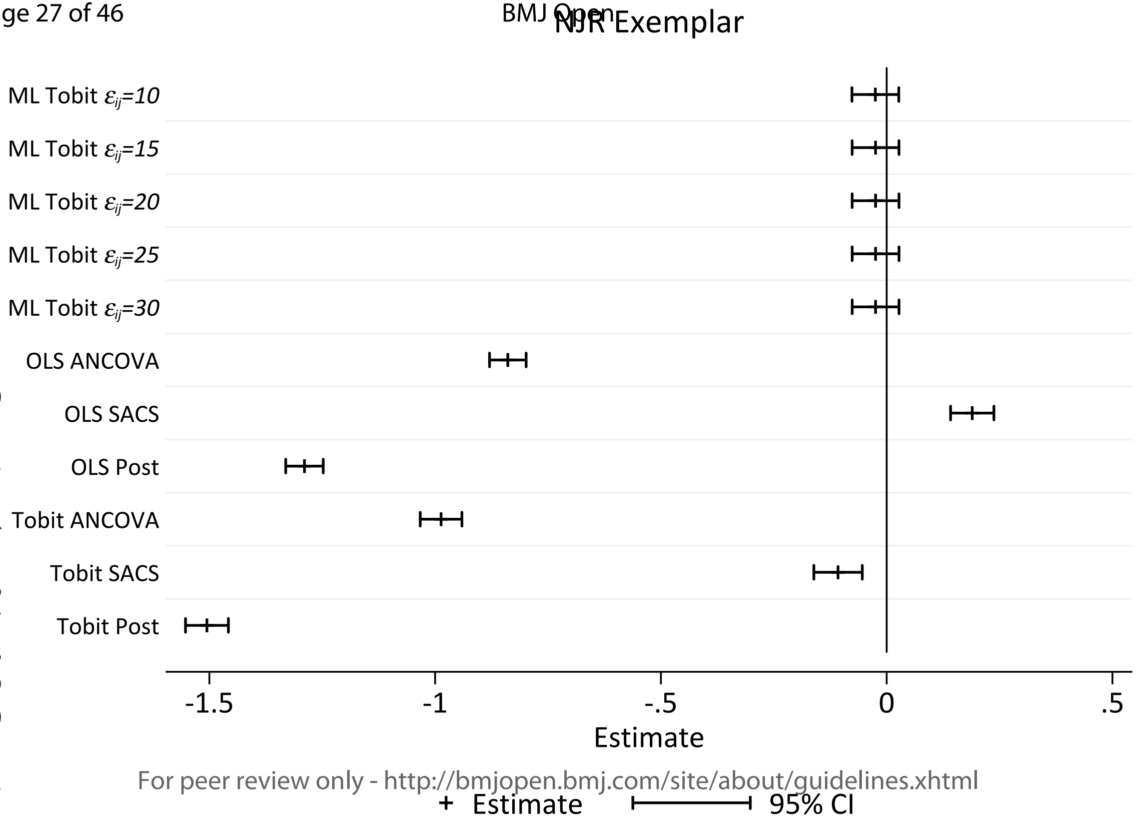
NJR Exemplar
ML Tobit εij=10
ML Tobit εij=15
ML Tobit εij=20
ML Tobit εij=25
ML Tobit εij=30
OLS ANCOVA
OLS SACS
OLS Post
Tobit ANCOVA
Tobit SACS
Tobit Post
-1.5
-1
-.5
0
.5
Estimate
Estimate
95% CI

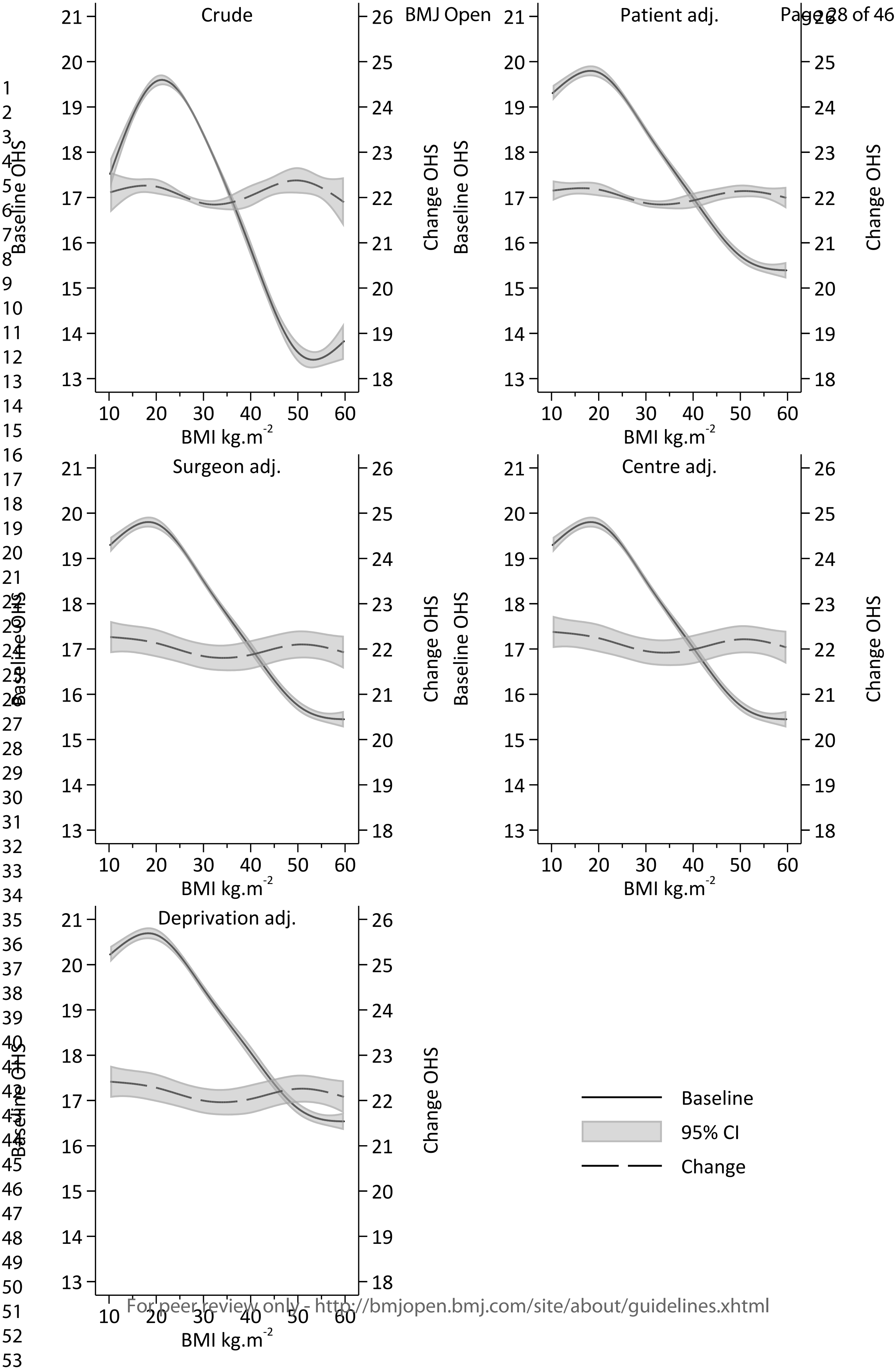
Crude
Patient adj.
Surgeon adj.
Centre adj.
Deprivation adj.
Baseline OHS
Change OHS
BMI kg.m^{-2}
Baseline
95% CI
Change

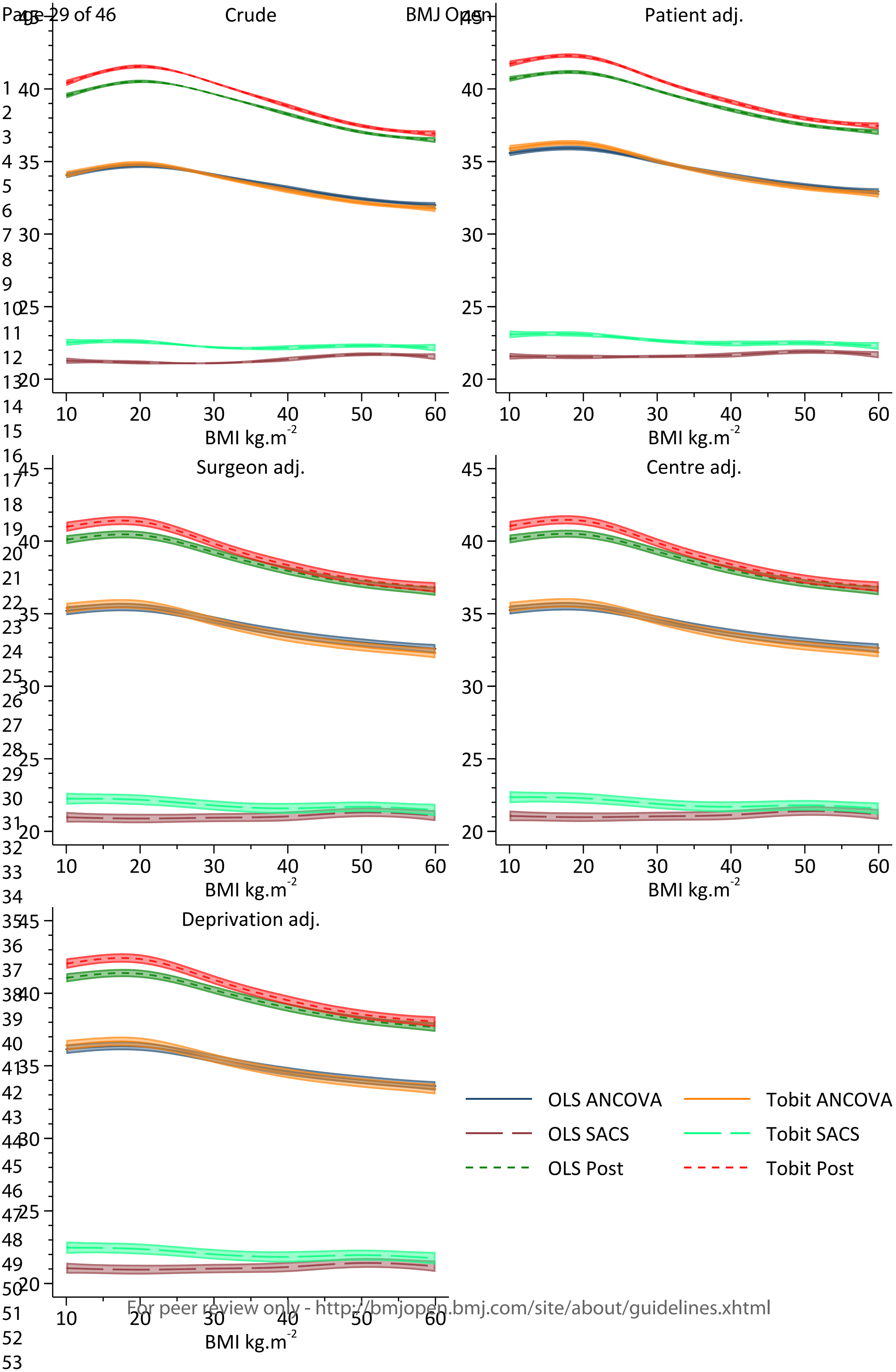
Crude
Patient adj.
Surgeon adj.
Centre adj.
Deprivation adj.
45
40
35
30
25
20
10
20
30
40
50
60
BMI kg.m-2
OLS ANCOVA
OLS SACS
OLS Post
Tobit ANCOVA
Tobit SACS
Tobit Post

Supplementary Figures

Supplementary.Figure 1: Inclusion / Exclusion Criteria of the NJR study.

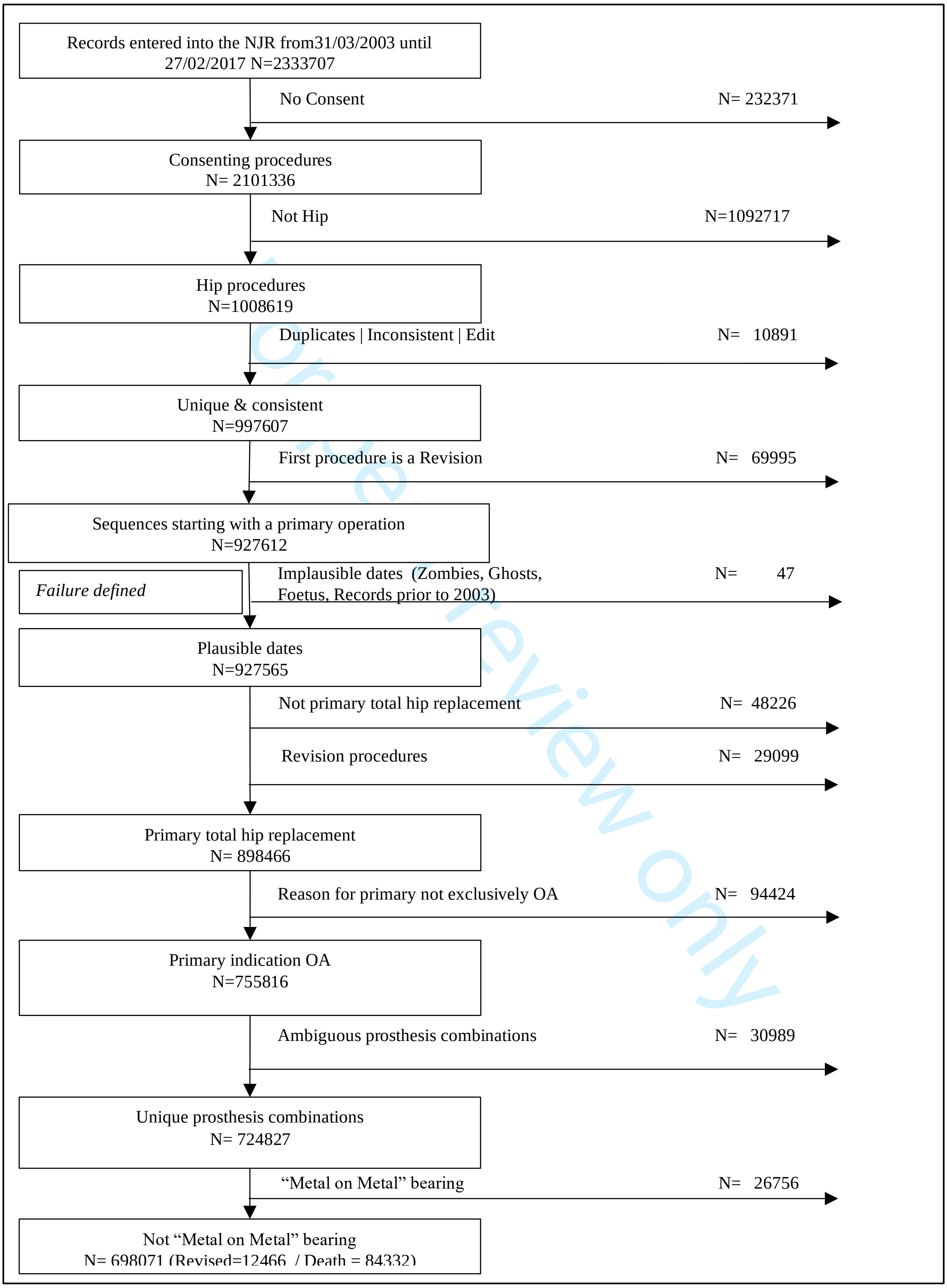

For peer review only

Supplementary.Figure 2: Description of covariate missing data in eligible data

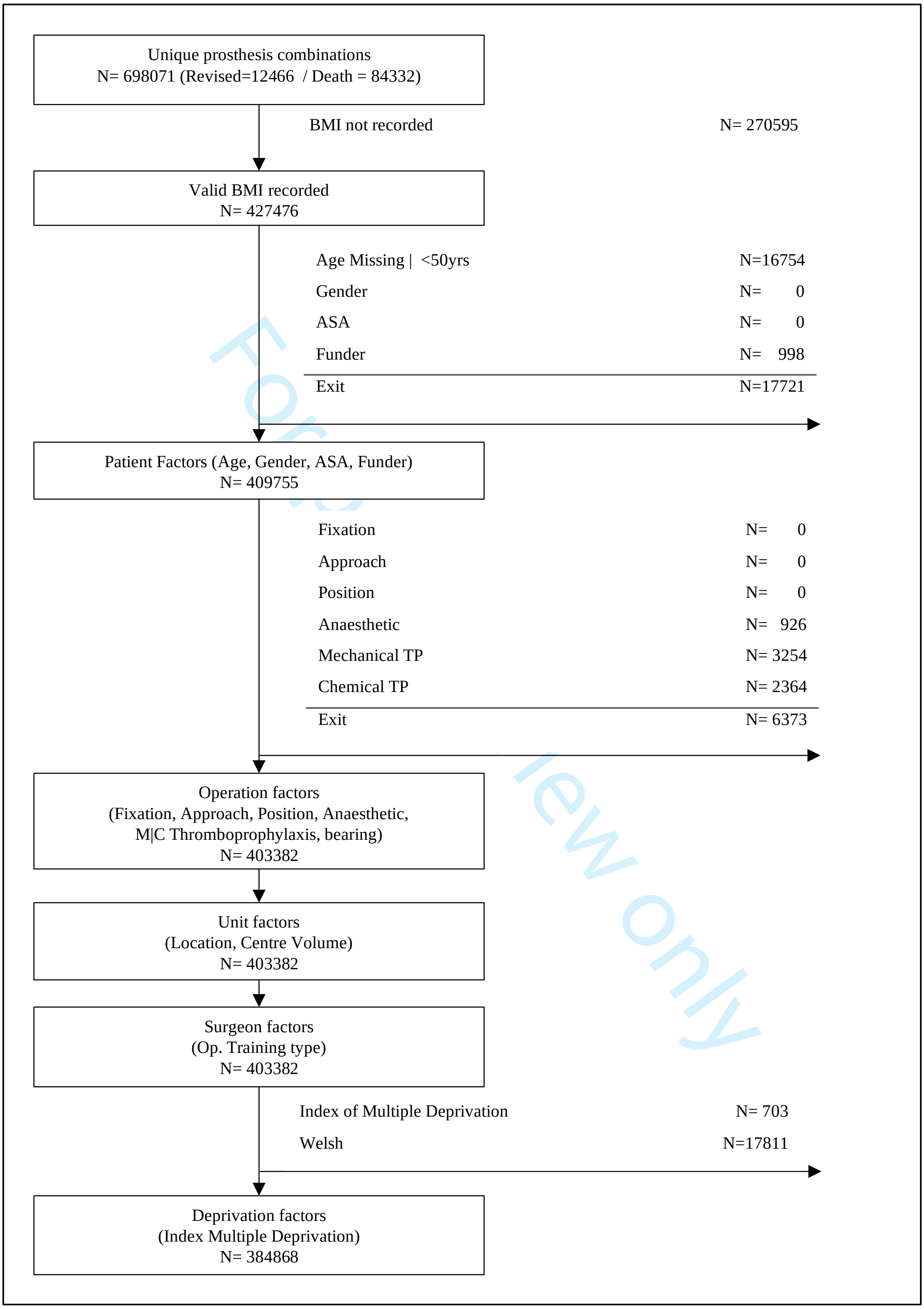

For peer review only

Supplementary.Figure 3: Description of National PROMS linkage to the NJR

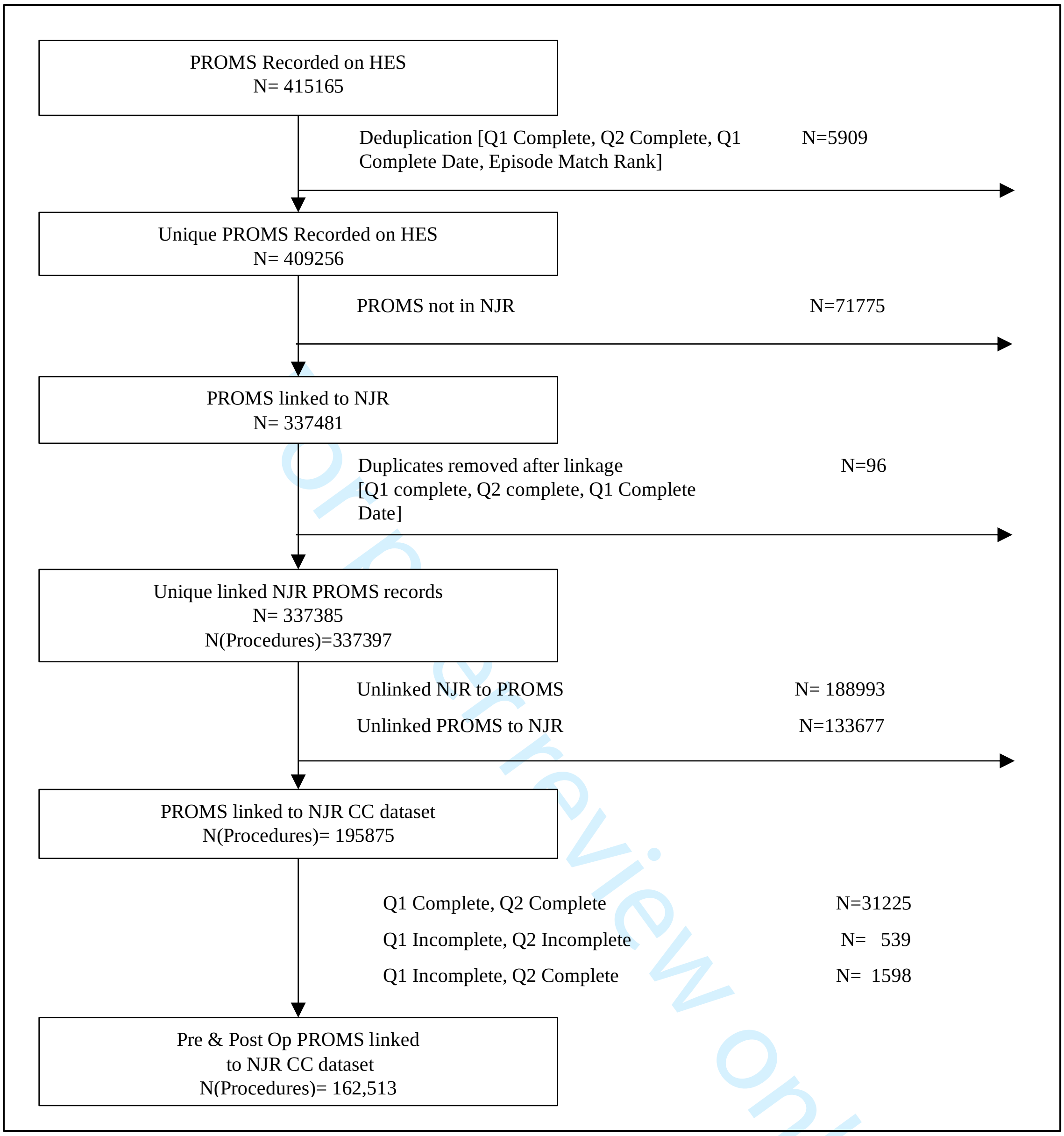

For peer review only

Supplementary.Figure 4: "Zip Plot" of the 1000 95% Confidence intervals for each method of analysis for DGP 1. The vertical axis is the centile of the two-sided p-value against H_0 : β_3=0 associated with the confidence interval for MLM and ML Tobit models.

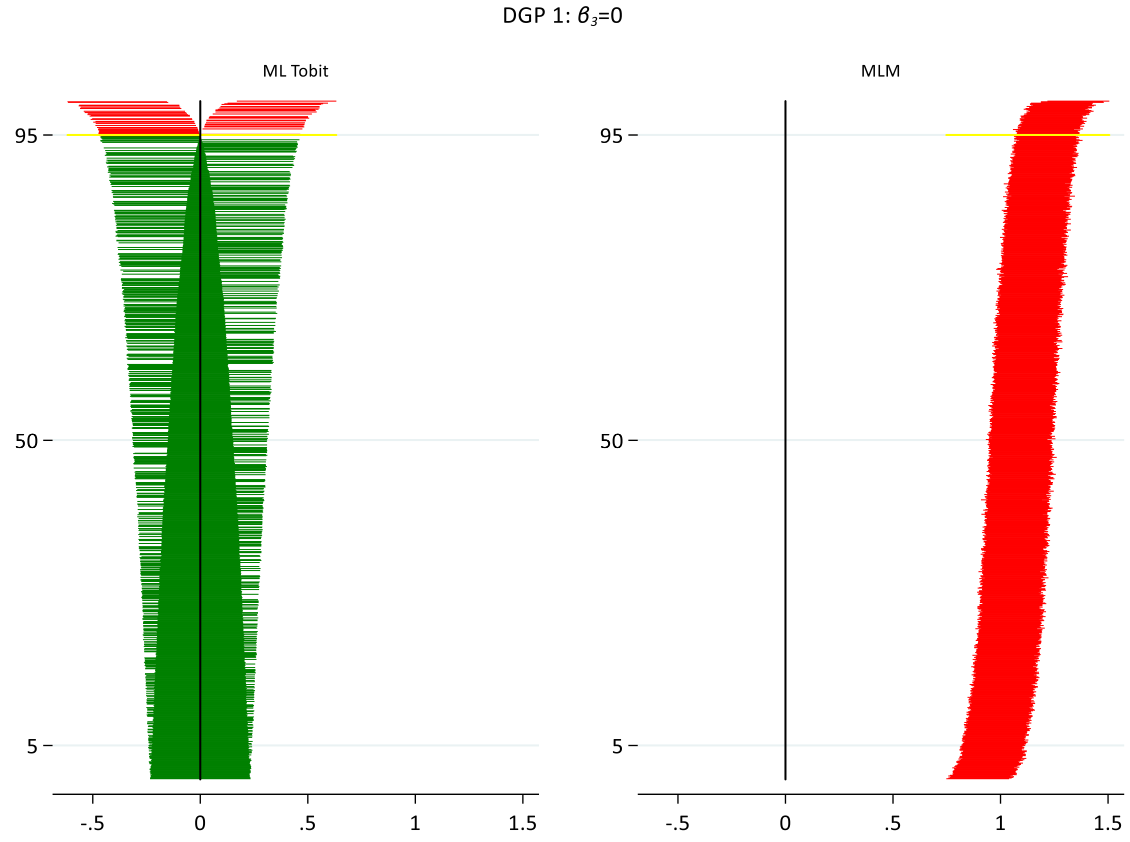

For peer review only - http://bmjopen.bmj.com/site/about/guidelines.xhtml

Supplementary.Figure 5: "Zip Plot" of the 1000 95% Confidence intervals for each method of analysis for DGP 1. The vertical axis is the centile of the two-sided p-value against H_0 : β_3=0 associated with the confidence interval for ML Tobit models with varying constraints of σ_{ε}^{2}

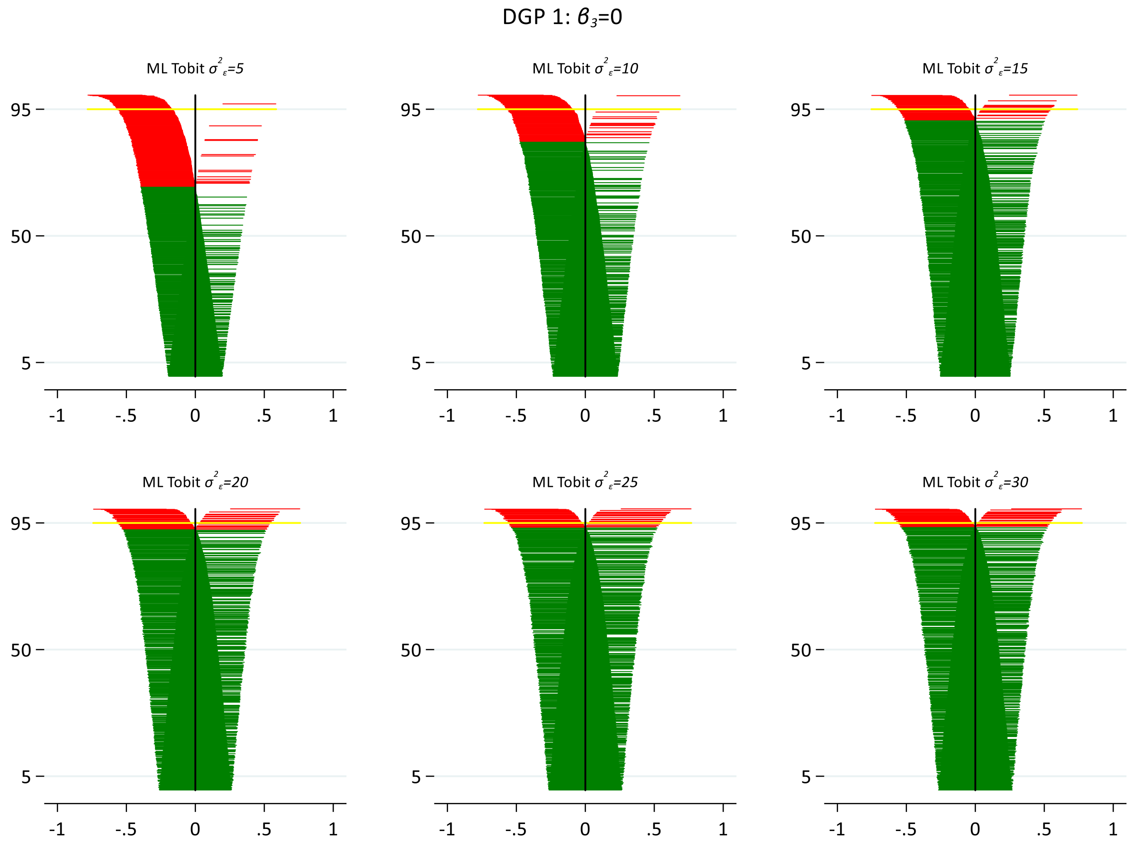

w only

Supplementary.Figure 6: "Zip Plot" of the 1000 95% Confidence intervals for each method of analysis for DGP 1. The vertical axis is the centile of the two-sided p-value against H_0 : β_3=0 associated with the confidence interval for Single level OLS and Tobit models.

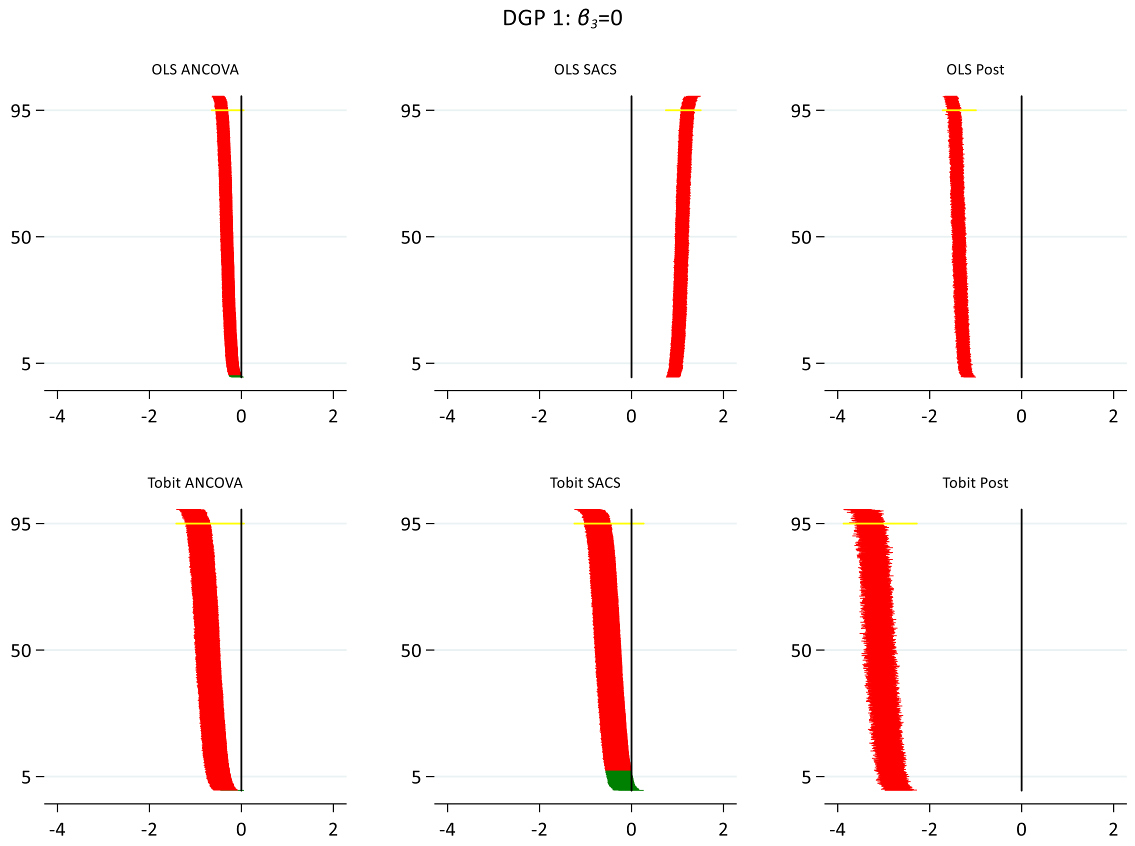

ew only

Supplementary.Figure 7: "Zip Plot" of the 1000 95% Confidence intervals for each method of analysis for DGP 2. The vertical axis is the centile of the two-sided p-value against H_0 : β_3=3 associated with the confidence interval for MLM and ML Tobit models.

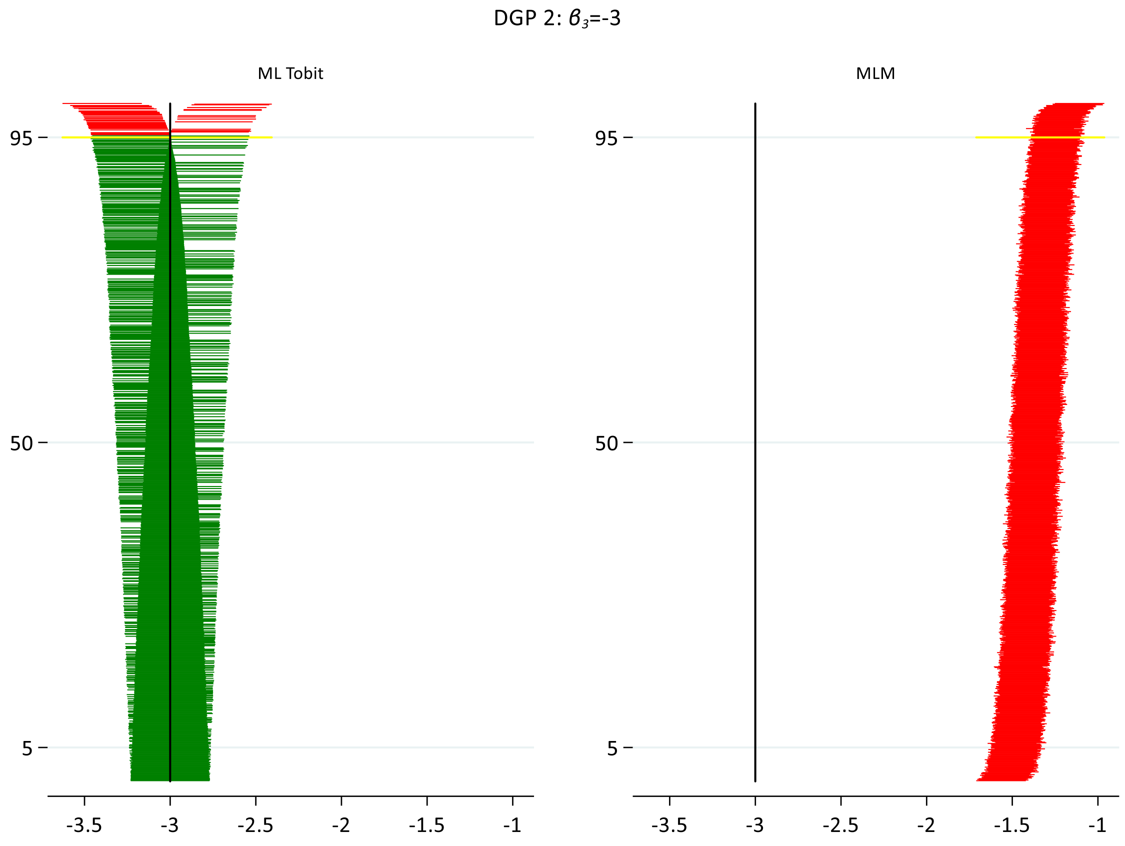

ew only

Supplementary.Figure 8: "Zip Plot" of the 1000 95% Confidence intervals for each method of analysis for DGP 2. The vertical axis is the centile of the two-sided p-value against H_0 : β_3=3 associated with the confidence interval for ML Tobit models with varying constraints of σ_ε^2

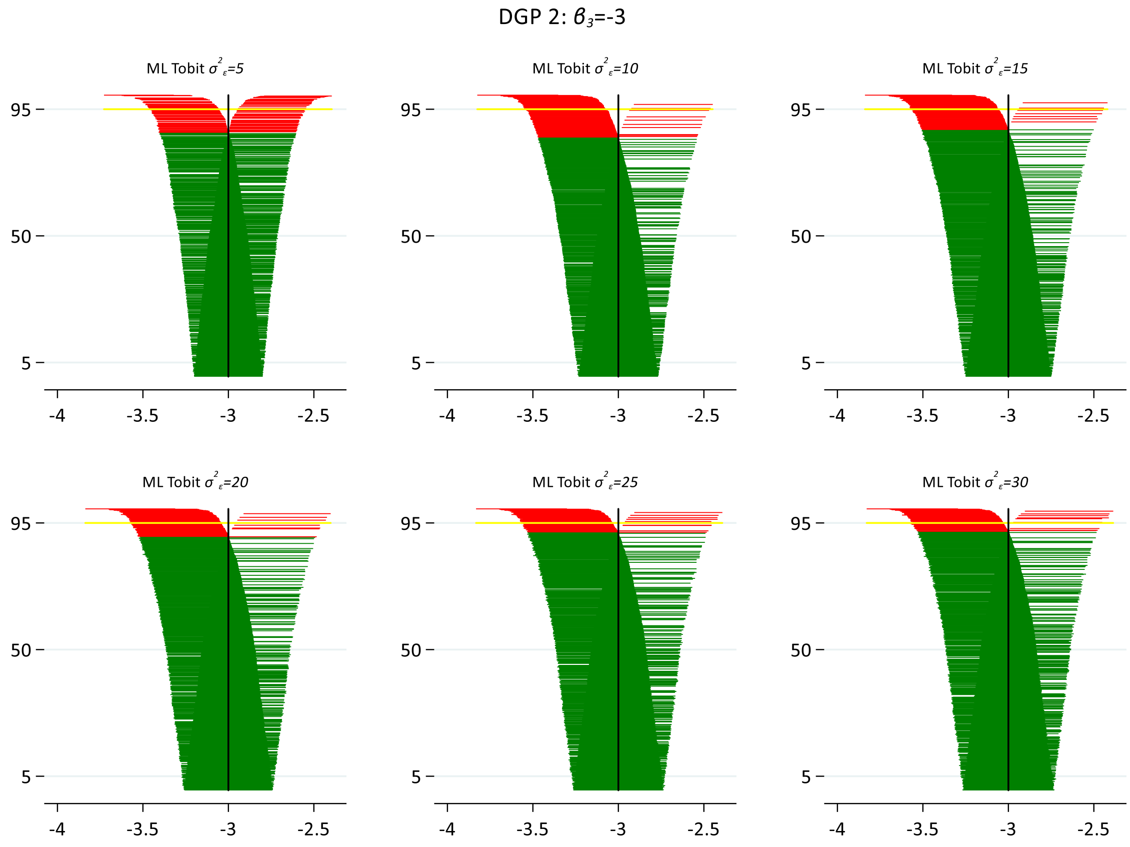

w only

Supplementary.Figure 9: "Zip Plot" of the 1000 95% Confidence intervals for each method of analysis for DGP 2. The vertical axis is the centile of the two-sided p-value against H_0 : β_3=3 associated with the confidence interval for Single level OLS and Tobit models.

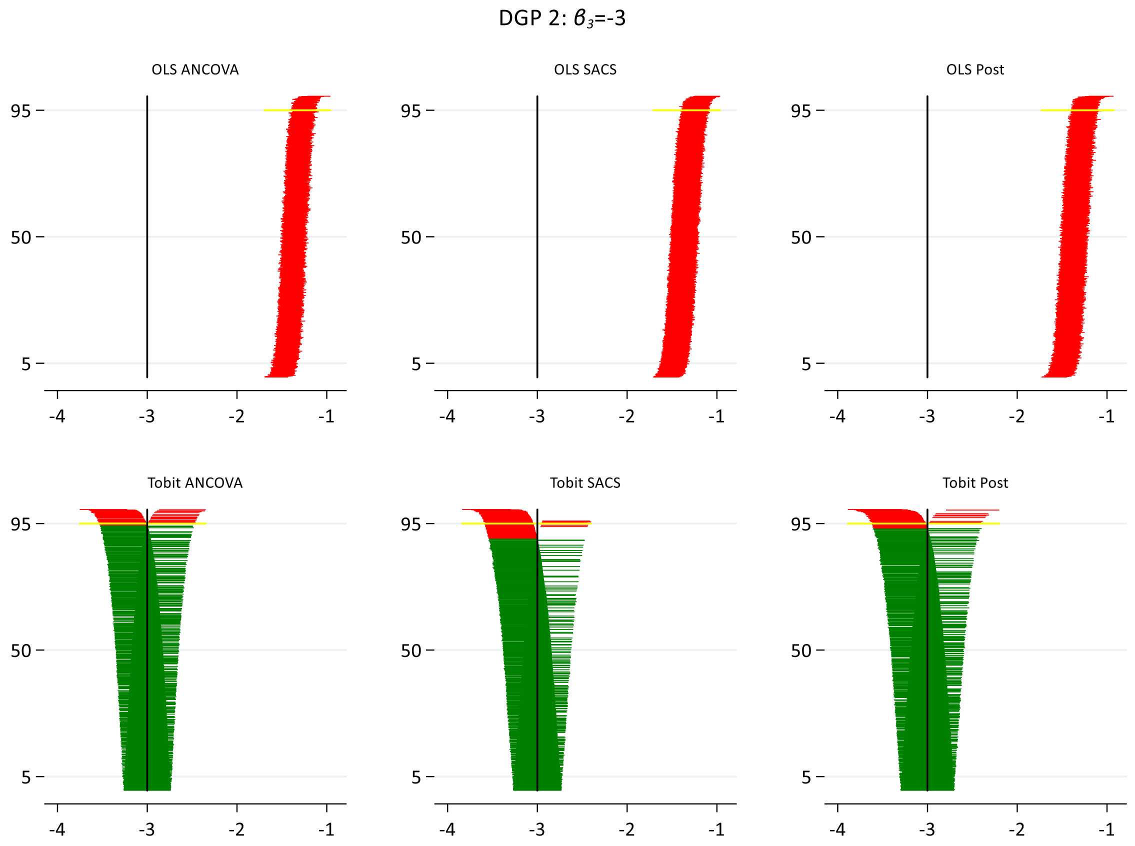

ew only

Supplementary.Figure 10: "Zip Plot" of the 1000 95% Confidence intervals for each method of analysis for DGP 3. The vertical axis is the centile of the two-sided p-value against H_0 : β_3=3 associated with the confidence interval for MLM and ML Tobit models.

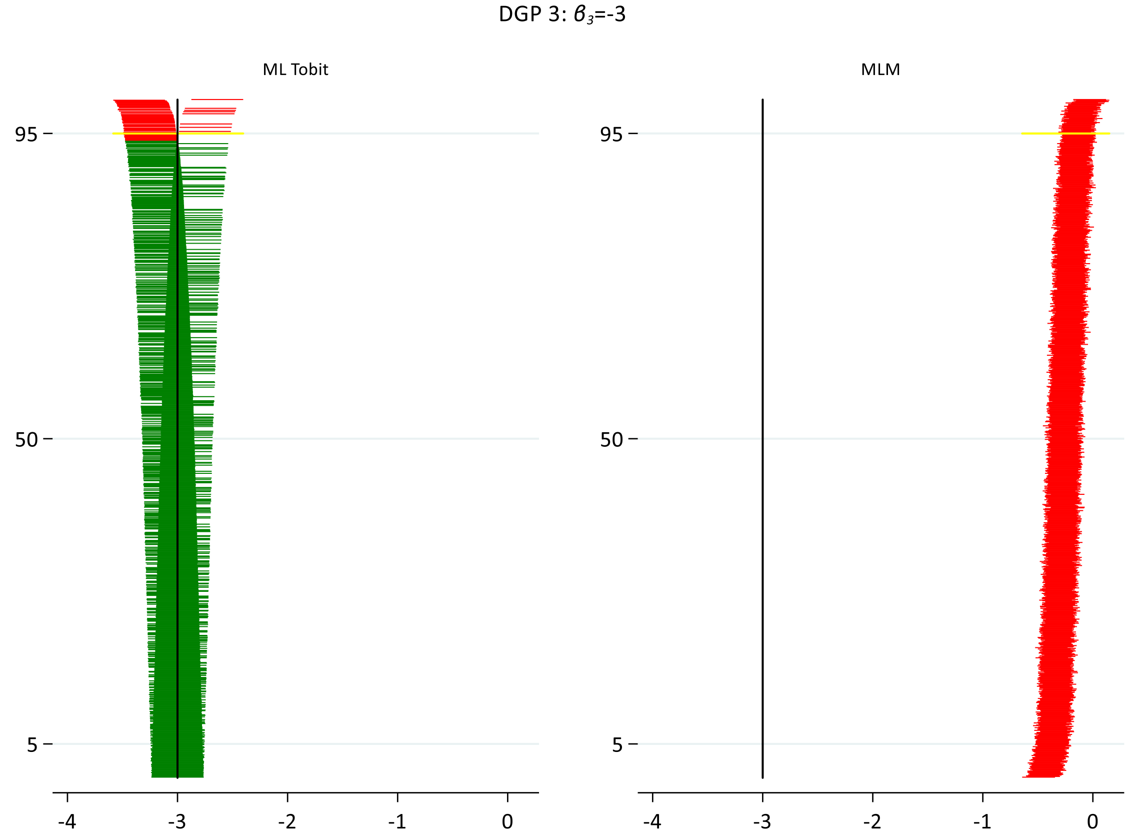

For peer review only - http://bmjopen.bmj.com/site/about/guidelines.xhtml

Supplementary.Figure 11: "Zip Plot" of the 1000 95% Confidence intervals for each method of analysis for DGP 3. The vertical axis is the centile of the two-sided p-value against H_0 : β_3=3 associated with the confidence interval for ML Tobit models with varying constraints of σ_ε^2

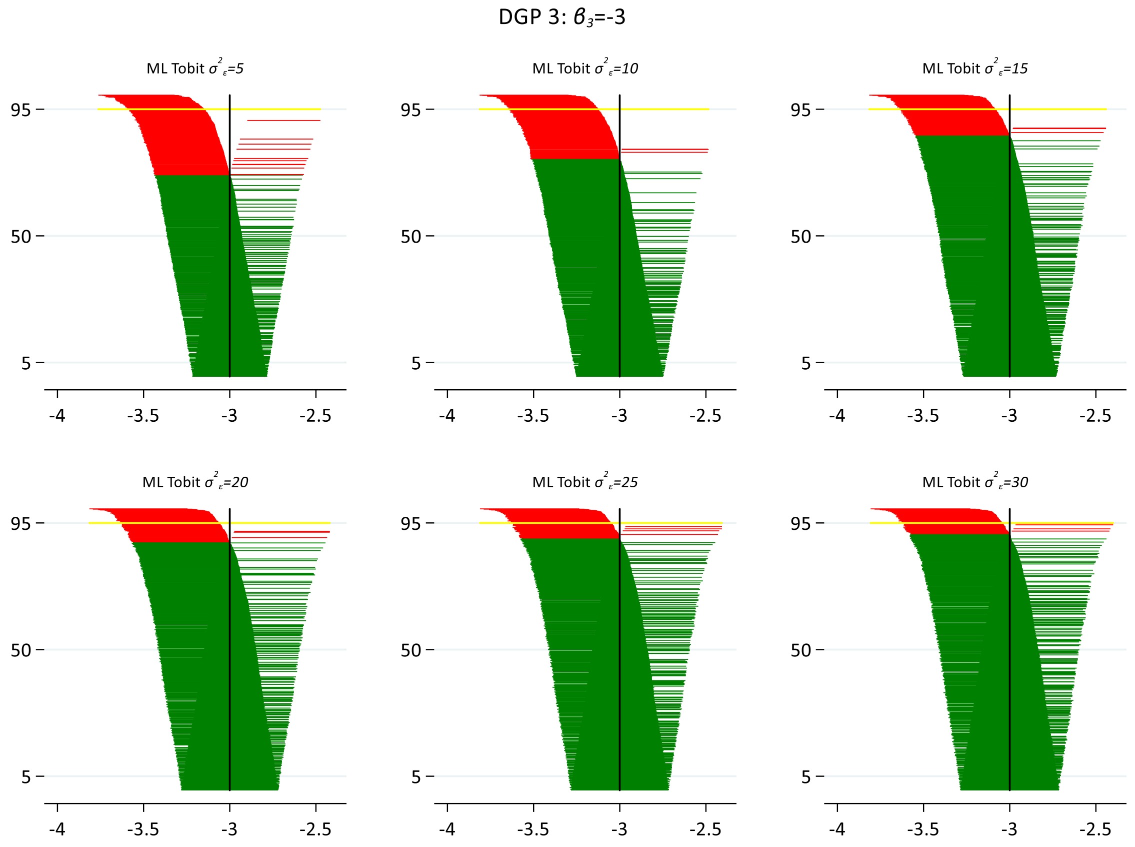

w only

Supplementary.Figure 12: "Zip Plot" of the 1000 95% Confidence intervals for each method of analysis for DGP 3. The vertical axis is the centile of the two-sided p-value against H_0 : β_3=3 associated with the confidence interval for Single level OLS and Tobit models.

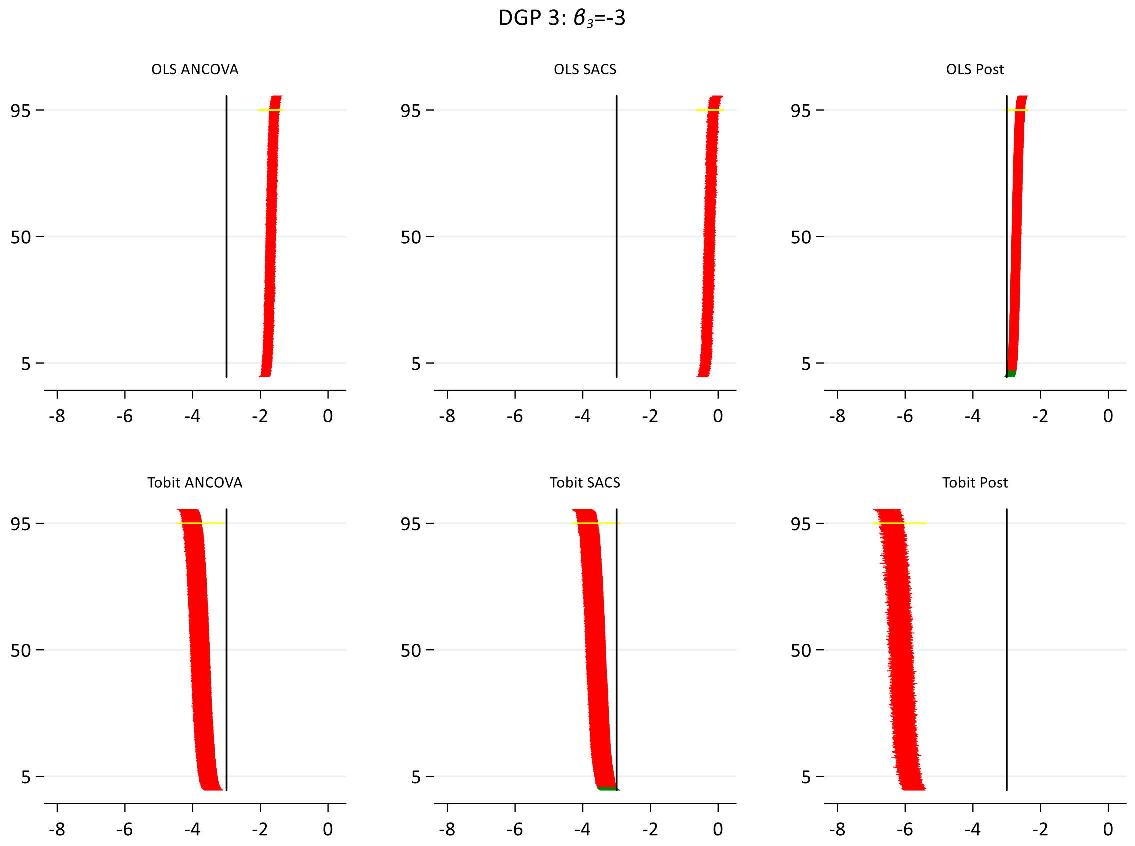

Supplementary.Figure 13: "Zip Plot" of the 1000 95% Confidence intervals for each method of analysis for DGP 4. The vertical axis is the centile of the two-sided p-value against H_0 : β_3=0 associated with the confidence interval for MLM and ML Tobit models.

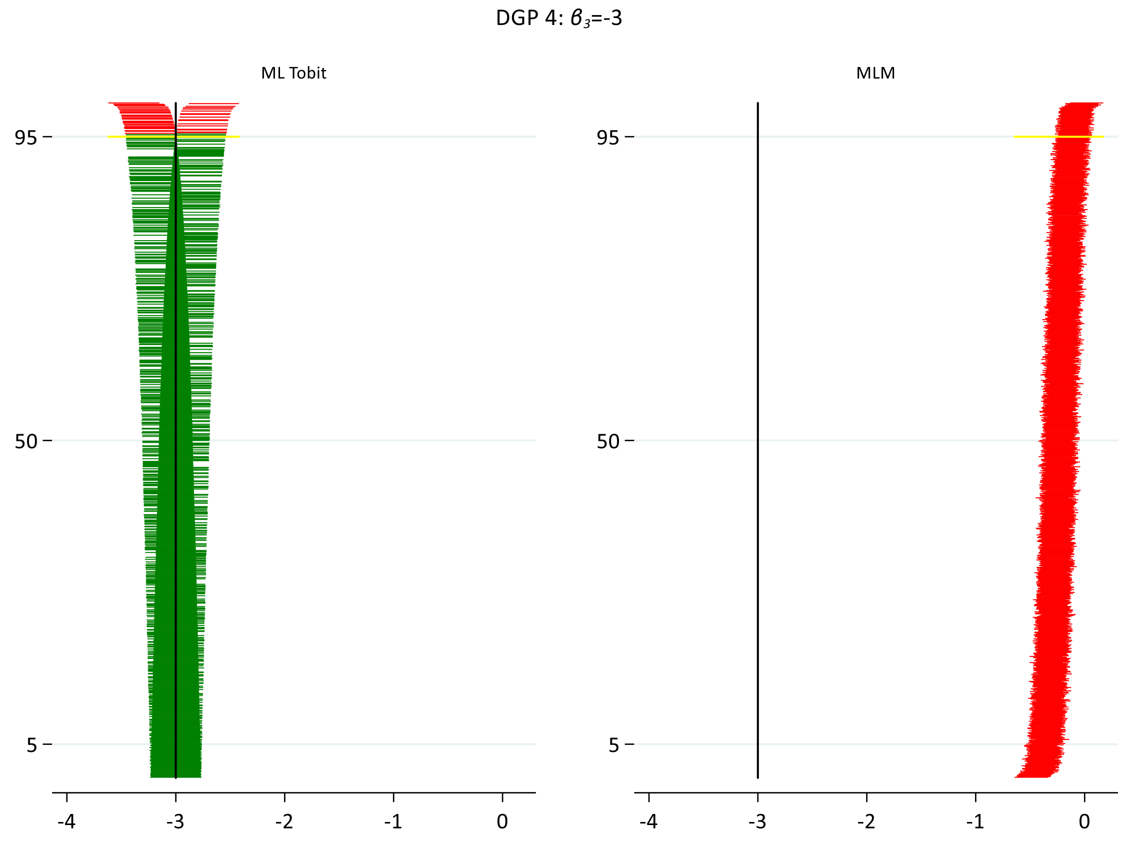

For peer review only - http://bmjopen.bmj.com/site/about/guidelines.xhtml

Supplementary.Figure 14: "Zip Plot" of the 1000 95% Confidence intervals for each method of analysis for DGP 4. The vertical axis is the centile of the two-sided p-value against H_0 : β_3=0 associated with the confidence interval for ML Tobit models with varying constraints of σ_ε^2

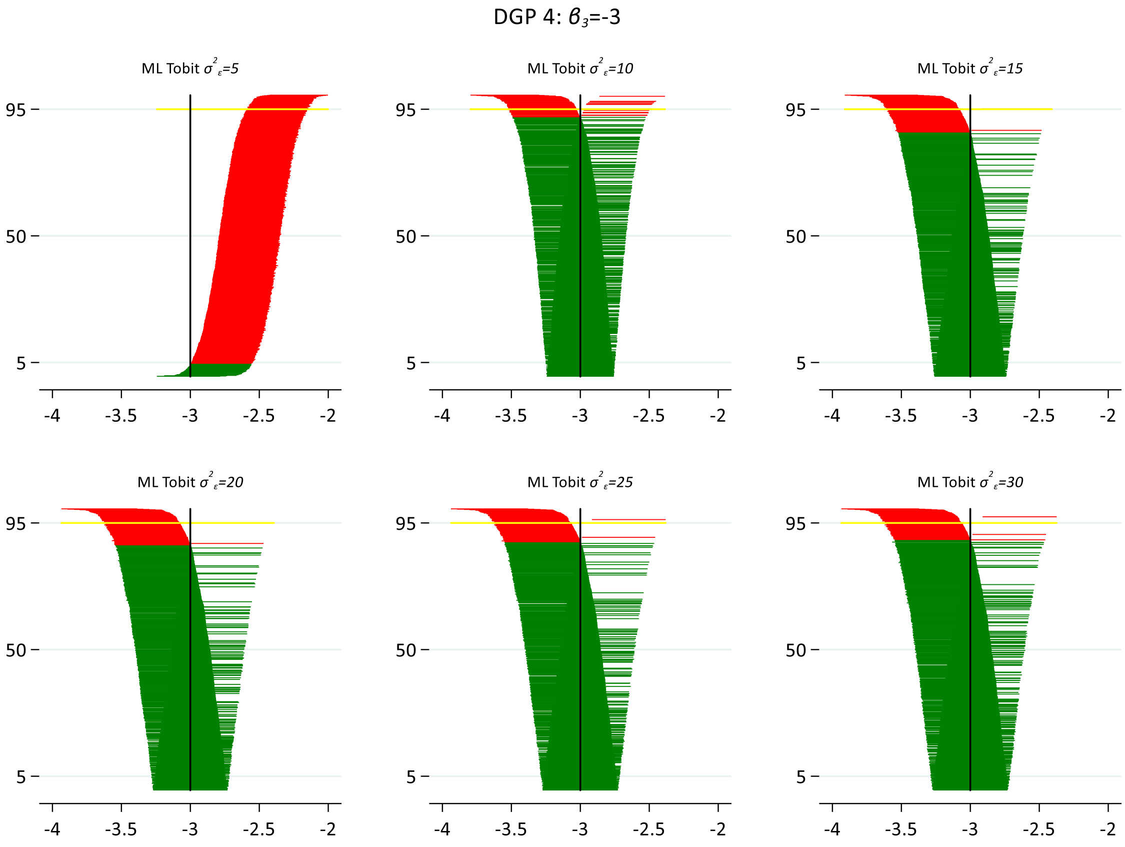

w only

Supplementary.Figure 15: "Zip Plot" of the 1000 95% Confidence intervals for each method of analysis for DGP 4. The vertical axis is the centile of the two-sided p-value against H_0 : β_3=0 associated with the confidence interval for Single-level OLS and Tobit models.

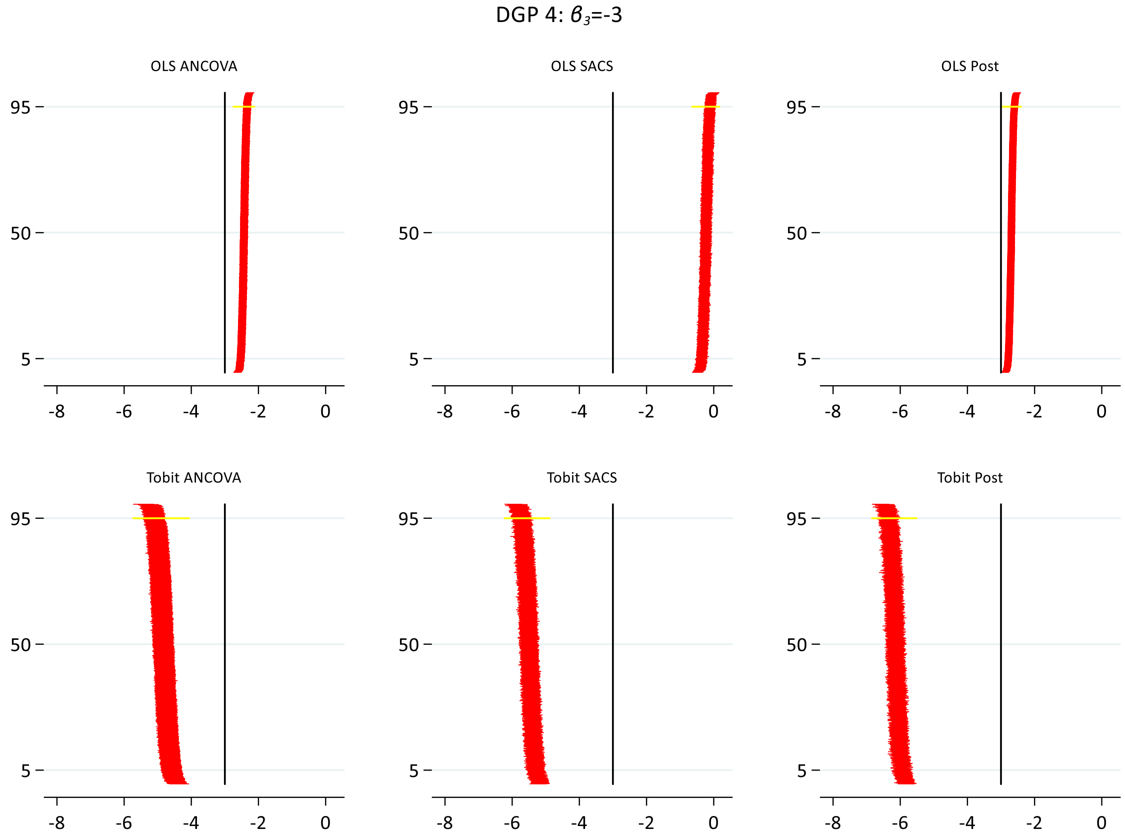

Supplementary.Figure 16: Marginal effect of level 1 error variance (σ_ε^2) constraint on level 2 variance components

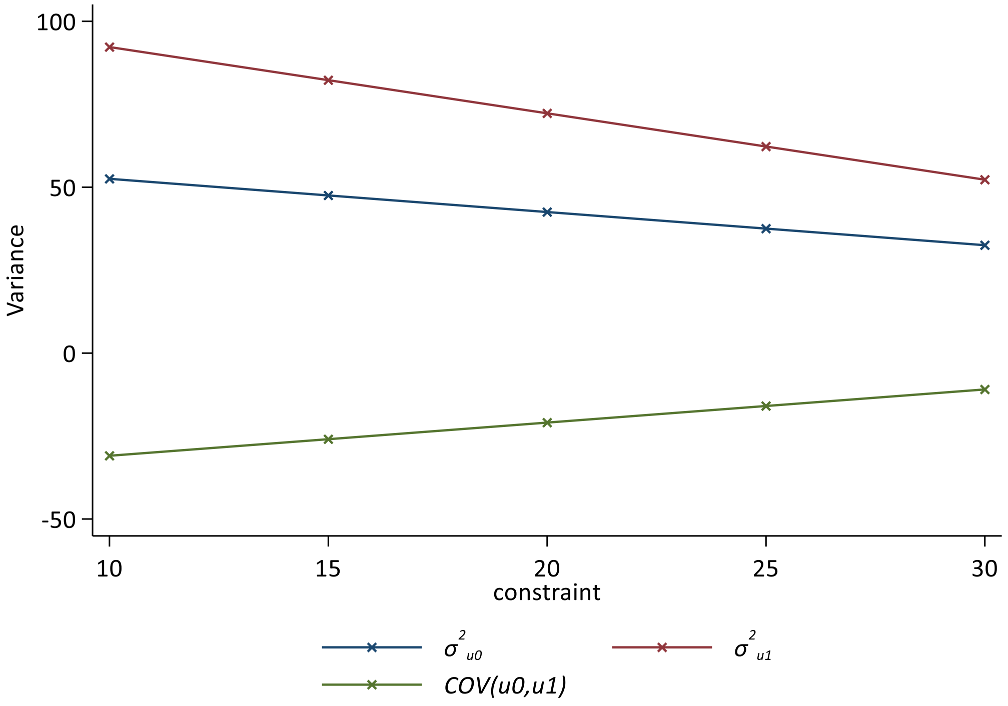

review only

Supplementary Tables

Supplementary.Table 1: Simulation estimates of performance characteristics (Monte Carlo Standarc Error in parantheses)

	Model	DGP 1: β_3 =0		DGP2 : β_3 =-3		DGP 3: β_3 =-3		DGP 4 : β_3 =-3	
Emperical SE	MLM	0.075	(0.0017)	0.072	(0.0016)	0.075	(0.0017)	0.079	(0.0018)
	ML Tobit	0.12	(0.0027)	0.12	(0.0026)	0.12	(0.0026)	0.12	(0.0026)
	ML Tobit σ_ε^2=5	0.14	(0.0031)	0.13	(0.0029)	0.14	(0.0032)	0.12	(0.0027)
	ML Tobit σ_ε^2 =10	0.14	(0.0031)	0.13	(0.003)	0.14	(0.0032)	0.13	(0.0029)
	ML Tobit σ_ε^2=15	0.14	(0.0031)	0.13	(0.003)	0.14	(0.0032)	0.14	(0.003)
	ML Tobit σ_ε^2=20	0.14	(0.0031)	0.13	(0.003)	0.14	(0.0032)	0.14	(0.0031)
	ML Tobit σ_ε^2=25	0.14	(0.0031)	0.13	(0.003)	0.14	(0.0032)	0.14	(0.0031)
	ML Tobit σ_ε^2=30	0.14	(0.0031)	0.13	(0.003)	0.14	(0.0032)	0.14	(0.0031)
	OLS SACS	0.075	(0.0017)	0.072	(0.0016)	0.075	(0.0017)	0.079	(0.0018)
	OLS ANCOVA	0.07	(0.0016)	0.064	(0.0014)	0.068	(0.0015)	0.059	(0.0013)
	OLS Post	0.072	(0.0016)	0.07	(0.0016)	0.071	(0.0016)	0.056	(0.0013)
	Tobit SACS	0.14	(0.0031)	0.13	(0.0029)	0.14	(0.0031)	0.15	(0.0034)
	Tobit ANCOVA	0.14	(0.0032)	0.13	(0.003)	0.14	(0.0032)	0.13	(0.003)
	Tobit Post	0.16	(0.0035)	0.15	(0.0034)	0.16	(0.0035)	0.13	(0.0028)
Mean Square Error	MLM	1.21	(0.0052)	2.69	(0.0075)	7.54	(0.013)	7.66	(0.014)
	ML Tobit	0.014	(0.0007)	0.014	(0.0006)	0.015	(0.0007)	0.014	(0.0006)
	ML Tobit σ_ε^2=5	0.036	(0.0015)	0.017	(0.0008)	0.038	(0.0015)	0.2	(0.0033)
	ML Tobit σ_ε^2=10	0.028	(0.0012)	0.025	(0.0011)	0.041	(0.0015)	0.019	(0.0009)
	ML Tobit σ_ε^2=15	0.023	(0.001)	0.026	(0.0011)	0.035	(0.0013)	0.032	(0.0014)
	ML Tobit σ_ε^2=20	0.021	(0.0009)	0.024	(0.001)	0.032	(0.0013)	0.033	(0.0014)
	ML Tobit σ_ε^2=25	0.02	(0.0009)	0.023	(0.001)	0.03	(0.0012)	0.032	(0.0014)
	ML Tobit σ_ε^2=30	0.02	(0.0009)	0.022	(0.001)	0.029	(0.0012)	0.031	(0.0014)
	OLS SACS	1.21	(0.0052)	2.69	(0.0075)	7.54	(0.013)	7.66	(0.014)
	OLS ANCOVA	0.1	(0.0014)	2.68	(0.0066)	1.71	(0.0056)	0.33	(0.0021)
	OLS Post	1.86	(0.0062)	2.68	(0.0073)	0.085	(0.0013)	0.097	(0.0011)
	Tobit SACS	0.54	(0.0064)	0.018	(0.0008)	0.63	(0.0069)	3.37	(0.018)
	Tobit ANCOVA	0.27	(0.0046)	0.027	(0.0011)	0.39	(0.0056)	6.25	(0.021)
	Tobit Post	9.42	(0.03)	0.027	(0.0012)	9.81	(0.031)	9.88	(0.025)
Relative Error	MLM	-1.31	(2.21)	1.98	(2.28)	2.74	(2.3)	-0.31	(2.23)
	ML Tobit	-0.95	(2.22)	0.79	(2.25)	2.37	(2.29)	1.66	(2.27)
	ML Tobit σ_ε^2=5	-28.5	(1.6)	-22.8	(1.73)	-23.8	(1.71)	-7.01	(2.08)
	ML Tobit σ_ε^2=10	-14.5	(1.91)	-10.3	(2.01)	-8.8	(2.04)	-3.97	(2.15)
	ML Tobit σ_ε^2=15	-7.35	(2.07)	-3.54	(2.16)	-2.41	(2.18)	-2.46	(2.18)
	ML Tobit σ_ε^2=20	-4.29	(2.14)	-0.57	(2.22)	0.26	(2.24)	-1.4	(2.21)
	ML Tobit σ_ε^2=25	-3.04	(2.17)	0.65	(2.25)	1.35	(2.27)	-0.8	(2.22)
	ML Tobit σ_ε^2=30	-2.51	(2.18)	1.17	(2.26)	1.83	(2.28)	-0.47	(2.23)
	OLS SACS	-1.3	(2.21)	1.99	(2.28)	2.75	(2.3)	-0.3	(2.23)
	OLS ANCOVA	-0.5	(2.23)	1.51	(2.27)	6.84	(2.39)	-0.54	(2.23)
	OLS Post	-1.96	(2.19)	-0.086	(2.24)	1.46	(2.27)	-1.25	(2.21)

For peer review only

	Tobit SACS	-2.97	(2.17)	2.09	(2.28)	2.82	(2.3)	-0.056	(2.24)
	Tobit ANCOVA	-0.19	(2.23)	2.21	(2.29)	4.01	(2.33)	-1.35	(2.21)
	Tobit Post	-2.82	(2.17)	1.27	(2.27)	2.74	(2.3)	0.83	(2.26)
Relative Precision	MLM	247	(14.1)	232.3	(13.9)	275.4	(16.9)	132.5	(6.66)
	ML Tobit	37.8	(4.36)	28.3	(4.07)	50.5	(5.64)	6.08	(3.13)
	ML Tobit σ_ε^2=5								
	ML Tobit σ_ε^2=10	-0.034	(0.97)	-0.92	(1.07)	3.91	(1.24)	-13.3	(1.5)
	ML Tobit σ_ε^2=15	0.62	(1.22)	-1.66	(1.37)	3.24	(1.52)	-22.5	(1.68)
	ML Tobit σ_ε^2=20	1.22	(1.3)	-1.7	(1.44)	2.57	(1.6)	-26	(1.7)
	ML Tobit σ_ε^2=25	1.67	(1.33)	-1.52	(1.47)	2.31	(1.62)	-27.2	(1.7)
	ML Tobit σ_ε^2=30	1.99	(1.34)	-1.34	(1.47)	2.29	(1.63)	-27.5	(1.69)
	OLS SACS	247	(14.1)	232.3	(13.9)	275.4	(16.9)	132.5	(6.66)
	OLS ANCOVA	297.2	(16)	327.4	(14.8)	357.6	(20.9)	310.3	(18.8)
	OLS Post	281.7	(15.5)	250	(14.8)	312.9	(19.2)	360.1	(21.2)
	Tobit SACS	2.26	(1.94)	1.8	(1.89)	7.48	(2.72)	-38.6	(2.12)
	Tobit ANCOVA	-5.67	(3.07)	-2.33	(1.55)	1.52	(3.56)	-19.1	(3.59)
	Tobit Post	-19.5	(2.6)	-22.5	(2.6)	-15.9	(2.95)	-10.6	(4.08)

For peer review only

Analysis of change in patient reported outcome measures with floor and ceiling effects using the multi-level Tobit model: A simulation study and an example from a National Joint Register using body mass index and the Oxford Hip Score.

Journal:	*BMJ Open*
Manuscript ID	bmjopen-2019-033646.R1
Article Type:	Original research
Date Submitted by the Author:	22-Jun-2020
Complete List of Authors:	Sayers, Adrian; University of Bristol, Muscloskeletal Research Unit<br>Whitehouse, Michael; University of Bristol, Musculoskeletal Research Unit<br>Judge, Andrew; University of Bristol, Musculoskeletal Research Unit, Translational Health Sciences, Bristol Medical School, University of Bristol, Learning and Research Building, Level 1, Southmead Hospital, Southmead, BS10 5NB<br>MacGregor, Alex; University of East Anglia, Norwich Medical School<br>Blom, AW; University of Bristol,<br>Ben-Shlomo, Yoav; University of Bristol,
**Primary Subject Heading**:	Epidemiology
Secondary Subject Heading:	Patient-centred medicine, Rheumatology
Keywords:	Multi-level Tobit Model, Change Scores, Epidemiologic Methods, Arthroplasty, Patient Reported Outcome Measures, Longitudinal Studies

SCHOLARONE™
Manuscripts

I, the Submitting Author has the right to grant and does grant on behalf of all authors of the Work (as defined in the below author licence), an exclusive licence and/or a non-exclusive licence for contributions from authors who are: i) UK Crown employees; ii) where BMJ has agreed a CC-BY licence shall apply, and/or iii) in accordance with the terms applicable for US Federal Government officers or employees acting as part of their official duties; on a worldwide, perpetual, irrevocable, royalty-free basis to BMJ Publishing Group Ltd ("BMJ") its licensees and where the relevant Journal is co-owned by BMJ to the co-owners of the Journal, to publish the Work in this journal and any other BMJ products and to exploit all rights, as set out in our licence.

The Submitting Author accepts and understands that any supply made under these terms is made by BMJ to the Submitting Author unless you are acting as an employee on behalf of your employer or a postgraduate student of an affiliated institution which is paying any applicable article publishing charge ("APC") for Open Access articles. Where the Submitting Author wishes to make the Work available on an Open Access basis (and intends to pay the relevant APC), the terms of reuse of such Open Access shall be governed by a Creative Commons licence – details of these licences and which Creative Commons licence will apply to this Work are set out in our licence referred to above.

Other than as permitted in any relevant BMJ Author's Self Archiving Policies, I confirm this Work has not been accepted for publication elsewhere, is not being considered for publication elsewhere and does not duplicate material already published. I confirm all authors consent to publication of this Work and authorise the granting of this licence.

For peer review only

Analysis of change in patient reported outcome measures with floor and ceiling effects using the multi-level Tobit model: A simulation study and an example from a National Joint Register using body mass index and the Oxford Hip Score.

Adrian Sayers[a,b], Michael R Whitehouse[a,c], Andrew Judge[a,c], Alexander J Macgregor[d], Ashley W Blom[a,c], Yoav Ben-Shlomo[b].

Author affiliations:

a) Musculoskeletal Research Unit, Bristol Medical School, 1st Floor Learning & Research Building, Southmead Hospital, Bristol, BS10 5NB, United Kingdom
b) Population Health Sciences, Bristol Medical School, Canynge Hall, 39 Whatley Road Bristol, BS8 2PS, United Kingdom
c) National Institute for Health Research Bristol Biomedical Research Centre, University of Bristol
d) Norwich Medical School, University of East Anglia, Norwich, United Kingdom

Running head : Analysis of change with multi-level Tobit models

Conflict of Interest: The authors have no conflict of interests

Financial Support

AS is funded by an MRC Strategic Skills Fellowship MR/L01226X/1

This study was supported by the NIHR Biomedical Research Centre at University Hospitals Bristol NHS Foundation Trust and the University of Bristol. The views expressed in this publication are those of the author(s) and not necessarily those of the NHS, the National Institute for Health Research or the Department of Health and Social Care.

Corresponding Author: Adrian Sayers

email address for correspondence, adrian.sayers@bristol.ac.uk

For peer review only

Abstract (290 Words)

Objectives

This study has three objectives. 1) Investigate the association between body mass index (BMI) and the efficacy of primary hip replacement using a Patient Reported Outcome Measure (PROMs) with a measurement floor and ceiling. 2) Explore the performance of different estimation methods to estimate change in PROMs score following surgery using a simulation study and real word data where data has measurement floors and ceilings. 3) Lastly, develop guidance for practicing researchers on the analysis of PROMs in the presence of floor and ceiling effects.

Design

Simulation study and prospective national medical device regiseter

Setting

National register of joint replacement and medical devices

Methods

Using a Monte-Carlo simulation study and data from a national joint replacement register (162,513 patients with pre/post surgery PROMs) we investigate simple approaches for the analysis of outcomes with floor and ceiling effects that are measured at two occasions: linear and Tobit regression (baseline adjusted ANCOVA, change-score analysis, post-score analysis) in addition to linear and multi-level Tobit models.

Primary outcome

The primary outcome of interest is change in patient reported outcome measures from pre-surgery to 6 months post-surgery.

Results

Analysis of data with floor and ceiling effects with models that fail to account for these features induce substantial bias. Single level Tobit models only correct for floor or ceiling effects when the exposure of interest is not associated with the baseline score. In observational data scenarios, only multi-level Tobit models are capable of providing unbiased inferences.

Conclusions

Inferences from pre/post studies that fail to account for floor and ceiling effects may induce spurious associations with substantial risk of bias. Multi-level Tobit models indicate the efficacy of total hip replacement is independent of BMI. Restricting access to total hip replacement based on a patients BMI can not be supported by the data.

For peer review only

Strengths and limitations of this study

- We use a comprehensive simulation study and large prospective study set to investigate the effect of floor and ceiling effects in the analysis of change in patient reported outcome measure pre- and post- surgery.
- We demonstrate the use and performance of mutli-level Tobit models to estimate change in patient reported outcome measures with floor and ceiling effects and compare them to simple analytical approaches.
- We compare and demonstrate a variety of estimators in simulation under a variety of different data generating mechanisms and compare results to real world data.
- This is the largest and most comprehensive analysis of the effect of BMI on the efficiacy of total hip replacement and provides data which will influence the provision of hip replacement.

Keywords

Multi-level Tobit Model, Change Scores, Epidemiologic Methods, Arthroplasty, Patient Reported Outcome Measures, Longitudinal Studies

For peer review only

Introduction

In many non-randomised experiments, researchers are interested in assessing how change in health status is associated with a covariate of interest. Whilst there is much guidance available on assessing change in randomised experiments, and extensive discussion with respect to efficiency and bias [1-9], the guidance in non-randomised studies is less clear. The principle difference is that in observational studies we do not expect balance between different levels of an exposure at baseline, in addition to expecting imbalance in other confounding factors. Glymour et al. advocate the use of, simple analysis of, change scores (SACS) without baseline adjustment to achieve unbiased causal effect estimates using causal arguments presented through Directed Acyclic Graphs (DAGS) [10]. They briefly suggest that in settings with floor and/or ceiling effects, that standard change analyses with and without baseline adjustment are both biased, and non-standard analyses based on Tobit models (censored regression) may ameliorate floor and ceiling problems. The degree to which Tobit models ameliorate the problems caused by floor and ceiling effects is unclear. Some authors suggest that using percentage change is one strategy to avoid dealing with floor and ceiling effects, but Twisk highlighted that this simply represents a linear transformation of change [11], and therefore does not deal with the problem of floor and ceiling effects. Twisk also describes the use of a longitudinal (multi-level) Tobit regression model to appropriately account for floor and ceiling effects in studies with repeated measures [12]. However, since its publication in 2009 there have only be a handful of analyses that use multi-level Tobit models[13-16], suggesting that lack of familiarity with these methods or understanding of when they can and should be applied has deterred analysts in their use, or when they can be applied.

Multi-level Tobit models are now incorporated in mainstream statistical software packages, such as Stata. Given their accesibility, they could arguably be used more frequently than they are. This is relavent considering that the use of measurement instruments with floor and ceiling effects are omnipresent in health related research. Examples include outcomes in health related quality of life (e.g. EQ5D, SF-36, SF-12), psychological wellbeing (e.g Hospital Anxiety and Depression Scale (HADS), Edinburgh Postnatal Depression Scale (EPDS)), and disease specific measures of wellbeing (e.g. Western Ontario and McMaster Universities Osteoarthritis Index (WOMAC) and Oxford Hip Score (OHS) as used in patients with osteoarthritis). Despite this, there is very little guidance available with respect to the consequences of using measurement instruments with floor or ceiling effects, when attempting to make inferences about the effect of an exposure on the change (between two time points) of an outcome of interest.

In this paper we use a Monte-Carlo simulation study to compare the performance of multi-level linear and Tobit models, Ordinarily Least Squares (OLS) regression and single-level Tobit regression, with and without adjustment for baseline scores, in the analysis of change in three different non-

For peer review only

randomised experiments, and a randomised experiment. We also demonstrate the use of these models using real world data from a large national joint replacement register.

We motivate the simulation and exemplar data analysis using an example from joint replacement research describing the association between body mass index (BMI) and the change in a disease specific patient reported outcome measure (PROM), the Oxford Hip Score (OHS). The issue is contentious in the UK [17-19] and USA [20] as some organisations suggest restricting joint-replacement to patients based on their BMI, citing an increased risk of revision surgery and lack of efficacy of surgery. The small increase in absolute risk of revision in obese patients, must be balanced against the other benefits of joint replacement, including a reduction in pain and improved physical functioning. Therefore, it is of interest to clinicians, policy makers, and patients to know the relative effect of obesity on the efficacy of total hip replacement compared to "normal weight patients".

For peer review only

Methods

Simulation Study Aims

We investigated the performance of four different methods of analysis, when estimating the effect of an exposure (BMI) on change in response (PROM) before and after total hip replacement with floor and ceiling effects using the Aims, Data Generating Process (DGP), Methods, Estimand, Performance (ADMEP) approach recomended by Morris et al. [21].

Data Generating Process (DGP)

We simulated longitudinal data of "well-being" before and after surgery. We assume that "well-being" is a latent, truly continuous and stable construct which is measured imperfectly by the OHS. Measurement error and floor/ceiling effects are then added to the latent construct to illustrate their consequences.

We assume the response, well-being, is a latent construct (y_{ij}^*) measured at the ith occasion, where i varies from 0 (pre-surgery) to 1 (1 year post-surgery), for the jth individual is modelled as a linear function of time. x_{0j} is mean-centred BMI categories according to WHO criteria i.e. -2 = BMI<18.5 (under weight), -1=18.5<BMI≤25 (normal), 0=25<BMI≤30 (overweight), 1=30<BMI≤35 (obese), and 2= BMI>35 (morbidly obese), i.e. x_{0j}=0 is a patient with a BMI classed as overweight.

$$y_{ij}^* = \beta_0 + u_{0j} + (\beta_1 + u_{1j})t_{ij} + \beta_2 x_{0j} + \beta_3 x_{0j} t_{ij}$$
$$\begin{bmatrix} u_{0j} \\ u_{1j} \end{bmatrix} \sim N(0, \Omega_u), \ \Omega_u = \begin{bmatrix} \sigma_{u0}^2 & \\ \sigma_{u01} & \sigma_{u1}^2 \end{bmatrix} \qquad 1$$

where t_{ij} is the time at which measurement i was taken on individual j, coded as 0 at pre-surgery and 1 post-surgery. $\beta_0$is the baseline population average response for a patient with average BMI, and u_{0j} represents the jth individual difference from the baseline response. The sum of $\beta_0 + u_{0j}$ is the individual baseline response for a patient with average BMI. β_1 represents the population average change per unit increase in time for a patient with average BMI, and u_{1j} represents the jth individual difference from the population average change per unit increase in time. The sum $\beta_1 + u_{1j}$ is the individual average change per unit increase in time for a patient with average BMI. β_2 represents the effect of a 1-unit increase in the exposure (x_{0j}) of interest (BMI) pre-surgery and β_3 represents the effect of a 1-unit increase in BMI (x_{0j}) on the pre-post surgery change in well-being (y_{ij}^*). The variance in individual deviations from the population average response at baseline and the average rate of change are σ_{u0}^2 and σ_{u1}^2 respectively. The covariance between baseline measurements and rate of change is characterised by σ_{u01} (with correlation ρ_{u01}).

For peer review only

Under the assumption of linear change, data was simulated from a multi-level model with a random intercept and slope, see Figure 1 for an illustration of a patient trajectory with an average BMI.

<Figure 1 Here>

The observed response without floor and ceiling effects (y_{ij}) is simulated by adding measurement error in the linear trajectory, ε_{ij} , to the latent response, where $\varepsilon_{ij} \sim N(0, \sigma_\varepsilon^2)$.

$$y_{ij} = y_{ij}^* + \varepsilon_{ij} \qquad 2$$

A response with floor and ceiling effects y_{ij}^{FC} is simulated by restricting the response to lie between 0 and 48.

$$y_{ij}^{FC} = \begin{cases} 0 & \text{if } y_{ij} \leq 0 \\ y_{ij} & \text{if } 0 < y_{ij} < 48 \\ 48 & \text{if } y_{ij} \geq 48 \end{cases} \qquad 3$$

See Figure 2 for a graphical illustration of the trajectory generation: we first simulate y_{ij}^*, then add some measurement error (ε_{ij}) to yield an observed response (y_{ij}), and finally add floor and ceiling effects to obtain the observed truncated response (y_{ij}^{FC}).

<Figure 2 Here>

We compared 4 DGPs to illustrate a range of scenarios by manipulating β_2, β_3, and $\rho_{u01}(\sigma_{u01})$ to influence the association between pre- and post-surgery outcomes. β_0, β_1, σ_{u0}, σ_{u1}, and $\sigma_{\varepsilon ij}$were fixed at 10, 40, 10, 15 and 3 respectively. **DGP 1** is a null model, where there is a baseline effect of the exposure is β_2= -3, but the exposure did not influence change over time (β_3= 0), and there is no correlation between baseline values and subsequent change (ρ_{u01}=0).

DGP 2 replicates a simple randomised trial where there is no difference between levels of the exposure at baseline (β_2= 0), but the exposure did influence change over time (β_3= -3), and there is no correlation between baseline values and subsequent change (ρ_{u01}=0). **DGP 3** and **DGP 4** replicate a cohort study, where there is a difference between levels of the exposure at baseline (β_2=-3), and the exposure also influenced change over time (β_3= -3). **DGP 3** specified no correlation between baseline values and subsequent change (ρ_{u01}=0), whereas **DGP 4** specified a negative correlation between baseline values and change (ρ_{u01}=-0.5), reflecting the fact the joint-replacement surgery has the tendency to normalise an individuals well-being, see Figure 3 for an illustration of the associated trajectories.

<Figure 3 Here>

We conducted a Monte-Carlo simulation with 1000 replicated datasets, each with 10,000 patients. A balanced dataset, i.e. 3 data points for each individual, was simulated to ensure identification of the

For peer review only

linear and Tobit multi-level models occurred, i.e. two data points allows estimation of baseline and change parameters but not measurement error. The middle data point was then dropped to replicate a pre/post design.

Method of analysis

For data sets with 3 measurement occasions, a linear multi-level model and a multi-level Tobit model (MLTM) that reflects the data generating process were fitted to the data, see equation 1.

In datasets with 2 measurement occasions, i.e. a pre-post design, single-level OLS and Tobit models were fitted to the data. Tobit models were only used when floor and ceiling effects had been simulated. Three different models were explored:

1) A simple model for post surgery well-being.

$$y_{1j}^{FC} = \alpha_1 + \alpha_2 x_{0j} + \varepsilon_j \qquad 4$$

2) A Simple Analysis of Change Score (SACS).

$$\left(y_{1j}^{FC} - y_{0j}^{FC}\right) = \alpha_6 + \alpha_7 x_{0j} + \varepsilon_j \qquad 5$$

3) A model for change adjusted for baseline i.e. baseline adjusted ANCOVA. This model is equivalent to a model for the post score adjusted for baseline ANCOVA, with the exception of the interpretation of the intercept.

$$\left(y_{1j}^{FC} - y_{0j}^{FC}\right) = \alpha_8 + \alpha_9 x_{0j} + \alpha_{10} y_{0j}^{FC} + \varepsilon_j \qquad 6$$

In addition, an under-identified MLTM model, equivalent to equation 1with constrained error variance σ_ε^2 was fitted in the spirit of a sensitivity analysis, where σ_ε^2 was constrained to a value from 5, 10, 15, 20, 25 and 30.

Estimand

The estimand of interest is the population average effect of the interaction between the exposure and change in slope i.e. β_3 the pre-post surgery change in well-being. We test whether the exposure modifies the improvement post-surgery (i.e. the null hypothesis that $\beta_3 = 0$).

Performance

The performance of each method was explored in terms of bias, coverage, empirical standard error, model based standard error, mean square error, relative error and relative precision.

National Joint Registry of England, Wales, Northern Ireland, and the Isle of Man (NJR)

For peer review only

Using data from the NJR, we investigated the association between BMI and a patient reported outcome measure, the OHS, in patients undergoing elective total hip replacement (THR) between 1st April 2003 and 22nd February 2017.

Data source

The NJR commenced data collection in April 2003; at inception it was mandatory for all THRs conducted in the private sector to be entered into the NJR, and from 2011 all THR procedures in the public and private sector were required to be entered into the NJR. A recent national audit of data entered into the NJR between 2014 and 2015 estimated data capture of 95% for primary THR and 91% for revision THR.

Inclusion/exclusion criteria

All consenting patients undergoing THR were eligible to be included in the analysis. Patients were included if their patient history was unique and consistent, i.e. contained no duplicates, revision prior to primary, or currently held in query by the submitting unit. Due to the requirement for reliable date information, patients who were indicated to have died prior to undergoing a procedure, were more than 110 years of age, had undergone a procedure prior to their date of birth, or received a procedure prior to 2003 were excluded from the analysis. Only primary THRs, where the primary indication for operation was osteoarthritis (OA) with unique prosthesis combinations were included in the analysis. All THRs with metal-on-metal bearing combinations were excluded from the analysis due to the exceptionally high failure rate in this group[22, 23]. Patients who were less than 50 years of age at the date of the index THR were also excluded, due to the high likelihood that these cases are due to OA secondary to other pathology.

See Supplementary.Figure 1 for a detailed breakdown of inclusion criteria.

Primary exposure

The primary exposure of interest in this study is BMI. BMI was introduced into the second "Minimal Data Set" in 2004. Patients with BMI between 10 and 60 were included in the analysis. BMI measures were excluded as implausible if height and weight measures were less than 130cm and weight less than 30kg respectively. See Supplementary.Figure 2.

Primary outcome

The primary outcome of interest in this study is change in OHS after surgery. Linked National PROMs were first available in 2009, see Supplementary.Figure 3 for details of linkage.

Confounding factors

Pre-operative confounding factors were thematically organised into groups: 1) Patient factors included sex, American Society of Anesthesiologists (ASA) grade, and operation funder. 2) Operation factors

For peer review only

included fixation, approach, patient position during surgery, anaesthetic type, thromboprophylaxis regime, bearing, and year of primary THR. 3) The setting of the treatment episode (i.e. private or NHS hospital). 4) Consultant based factors included the training status of the primary surgeon performing the operation. 5) Deprivation factors were based on the English indices of multiple deprivation (an area based index of deprivation).

Statistical analyses

Means, standard deviations and interquartile points were used to describe continuous variables. Frequencies and percentages were used to describe categorical variables.

The association between change in PROMS score was investigated using the same single-level methods and the ML Tobit model with constrained error variances described in the simulation study as an exemplar. In addition, we conducted more comprehensive analyses using restricted cubic splines to model the BMI association in the ML Tobit model with constrained error variance, single-level linear and Tobit SACS, ANCOVA, and Post score models. In the ML Tobit model, BMI was modelled with restricted cubic splines at baseline and its interaction with time. Correspondingly, we adjusted OHS for patient and deprivation confounding factors at baseline and operation, setting and confounding factors with an interaction with time i.e. operative factors and settings influence the change in outcome but not the baseline response. In single-level models, the effect of BMI was modelled using restricted cubic splines and adjusted for confounding factors using standard regression approaches.

Missing data

Due to the method of data collection in the national PROMS program, item non-response is masked. Defacto mean imputation of up to two missing items in the OHS occurred automatically. In addition, despite valid values appearing with individual OHS items, if the questionnaire was marked as “not complete”, implausible overall scores were obtained. For simplicity only patients with complete pre-operative and post-operative PROMS were used in the analysis. BMI is missing in a substantial proportion of the cohort. Patients prior to 2004 did not have BMI recorded, and the proportion of patients with missing BMI in 2004 is large. In 2009 ~40% of patients did not have BMI recorded; this reduced year on year and in 2016 was ~18% of eligible patients.

For pedagogic simplicity we use complete-case analyses throughout.

Patient and Public Involvement

Patient representatives sit on the committee structure of the National Joint Registry. The research priorities of the National Joint Registry are identified by this committee structure and approved by the patient representatives. Patients were not involved in the setting of the research question or the outcome measures, nor were they involved in designing or implementing this work or interpretation of

For peer review only

the results. We are unable to disseminate results of this study directly to study participants due to the anonymous nature of the data. We plan to disseminate our findings to the National Joint Registry, via their communications team, to relevant individuals with regards to the provision of joint replacement and to the general population through the local and national press.

Results

Simulation Study

Figure 4 illustrate the results from the MC simulation for each DGP. It is clear that MLM, OLS methods, and in DGP's 1, 3, and 4 (observational scenarios) single-level Tobit models all exhibit substantial bias. Only the ML Tobit with 3 datapoints provides unbiased estimates in all scenarios. Constrained ML Tobit models are close to being unbiased, but slightly over estimate the effect size, see Table 1, Single-level Tobit models also provide unbiased estimates for DGP 2 (the randomised trial). Empirical standard error, mean squared error, relative error and relative precision for each of the methods are reported in Supplementary.Table 1.

Figure 5 illustrates the spread of model based standard errors (SE) for each method by DGP. It is clear that the variation and absolute magnitude of SE in MLM with 3 data points per person is less than that of ML Tobit Models. Similarly, model based SEs from OLS methods are smaller and less variable than single-level Tobit methods. In DGP 2, the randomised trial, it is interesting to note that the SE from Tobit ANCOVA models are marginally smaller than for Tobit SACS. Whilst there is little difference in terms of bias from the constrained ML Tobit models, see Figure 4, the size and variability of estimated SEs increased with increasing value of the constrained of σ_{ε}^{2}.

Supplementary.Figure 4 to Supplementary.Figure 15, illustrate the coverage of 95% confidence intervals in each DGP. Unsurprisingly, coverage of methods which demonstrate bias is very poor, whilst coverage is at nominal levels for the ML Tobit model with three data points. The results from constrained ML Tobit indicate coverage less than the nominal levels. Coverage less than the advertised levels is principally due to the bias in estimate. However, when the estimates from the model are unbiased, as in DGP 2 with σ_{ε}^{2}=5, coverage is poor, suggesting bias in model based SE, i.e. they are too small.

National Joint Registry of England, Wales, Northern Ireland, and the Isle of Man.

Following application of inclusion and exclusion criteria, there were 162,513 patients with pre and post-operative OHS available for analysis. Figure 6 illustrates the results of the exemplar dataset using different approaches whilst attempting to estimate the effect of BMI category on the efficacy of surgery, whereas Figure 7 and Figure 8 illustrate the use of restricted cubic splines to assess the same question.

For peer review only

Exemplar Analysis

A single-level OLS SACS appoach suggests a positive association between BMI and change in OHS i.e. patients with greater BMIs have greater gains in well-being, whereas OLS ANCOVA and OLS post score models suggest a negative association. The single-level Post model score is approximately 50% greater than the ANCOVA model. All single-level Tobit models suggest a negative association between BMI and OHS. The Tobit SACS model is the smallest, with both the Tobit ANCOVA and Post models estimating substantially larger effects. The constrained ML Tobit models all provide equivalent (to 2 decimal places) results, suggesting there is no effect of BMI on the change in OHS pre and post surgery, see Figure 6.

Restricted Cubic Spline Approach

Crude analyses, which model BMI using restricted cubic splines, illustrate a complex association between BMI and pre-operative OHS. A ~4.5 point reduction in OHS is observed as BMI increases between 20 and 50 kg.m^{-2}. However, the change in OHS between pre- and post- surgery is very weakly associated with pre-operative BMI, with individuals with BMI's <25 kg.m^{-2} and >45 kg.m^{-2} receiving modestly greater gains than those patients with an average BMI of 28 kg.m^{-2}. However, with less than ½ a unit variation across the range of BMI observed in the cohort, the difference falls well below anything that could be considered clinically meaningful, see Figure 7. Following adjustment for patient factors, operation factors, centre factors, consultant factors, and deprivation there was little difference in the pattern of change compared to crude results, see Figure 7. Single-level approaches are illustrated in Figure 8, with OLS and Tobit models giving similar patterns of results. ANCOVA and the post model specification suggest a strong inverse association with BMI, with obese individuals receiving l ess improvement following surgery. OLS SACS indicate that obesity is associated with greater gains in OHS following surgery. Conversely, Tobit SACS models indicate that obesity is associated with smaller gains in OHS following surgery.

For peer review only

Discussion

The results of the simulation study clearly illustrate that, in the presence of floor and ceiling effects, neither baseline adjustment, or simple analysis of change scores (SACS) will yield unbiased estimates of the effect of an exposure on the outcome of interest. Single-level modifications to account for floor and ceiling effects such as the Tobit model only work in the context of a randomised trial, i.e. when there is no difference between baseline values by BMI. Importantly, single-level methods, OLS and Tobit models, induce significant bias, with negligible coverage, when $\beta_3 = 0$ i.e. there is no change in the pre- post- surgergy well being by BMI. Fully identified MLTM with three measurement occasions, return unbiased estimates with coverage close to advertised levels. In pre- post- designs

with two measurement occasions ML Tobit models, with constrained level 1 variances, return estimates very close to being unbiased, but coverage is less than advertised indicating bias in the model based standard errors.

The simulation study is consistent with a lay intuition with respect to analyses of floor and ceiling effects. Assuming we accept that either the MLM and OLS change analyses are appropriate in the absence of floor and ceiling effects, DGP 1 illustrates that when there is no effect of obesity on the efficacy of surgery, the addition of an artificial ceiling compresses the gain of individuals towards the top of the distribution. Due to the baseline association between obesity and well-being, underweight individuals tend to have gains that are more compressed compared to obese individuals. This inevitably induces bias, and provides evidence of a change in pre- post- surgery wellbeing by BMI, where none actually exist. Similarly, in DGP 2 (no baseline differences) where there is truly an interaction effect, will also lead to biased estimates. The DGP used in the simulation assumes underweight individuals benefit more from surgery than heavier individuals, which results in a fanning out of the trajectories. Underweight individuals have truly greater gains than obese individuals, but these gains are underestimated due to the ceiling effect, resulting in bias towards the null. In DGPs 3 and 4 (baseline differences in BMI, and interaction between BMI and change) we see a more extreme pattern of results compared to DGP 2, but overall consistency with the expected response of compressing individual gains which have initially higher starting values.

In the exemplar analysis of NJR data, the pattern of results is very similar to that of DGP 1 of the simulation, suggesting that results of the simulation are likely to be replicated in real world datasets. The more comprehensive analysis of the NJR data, using RCS to reflect the continuous nature of BMI, aptly illustrate where the effects from mis-specified single-level models are arising from. The ML Tobit model illustrates a strong negative association between BMI and pre-operative OHS, and failing to account for these baseline differences appropriately when attempting to estimate change leads to variation at baseline being incorporated in the estimate of change. Furthermore, the ability to adjust both baseline and post-surgical OHS for their pronounced floor and ceiling effects respectively, leads to unbiased estimates of the effect of interest. Unfortunately, due to the constraints on the level 1 variance, interpretation of the random effects are difficult, as they depend on the magnitude of the variance applied in the constraint, see Supplementary.Figure 16. However, the models clearly illustrate that change in PROMS following THR do not depend on BMI, and surgery appears to be effective for patients regardless of their BMI.

Conclusion

Floors and ceilings in PROM instruments have somewhat predictable effects on estimated coefficients from standard OLS models that do not adjust for floor or ceiling effects, assuming the true underlying association is known. As this is rarely the case, it is important to consider a variety of different data

For peer review only

generating processes to explore the likely impact on an analysis. It is important to consider the validity of the assumptions underpinning the Tobit model, i.e. that the latent response is truly continuous and that there is a true ceiling just beyond the range of the measurement being used.

Single-level Tobit models do not ameliorate floor and ceiling effects in simple analysis of change scores. However, ML Tobit models appear to recover the effects of interest under specific assumptions. The analysis of pre- post- designs require further constraints to ensure models are fully identified. The difference between analytical approaches can profoundly alter the interepretation of the model parameters, and this may have serious consequences if used to generate policy inappropriately. For example, inappropriate analyses that fail to consider data generating process appropriately may lead to the restriction of joint replacement for overweight or obese patients.

When designing a study to investigate the effect of an exposure on change in health status, it would be preferable to use a measurement instrument that does not have floor or ceiling effects as inference is less complicated, and design trumps analysis in most scenarios. If the use of measurement instrument with floor and ceiling effects is unavoidable, it is preferable to collect data at 3 time points which ensure models are fully identified, alleviating the need to constrain level 1 variance in order to identify models, again design trumps analysis. If retrospective analysis of pre-post data sets are required, it appears that using ML Tobit model with constrained level 1 error variance would be preferable to single-level approaches.

Broadly speaking the analyses of this simulation are in agreement with the work of Glymour et al., that analysis of change and its interaction with an exposure at baseline, should not be adjusted for baseline measurements in observational data. The presence of floor and ceiling effects in data requires additional assumptions which makes things marginally more complex.

Data Access

Data may be obtained from a third party and are not publicly available. Access to the data can be made via research requests to the National Joint Registry of England, Wales, Northern Ireland and the Isle of Man. Full details can be found at http://www.njrcentre.org.uk/njrcentre/Research/Research-requests.

Role of the funding source

The Medical Research Council or National Institute of Health Research had no role in the design and conduct of the study; collection, management, analysis, and interpretation of the data; preparation, review, or approval of the manuscript; and decision to submit the manuscript for publication

For peer review only

Acknowledgements

We thank the patients and staff of all the hospitals who have contributed data to the National Joint Registry. We are grateful to the Healthcare Quality Improvement Partnership, the National Joint Registry Steering Committee, and staff at the National Joint Registry for facilitating this work. The views expressed represent those of the authors and do not necessarily reflect those of the National Joint Registry Steering Committee or Healthcare Quality Improvement Partnership, who do not vouch for how the information is presented.

Ethical approval

Ethics approval of pseudo anonymised analysis of NJR data is considered as secondary use of clinical registry data, under HRA guidance this does not require formal ethical approval. However, all research projects are internally approved by the NJR. The full NJR privacy notice can be found online (http://www.njrcentre.org.uk/njrcentre/About-the-NJR/Privacy-Notice-GDPR).

Author contributions

AS, MRW, AJ, AM, AWB, and YBS were responsible for the study design, AS conducted the data analysis. AS, MRW, AJ, AM, AWB, and YBS were responsible for interpreting the data. AS, MRW, AJ, AM, AWB, and YBS prepared and edited and approved the final manuscript.

For peer review only

Tables

Table 1:

Model	DGP 1: β_3 =0		DGP2 : β_3 =-3		DGP 3: β_3=-3		DGP 4 : β_3 =-3	
Estimate								
MLM	1.1	(0.0024)	-1.36	(0.0023)	-0.26	(0.0024)	-0.23	(0.0025)
ML Tobit	-0.0056	(0.0038)	-3.03	(0.0037)	-3.04	(0.0037)	-3.01	(0.0037)
ML Tobit σ_ϵ^2=5	-0.13	(0.0044)	-3.01	(0.0042)	-3.13	(0.0046)	-2.57	(0.0038)
ML Tobit σ_ϵ^2=10	-0.093	(0.0044)	-3.09	(0.0042)	-3.14	(0.0045)	-3.05	(0.0041)
ML Tobit σ_ϵ^2=15	-0.057	(0.0044)	-3.09	(0.0042)	-3.12	(0.0045)	-3.11	(0.0043)
ML Tobit σ_ϵ^2=20	-0.04	(0.0044)	-3.08	(0.0042)	-3.11	(0.0045)	-3.12	(0.0044)
ML Tobit σ_ϵ^2=25	-0.031	(0.0044)	-3.07	(0.0042)	-3.1	(0.0045)	-3.11	(0.0044)
ML Tobit σ_ϵ^2=30	-0.026	(0.0044)	-3.07	(0.0042)	-3.09	(0.0045)	-3.1	(0.0045)
OLS SACS	1.1	(0.0024)	-1.36	(0.0023)	-0.26	(0.0024)	-0.23	(0.0025)
OLS ANCOVA	-0.31	(0.0022)	-1.36	(0.002)	-1.69	(0.0021)	-2.43	(0.0019)
OLS Post	-1.36	(0.0023)	-1.36	(0.0022)	-2.72	(0.0023)	-2.69	(0.0018)
Tobit SACS	-0.72	(0.0044)	-3.04	(0.0041)	-3.78	(0.0044)	-4.83	(0.0048)
Tobit ANCOVA	-0.5	(0.0046)	-3.09	(0.0042)	-3.61	(0.0045)	-5.5	(0.0042)
Tobit Post	-3.07	(0.0049)	-3.06	(0.0047)	-6.13	(0.005)	-6.14	(0.004)
Coverage								
MLM	0	(0)	0	(0)	0	(0)	0	(0)
ML Tobit	94.8	(0.7)	95.3	(0.67)	93.9	(0.76)	95.4	(0.66)
ML Tobit σ_ϵ^2=5	67.5	(1.48)	86.7	(1.07)	71.6	(1.43)	4.6	(0.66)
ML Tobit σ_ϵ^2=10	83.5	(1.17)	85	(1.13)	77.4	(1.32)	92.2	(0.85)
ML Tobit σ_ϵ^2=15	91.1	(0.9)	87.7	(1.04)	85.7	(1.11)	86.8	(1.07)
ML Tobit σ_ϵ^2=20	92.7	(0.82)	90	(0.95)	88.2	(1.02)	87.1	(1.06)
ML Tobit σ_ϵ^2=25	93.4	(0.79)	91.6	(0.88)	89.5	(0.97)	88.2	(1.02)
ML Tobit σ_ϵ^2=30	93.6	(0.77)	91.9	(0.86)	91.1	(0.9)	88.9	(0.99)
OLS SACS	0	(0)	0	(0)	0	(0)	0	(0)
OLS ANCOVA	0.8	(0.28)	0	(0)	0	(0)	0	(0)
OLS Post	0	(0)	0	(0)	2.4	(0.48)	0	(0)
Tobit SACS	0.1	(0.1)	94.4	(0.73)	0	(0)	0	(0)
Tobit ANCOVA	7.2	(0.82)	89.7	(0.96)	1.2	(0.34)	0	(0)
Tobit Post	0	(0)	93.3	(0.79)	0	(0)	0	(0)
Model SE								
MLM	0.074	(2E-05)	0.074	(2E-05)	0.077	(2E-05)	0.078	(2E-05)
ML Tobit	0.12	(3E-05)	0.12	(3E-05)	0.12	(3E-05)	0.12	(3E-05)
ML Tobit σ_ϵ^2=5	0.1	(3E-05)	0.1	(3E-05)	0.11	(3E-05)	0.11	(4E-05)
ML Tobit σ_ϵ^2=10	0.12	(3E-05)	0.12	(3E-05)	0.13	(4E-05)	0.12	(3E-05)
ML Tobit σ_ϵ^2=15	0.13	(4E-05)	0.13	(4E-05)	0.14	(4E-05)	0.13	(3E-05)
ML Tobit σ_ϵ^2=20	0.13	(5E-05)	0.13	(5E-05)	0.14	(5E-05)	0.14	(4E-05)
ML Tobit σ_ϵ^2=25	0.14	(5E-05)	0.13	(5E-05)	0.14	(6E-05)	0.14	(5E-05)
ML Tobit σ_ϵ^2=30	0.14	(5E-05)	0.13	(5E-05)	0.15	(6E-05)	0.14	(5E-05)
OLS SACS	0.074	(2E-05)	0.074	(2E-05)	0.077	(2E-05)	0.078	(2E-05)
OLS ANCOVA	0.07	(2E-05)	0.065	(2E-05)	0.072	(2E-05)	0.059	(2E-05)
OLS Post	0.07	(2E-05)	0.07	(2E-05)	0.072	(2E-05)	0.055	(2E-05)
Tobit SACS	0.13	(5E-05)	0.13	(5E-05)	0.14	(6E-05)	0.15	(6E-05)
Tobit ANCOVA	0.14	(6E-05)	0.14	(6E-05)	0.15	(6E-05)	0.13	(6E-05)
Tobit Post	0.15	(7E-05)	0.15	(6E-05)	0.16	(7E-05)	0.13	(6E-05)

For peer review only

For peer review only

Figure Legends

Figure 1: Graphical illustration of a multi-level random intercept and slope model used to generate data for a individual with average BMI.

Figure 2: Graphical illustration of the data generating process of the latent, measured, and measured response with floor and ceiling effects. The Latent Response is y_{ij}^{*}, the measured response is y_{ij}, and the measured response with censoring is y_{ij}^{FC} .

Figure 3: Graphical Illustration of the four Data Generating Processes used to investigate the effect of floor and ceiling effects on analysis of pre-post surgery change with BMI as an exposure.Horizontal red lines at 0 and 48 indicate floor and ceilings of the measurement instrument.

Figure 4: Plot of 1000 estimates by each DGP, for each method of analysis. Within each method, the verical axis is the repition number of each simulated dataset. The white pipe symbol is the average of the estimates.

Figure 5: Plot of 1000 estimated Standard Errors by each DGP, for each method of analysis. Within each method, the verical axis is the repition number of each simulated dataset. The white pipe symbol is the average of the standard errors.

Figure 6: Estimate and 95% Confidence Intervals of constrained ML Tobit, Single-level OLS and Tobit: ANCOVA, SACS, and Post models.

Figure 7: Estimates and 95% confidence intervals of baseline and change in Oxford Hip Score (OHS) pre and post surgery and its association with Body Mass Index (BMI) adjusted for confounding.

Figure 8: Estimates and 95% confidence intervals of single-level approaches to the analysis of change in Oxford Hip Score (OHS) pre and post surgery and its association with Body Mass Index (BMI) adjusted for confounding.

For peer review only

References

1. Frison L, Pocock SJ. Repeated measures in clinical trials: analysis using mean summary statistics and its implications for design. *Stat Med* 1992;11(13):1685-704. [published Online First: 1992/09/30]
2. Goldstein H. Tutorial in biostatistics-longitudinal data analysis (repeated measures) in clinical trials. *Stat Med* 2000;19(13):1821. [published Online First: 2000/06/22]
3. Kaiser L. Adjusting for baseline: change or percentage change? *Stat Med* 1989;8(10):1183-90. [published Online First: 1989/10/01]
4. Matthews JN, Campbell MJ. Adjusting for baseline: change or percentage change. *Stat Med* 1992;11(12):1624-6. [published Online First: 1992/09/15]
5. Senn S. Repeated measures in clinical trials: analysis using mean summary statistics and its implications for design. *Stat Med* 1994;13(2):197-8. [published Online First: 1994/01/30]
6. Senn S. Change from baseline and analysis of covariance revisited. *Stat Med* 2006;25(24):4334-44. doi: 10.1002/sim.2682 [published Online First: 2006/08/22]

7. Senn S, Stevens L, Chaturvedi N. Repeated measures in clinical trials: simple strategies for analysis using summary measures. *Stat Med* 2000;19(6):861-77. [published Online First: 2000/03/29]
8. Vickers AJ. The use of percentage change from baseline as an outcome in a controlled trial is statistically inefficient: a simulation study. *BMC medical research methodology* 2001;1:6. [published Online First: 2001/07/19]
9. Vickers AJ, Altman DG. Statistics notes: Analysing controlled trials with baseline and follow up measurements. *BMJ* 2001;323(7321):1123-4. [published Online First: 2001/11/10]
10. Glymour MM, Weuve J, Berkman LF, et al. When is baseline adjustment useful in analyses of change? An example with education and cognitive change. *Am J Epidemiol* 2005;162(3):267-78. doi: 10.1093/aje/kwi187 [published Online First: 2005/07/01]
11. Twisk J. Applied longitudinal data analysis for epidemiology : a practical guide. Cambridge: Cambride University Press 2004:167-178.
12. Twisk J, Rijmen F. Longitudinal tobit regression: a new approach to analyze outcome variables with floor or ceiling effects. *J Clin Epidemiol* 2009;62(9):953-8. doi: 10.1016/j.jclinepi.2008.10.003 [published Online First: 2009/02/13]
13. Holla JFM, van Beers-Tas MH, van de Stadt LA, et al. Depressive mood and low social support are not associated with arthritis development in patients with seropositive arthralgia, although they predict increased musculoskeletal symptoms. *RMD Open* 2018;4(1):e000653. doi: 10.1136/rmdopen-2018-000653 [published Online First: 2018/07/19]
14. Moran LJ, Fraser LM, Sundernathan T, et al. The effect of an antenatal lifestyle intervention in overweight and obese women on circulating cardiometabolic and inflammatory biomarkers: secondary analyses from the LIMIT randomised trial. *BMC Med* 2017;15(1):32. doi: 10.1186/s12916-017-0790-z [published Online First: 2017/02/15]
15. Ravona-Springer R, Moshier E, Schmeidler J, et al. Changes in glycemic control are associated with changes in cognition in non-diabetic elderly. *J Alzheimers Dis* 2012;30(2):299-309. doi: 10.3233/JAD-2012-120106 [published Online First: 2012/03/20]
16. Zhu L, Gonzalez J. Modeling Floor Effects in Standardized Vocabulary Test Scores in a Sample of Low SES Hispanic Preschool Children under the Multilevel Structural Equation Modeling Framework. *Front Psychol* 2017;8:2146. doi: 10.3389/fpsyg.2017.02146 [published Online First: 2018/01/10]
17. Coombes R. Rationing of joint replacements raises fears of further cuts. *BMJ* 2005;331(7528):1290. doi: 10.1136/bmj.331.7528.1290 [published Online First: 2005/12/03]
18. Finer N. Rationing joint replacements: trust's decision seems to be based on prejudice or attributing blame. *BMJ* 2005;331(7530):1472. doi: 10.1136/bmj.331.7530.1472-a [published Online First: 2005/12/17]
19. McNicol MW. Rationing joint replacements: ...and is false economy resulting in overall damage. *BMJ* 2005;331(7530):1473. doi: 10.1136/bmj.331.7530.1473 [published Online First: 2005/12/17]
20. Workgroup of the American Association of H, Knee Surgeons Evidence Based C. Obesity and total joint arthroplasty: a literature based review. *J Arthroplasty* 2013;28(5):714-21. doi: 10.1016/j.arth.2013.02.011 [published Online First: 2013/03/23]

For peer review only

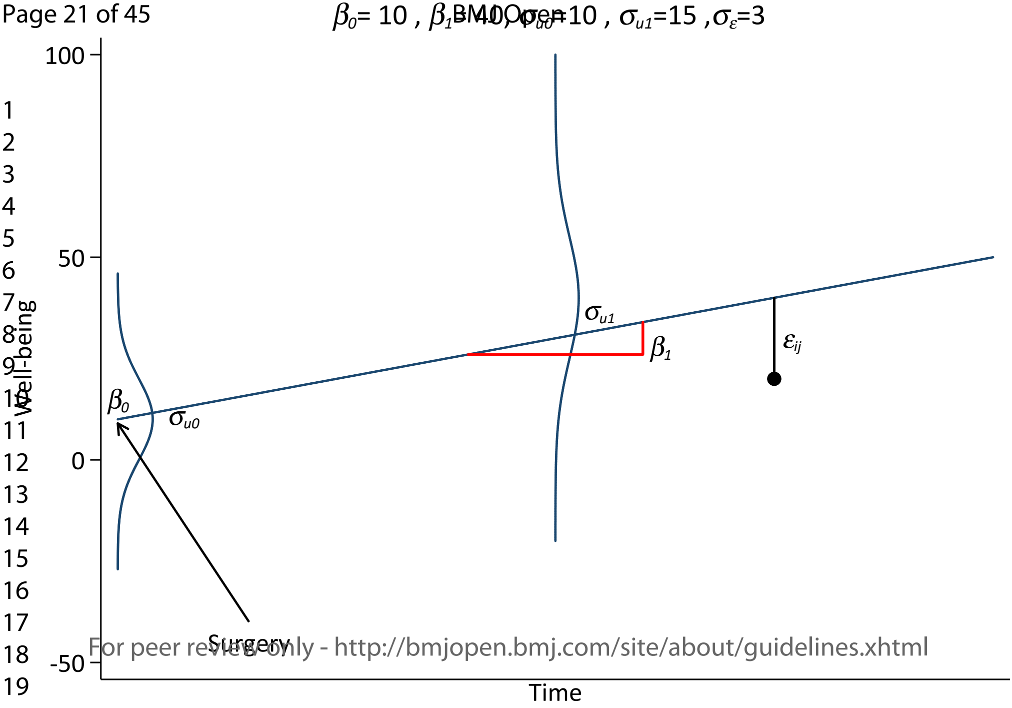

For peer review only - http://bmjopen.bmj.com/site/about/guidelines.xhtml

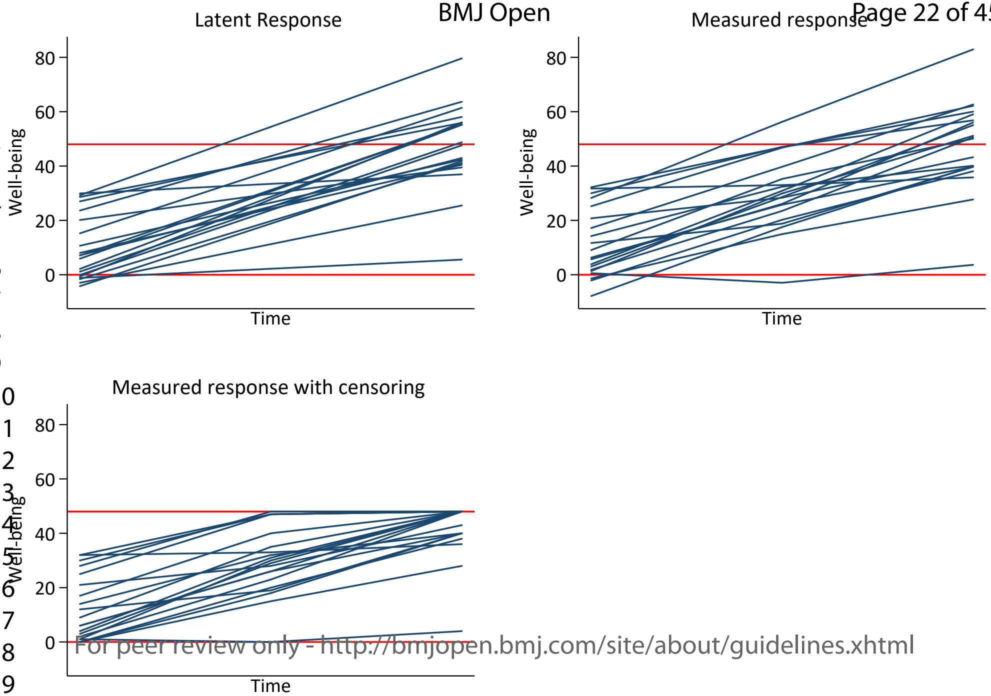

For peer review only - http://bmjopen.bmj.com/site/about/guidelines.xhtml

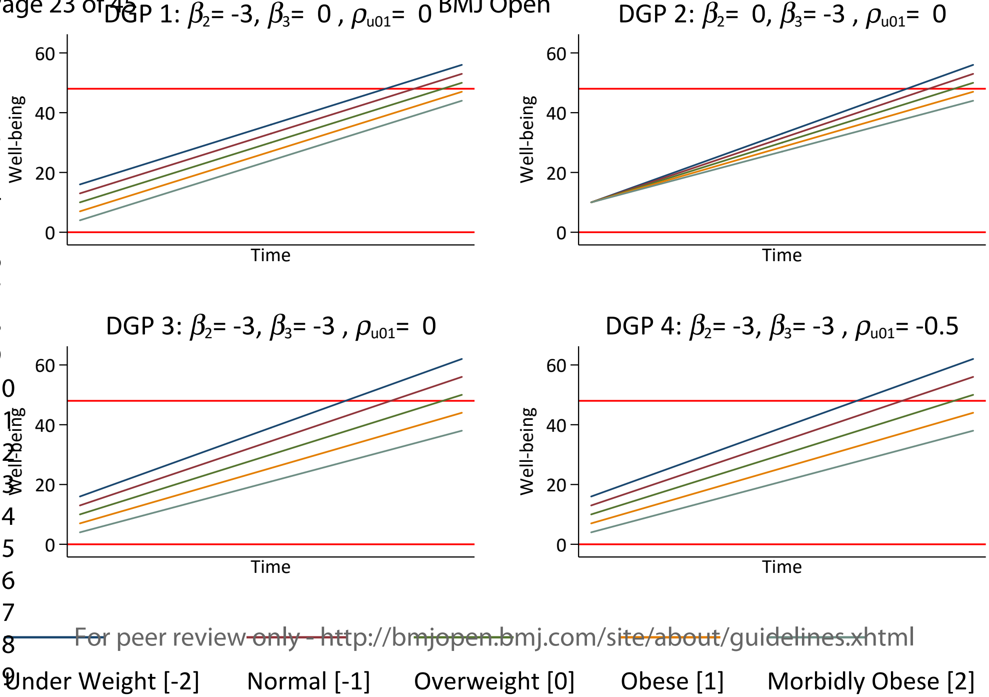
DGP 1: β_2= -3, β_3= 0 , ρ_{u01}= 0
DGP 2: β_2= 0, β_3= -3 , ρ_{u01}= 0
DGP 3: β_2= -3, β_3= -3 , ρ_{u01}= 0
DGP 4: β_2= -3, β_3= -3 , ρ_{u01}= -0.5
Well-being
0
20
40
60
Time
Under Weight [-2]
Normal [-1]
Overweight [0]
Obese [1]
Morbidly Obese [2]

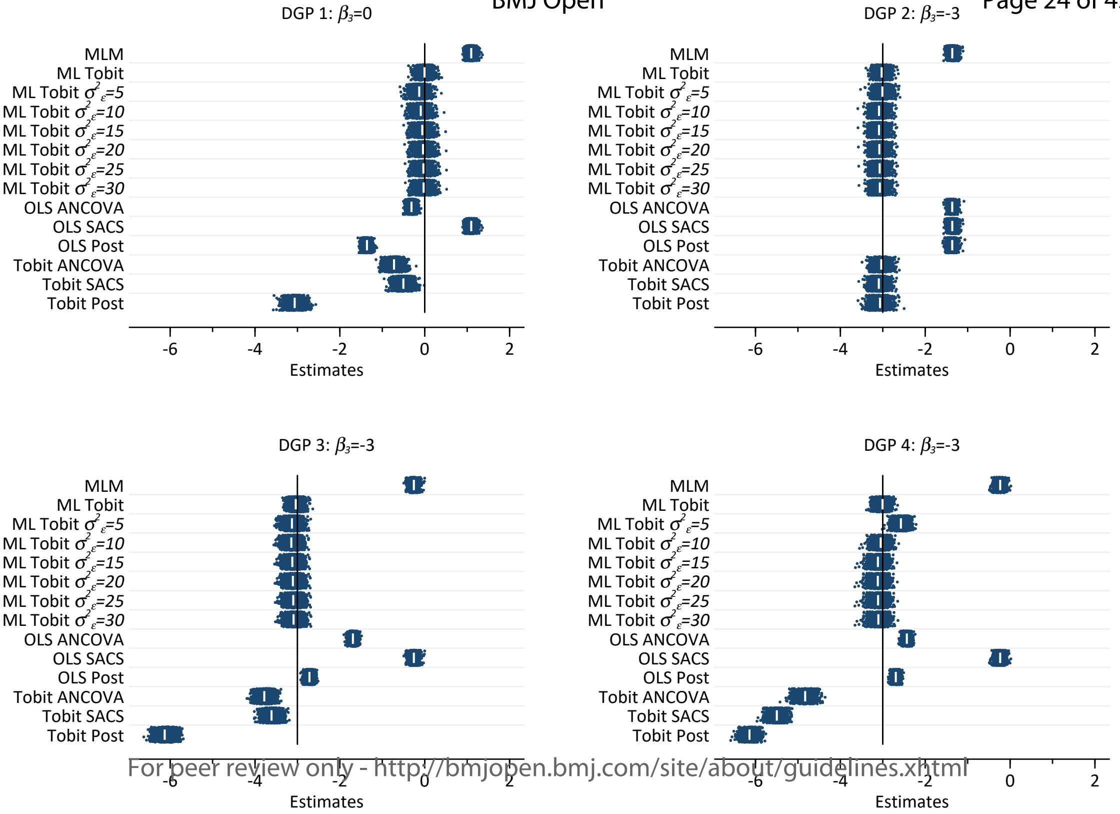

For peer review only - http://bmjopen.bmj.com/site/about/guidelines.xhtml

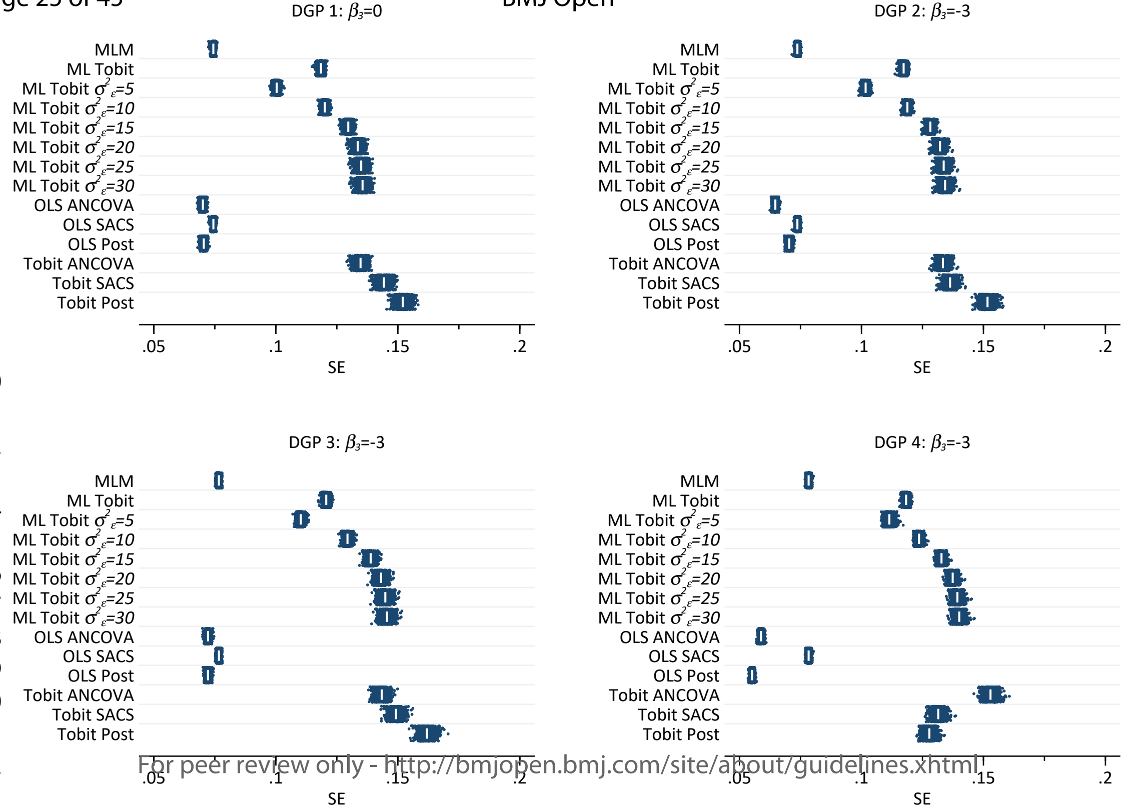
DGP 1: β_3=0
DGP 2: β_3=-3
DGP 3: β_3=-3
DGP 4: β_3=-3
MLM
ML Tobit
ML Tobit σ^2_ε=5
ML Tobit σ^2_ε=10
ML Tobit σ^2_ε=15
ML Tobit σ^2_ε=20
ML Tobit σ^2_ε=25
ML Tobit σ^2_ε=30
OLS ANCOVA
OLS SACS
OLS Post
Tobit ANCOVA
Tobit SACS
Tobit Post
.05
.1
.15
.2
SE

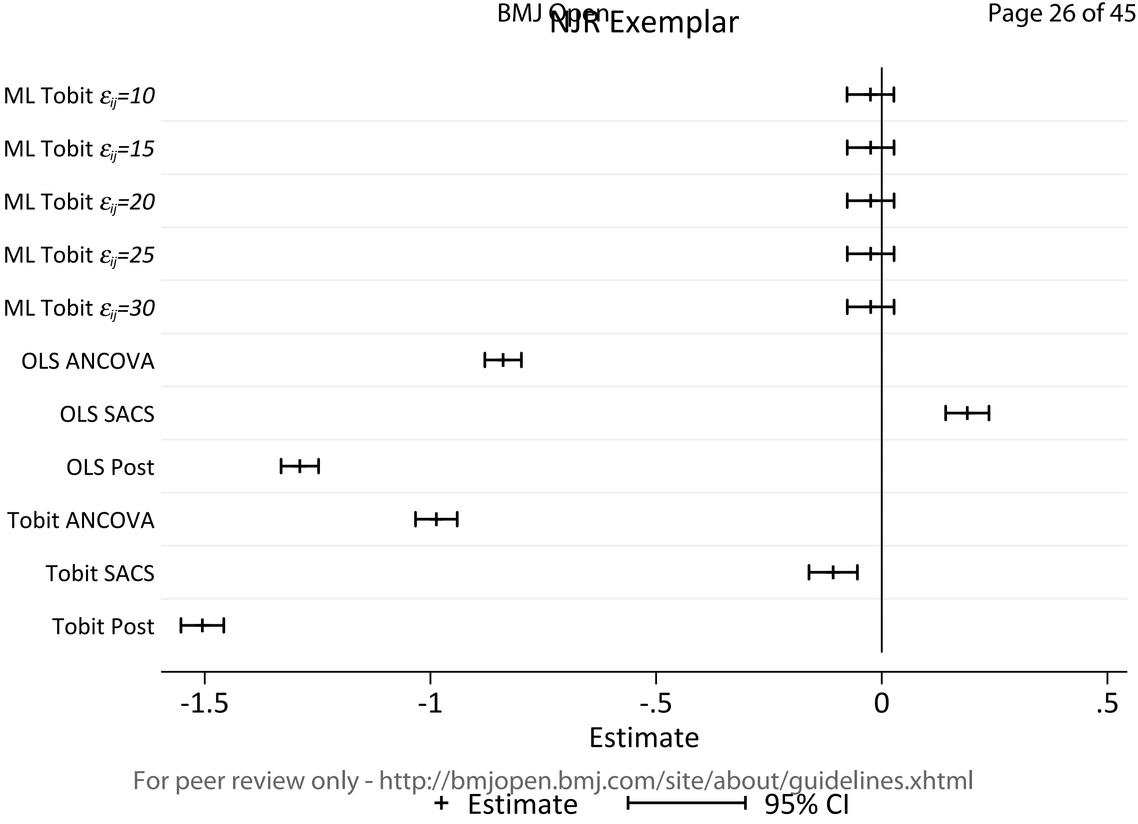

For peer review only - http://bmjopen.bmj.com/site/about/guidelines.xhtml

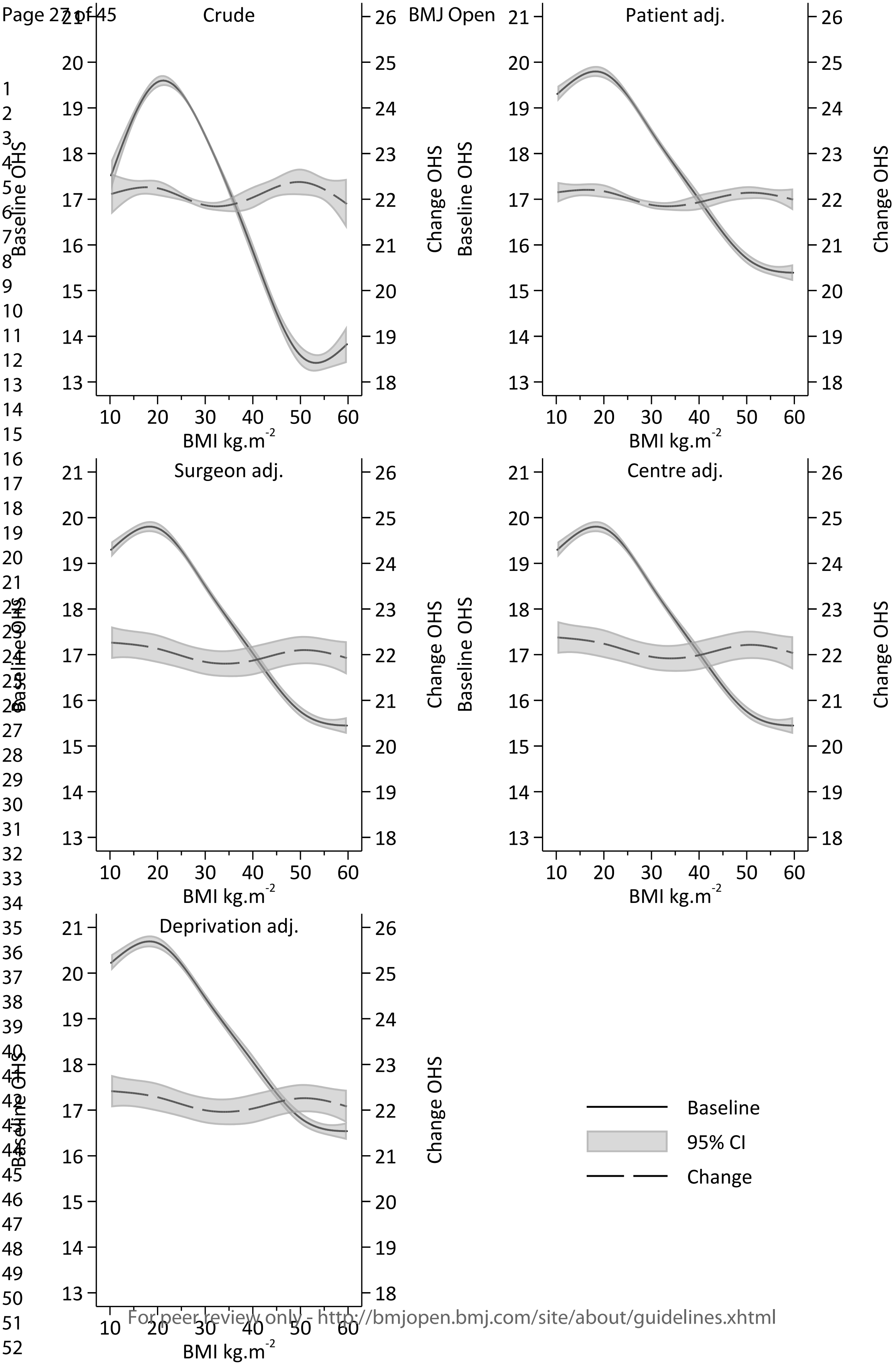
Crude
Patient adj.
Surgeon adj.
Centre adj.
Deprivation adj.
Baseline OHS
Change OHS
BMI kg.m-2
Baseline
95% CI
Change

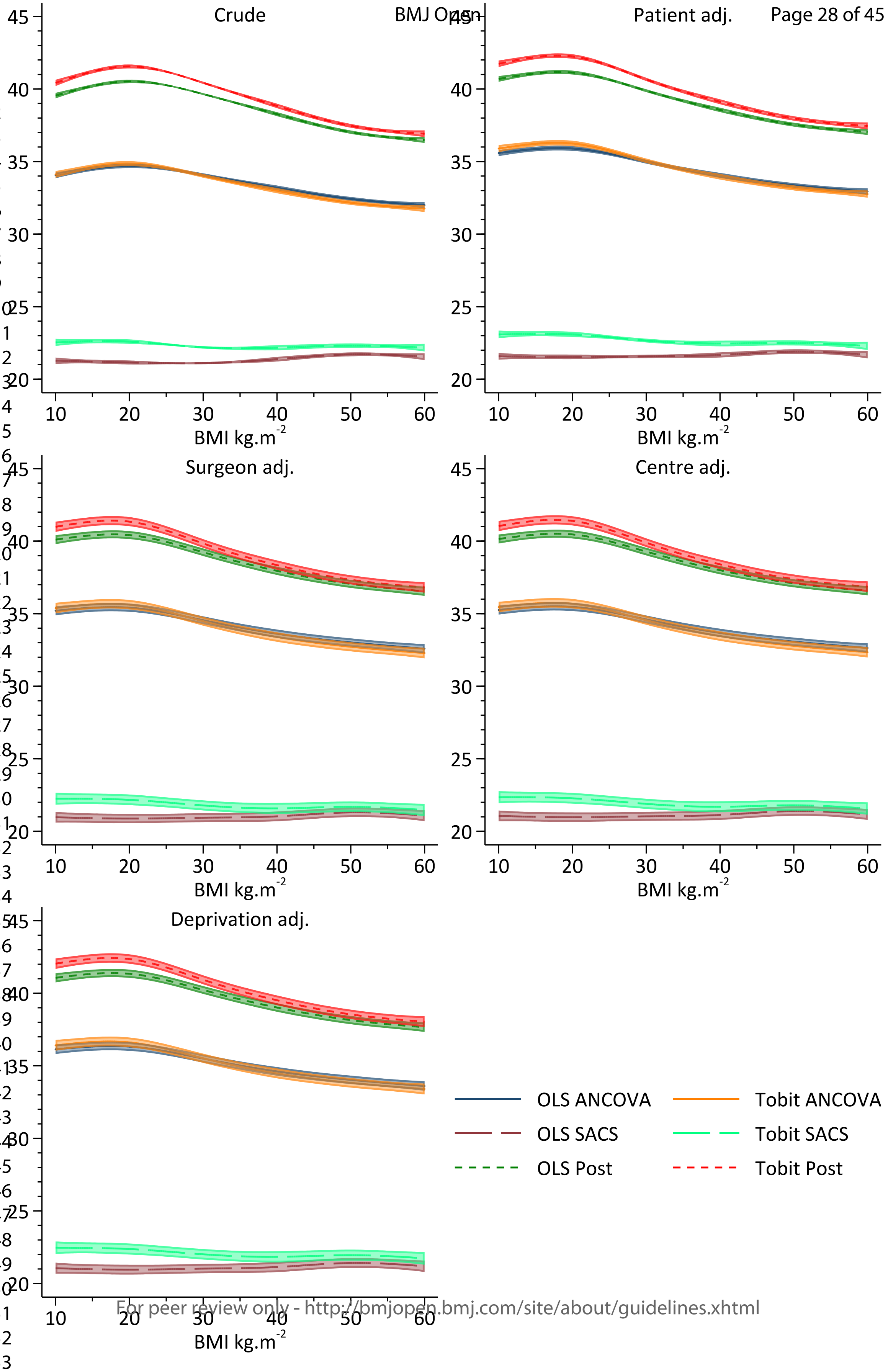
Crude
Patient adj.
Surgeon adj.
Centre adj.
Deprivation adj.
45
40
35
30
25
20
10
20
30
40
50
60
BMI kg.m^{-2}
OLS ANCOVA
OLS SACS
OLS Post
Tobit ANCOVA
Tobit SACS
Tobit Post

Supplementary Tables

Supplementary.Table 1: Simulation estimates of performance characteristics (Monte Carlo Standarc Error in parantheses)

	Model	DGP 1: β_3 =0		DGP2 : β_3 =-3		DGP 3: β_3 =-3		DGP 4 : β_3 =-3	
Emperical SE	MLM	0.075	(0.0017)	0.072	(0.0016)	0.075	(0.0017)	0.079	(0.0018)
	ML Tobit	0.12	(0.0027)	0.12	(0.0026)	0.12	(0.0026)	0.12	(0.0026)
	ML Tobit σ_ε^2=5	0.14	(0.0031)	0.13	(0.0029)	0.14	(0.0032)	0.12	(0.0027)
	ML Tobit σ_ε^2 =10	0.14	(0.0031)	0.13	(0.003)	0.14	(0.0032)	0.13	(0.0029)
	ML Tobit σ_ε^2=15	0.14	(0.0031)	0.13	(0.003)	0.14	(0.0032)	0.14	(0.003)
	ML Tobit σ_ε^2=20	0.14	(0.0031)	0.13	(0.003)	0.14	(0.0032)	0.14	(0.0031)
	ML Tobit σ_ε^2=25	0.14	(0.0031)	0.13	(0.003)	0.14	(0.0032)	0.14	(0.0031)
	ML Tobit σ_ε^2=30	0.14	(0.0031)	0.13	(0.003)	0.14	(0.0032)	0.14	(0.0031)
	OLS SACS	0.075	(0.0017)	0.072	(0.0016)	0.075	(0.0017)	0.079	(0.0018)
	OLS ANCOVA	0.07	(0.0016)	0.064	(0.0014)	0.068	(0.0015)	0.059	(0.0013)
	OLS Post	0.072	(0.0016)	0.07	(0.0016)	0.071	(0.0016)	0.056	(0.0013)
	Tobit SACS	0.14	(0.0031)	0.13	(0.0029)	0.14	(0.0031)	0.15	(0.0034)
	Tobit ANCOVA	0.14	(0.0032)	0.13	(0.003)	0.14	(0.0032)	0.13	(0.003)
	Tobit Post	0.16	(0.0035)	0.15	(0.0034)	0.16	(0.0035)	0.13	(0.0028)
Mean Square Error	MLM	1.21	(0.0052)	2.69	(0.0075)	7.54	(0.013)	7.66	(0.014)
	ML Tobit	0.014	(0.0007)	0.014	(0.0006)	0.015	(0.0007)	0.014	(0.0006)
	ML Tobit σ_ε^2=5	0.036	(0.0015)	0.017	(0.0008)	0.038	(0.0015)	0.2	(0.0033)
	ML Tobit σ_ε^2=10	0.028	(0.0012)	0.025	(0.0011)	0.041	(0.0015)	0.019	(0.0009)
	ML Tobit σ_ε^2=15	0.023	(0.001)	0.026	(0.0011)	0.035	(0.0013)	0.032	(0.0014)
	ML Tobit σ_ε^2=20	0.021	(0.0009)	0.024	(0.001)	0.032	(0.0013)	0.033	(0.0014)
	ML Tobit σ_ε^2=25	0.02	(0.0009)	0.023	(0.001)	0.03	(0.0012)	0.032	(0.0014)
	ML Tobit σ_ε^2=30	0.02	(0.0009)	0.022	(0.001)	0.029	(0.0012)	0.031	(0.0014)
	OLS SACS	1.21	(0.0052)	2.69	(0.0075)	7.54	(0.013)	7.66	(0.014)
	OLS ANCOVA	0.1	(0.0014)	2.68	(0.0066)	1.71	(0.0056)	0.33	(0.0021)
	OLS Post	1.86	(0.0062)	2.68	(0.0073)	0.085	(0.0013)	0.097	(0.0011)
	Tobit SACS	0.54	(0.0064)	0.018	(0.0008)	0.63	(0.0069)	3.37	(0.018)
	Tobit ANCOVA	0.27	(0.0046)	0.027	(0.0011)	0.39	(0.0056)	6.25	(0.021)
	Tobit Post	9.42	(0.03)	0.027	(0.0012)	9.81	(0.031)	9.88	(0.025)
Relative Error	MLM	-1.31	(2.21)	1.98	(2.28)	2.74	(2.3)	-0.31	(2.23)
	ML Tobit	-0.95	(2.22)	0.79	(2.25)	2.37	(2.29)	1.66	(2.27)
	ML Tobit σ_ε^2=5	-28.5	(1.6)	-22.8	(1.73)	-23.8	(1.71)	-7.01	(2.08)
	ML Tobit σ_ε^2=10	-14.5	(1.91)	-10.3	(2.01)	-8.8	(2.04)	-3.97	(2.15)
	ML Tobit σ_ε^2=15	-7.35	(2.07)	-3.54	(2.16)	-2.41	(2.18)	-2.46	(2.18)
	ML Tobit σ_ε^2=20	-4.29	(2.14)	-0.57	(2.22)	0.26	(2.24)	-1.4	(2.21)
	ML Tobit σ_ε^2=25	-3.04	(2.17)	0.65	(2.25)	1.35	(2.27)	-0.8	(2.22)
	ML Tobit σ_ε^2=30	-2.51	(2.18)	1.17	(2.26)	1.83	(2.28)	-0.47	(2.23)
	OLS SACS	-1.3	(2.21)	1.99	(2.28)	2.75	(2.3)	-0.3	(2.23)
	OLS ANCOVA	-0.5	(2.23)	1.51	(2.27)	6.84	(2.39)	-0.54	(2.23)
	OLS Post	-1.96	(2.19)	-0.086	(2.24)	1.46	(2.27)	-1.25	(2.21)

	Tobit SACS	-2.97	(2.17)	2.09	(2.28)	2.82	(2.3)	-0.056	(2.24)
	Tobit ANCOVA	-0.19	(2.23)	2.21	(2.29)	4.01	(2.33)	-1.35	(2.21)
	Tobit Post	-2.82	(2.17)	1.27	(2.27)	2.74	(2.3)	0.83	(2.26)
Relative Precision	MLM	247	(14.1)	232.3	(13.9)	275.4	(16.9)	132.5	(6.66)
	ML Tobit	37.8	(4.36)	28.3	(4.07)	50.5	(5.64)	6.08	(3.13)
	ML Tobit σ_ε^2=5								
	ML Tobit σ_ε^2=10	-0.034	(0.97)	-0.92	(1.07)	3.91	(1.24)	-13.3	(1.5)
	ML Tobit σ_ε^2=15	0.62	(1.22)	-1.66	(1.37)	3.24	(1.52)	-22.5	(1.68)
	ML Tobit σ_ε^2=20	1.22	(1.3)	-1.7	(1.44)	2.57	(1.6)	-26	(1.7)
	ML Tobit σ_ε^2=25	1.67	(1.33)	-1.52	(1.47)	2.31	(1.62)	-27.2	(1.7)
	ML Tobit σ_ε^2=30	1.99	(1.34)	-1.34	(1.47)	2.29	(1.63)	-27.5	(1.69)
	OLS SACS	247	(14.1)	232.3	(13.9)	275.4	(16.9)	132.5	(6.66)
	OLS ANCOVA	297.2	(16)	327.4	(14.8)	357.6	(20.9)	310.3	(18.8)
	OLS Post	281.7	(15.5)	250	(14.8)	312.9	(19.2)	360.1	(21.2)
	Tobit SACS	2.26	(1.94)	1.8	(1.89)	7.48	(2.72)	-38.6	(2.12)
	Tobit ANCOVA	-5.67	(3.07)	-2.33	(1.55)	1.52	(3.56)	-19.1	(3.59)
	Tobit Post	-19.5	(2.6)	-22.5	(2.6)	-15.9	(2.95)	-10.6	(4.08)

For peer review only

Supplementary Figures

Supplementary.Figure 1: Inclusion / Exclusion Criteria of the NJR study.

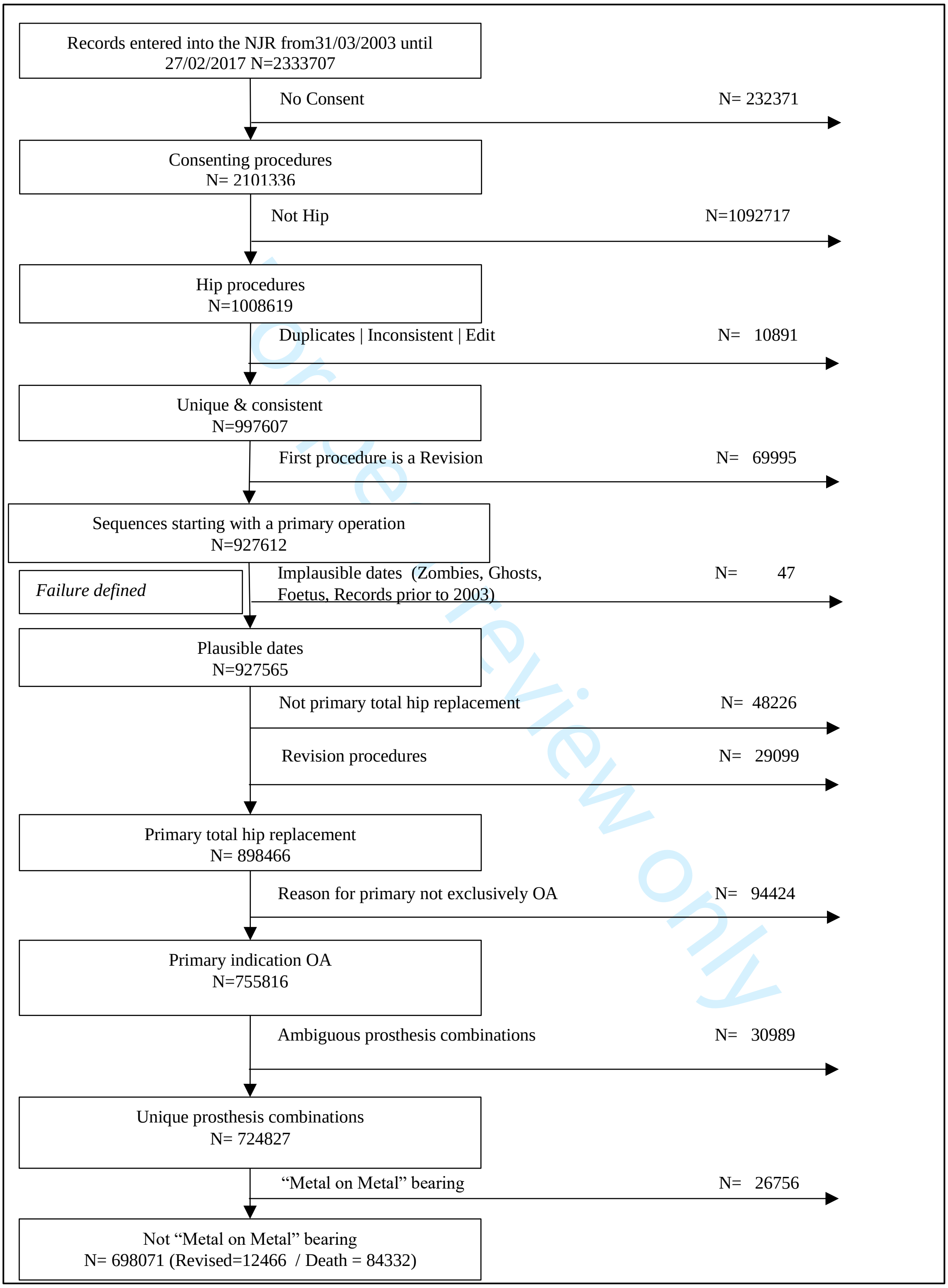

Supplementary.Figure 2: Description of covariate missing data in eligible data

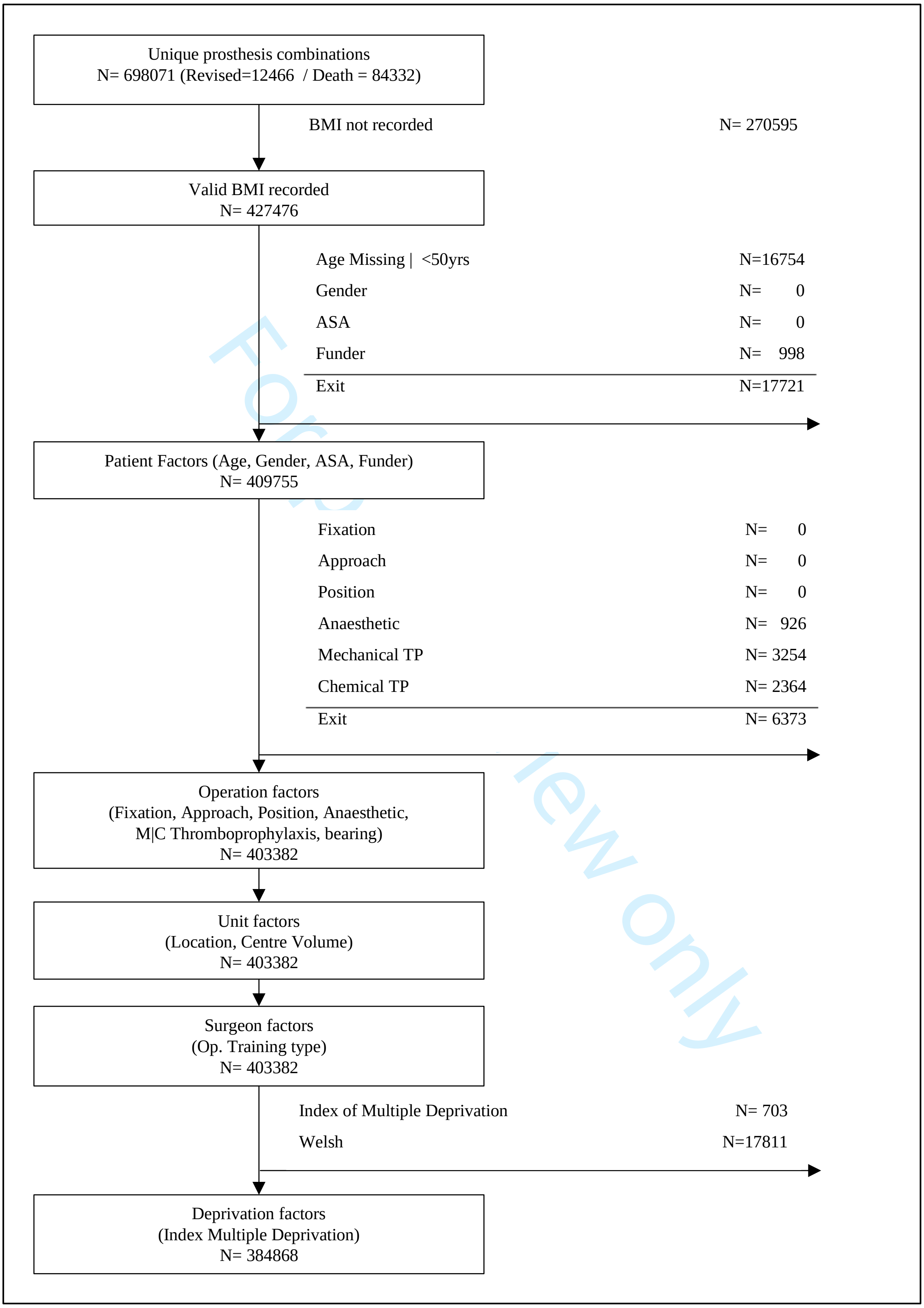

Supplementary.Figure 3: Description of National PROMS linkage to the NJR

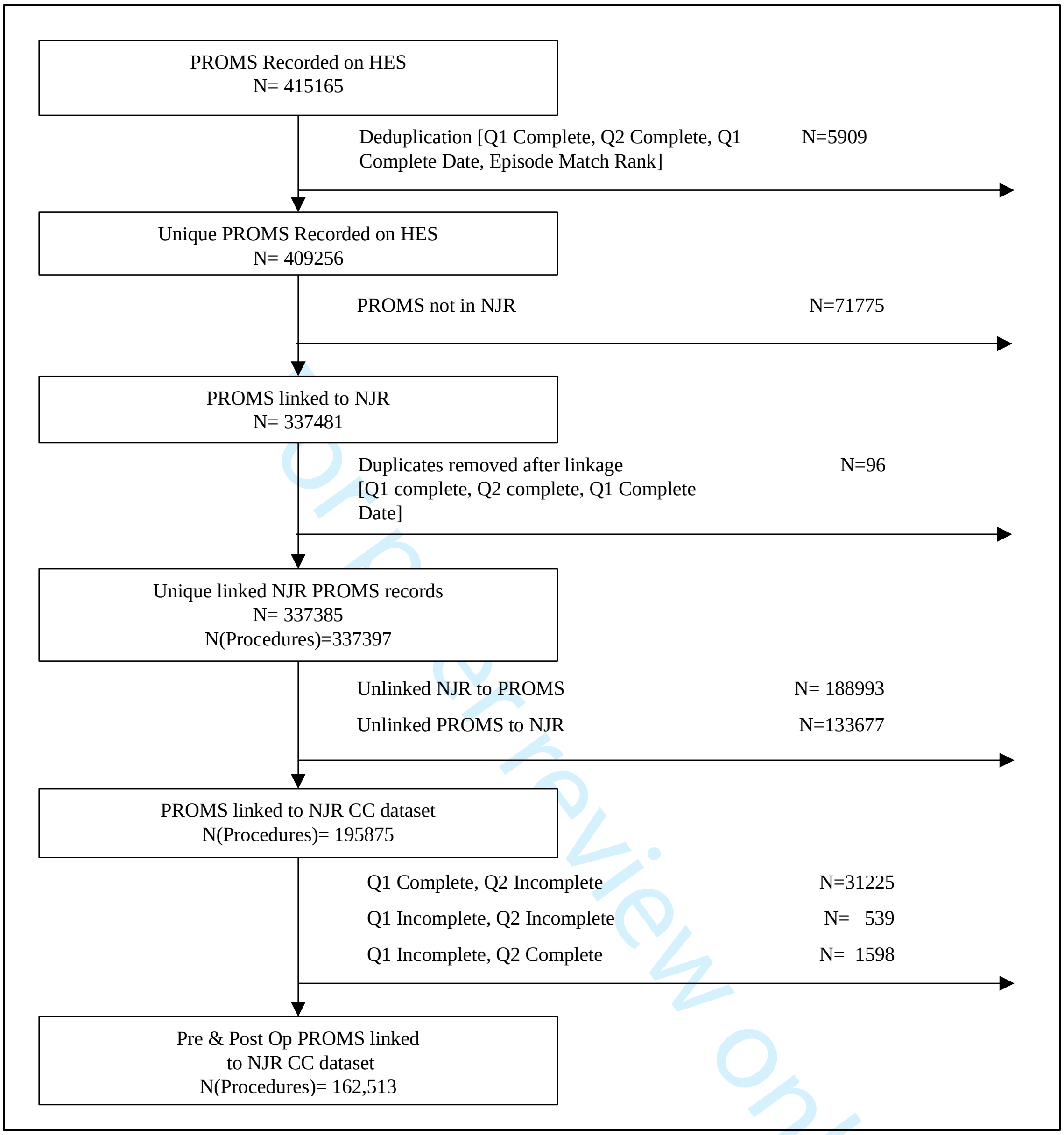

For peer review only

Supplementary.Figure 4: "Zip Plot" of the 1000 95% Confidence intervals for each method of analysis for DGP 1. The vertical axis is the centile of the two-sided p-value against H_0 : β_3=0 associated with the confidence interval for MLM and ML Tobit models.

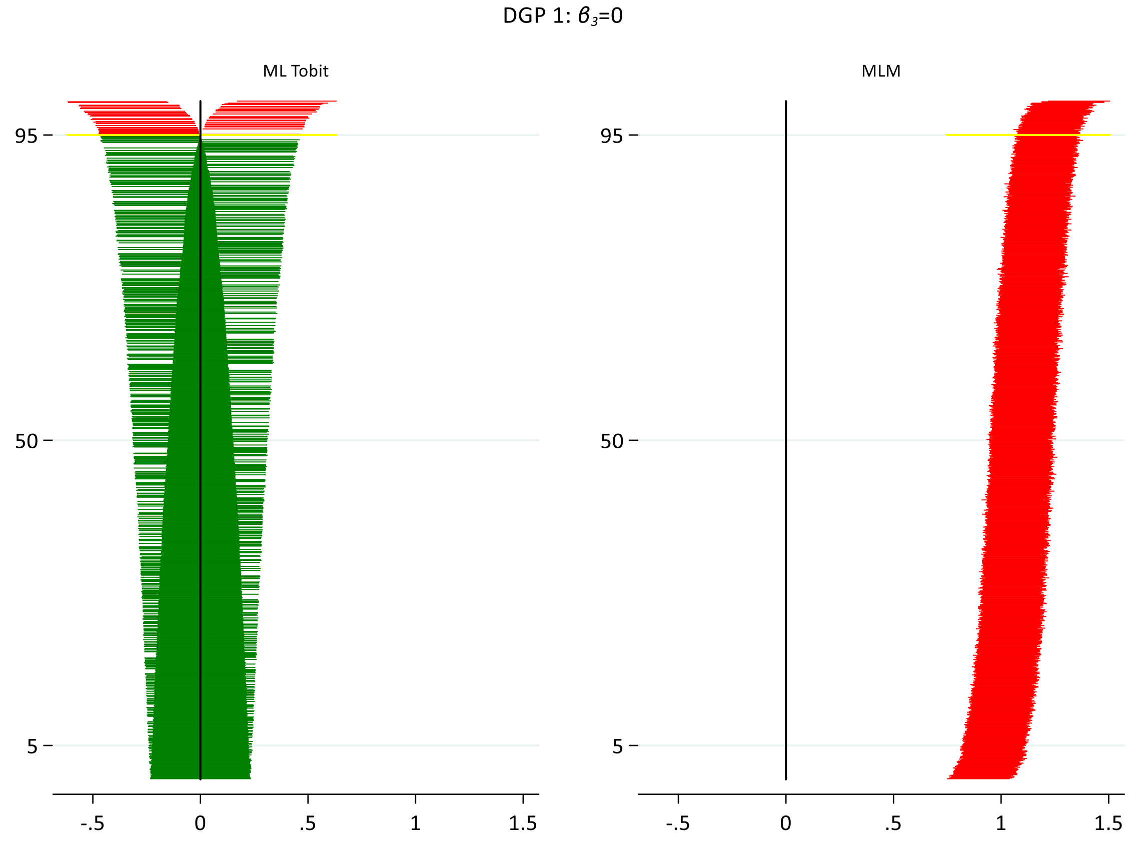

For peer review only - http://bmjopen.bmj.com/site/about/guidelines.xhtml

Supplementary.Figure 5: "Zip Plot" of the 1000 95% Confidence intervals for each method of analysis for DGP 1. The vertical axis is the centile of the two-sided p-value against H_0 : β_3=0 associated with the confidence interval for ML Tobit models with varying constraints of σ_ε^2

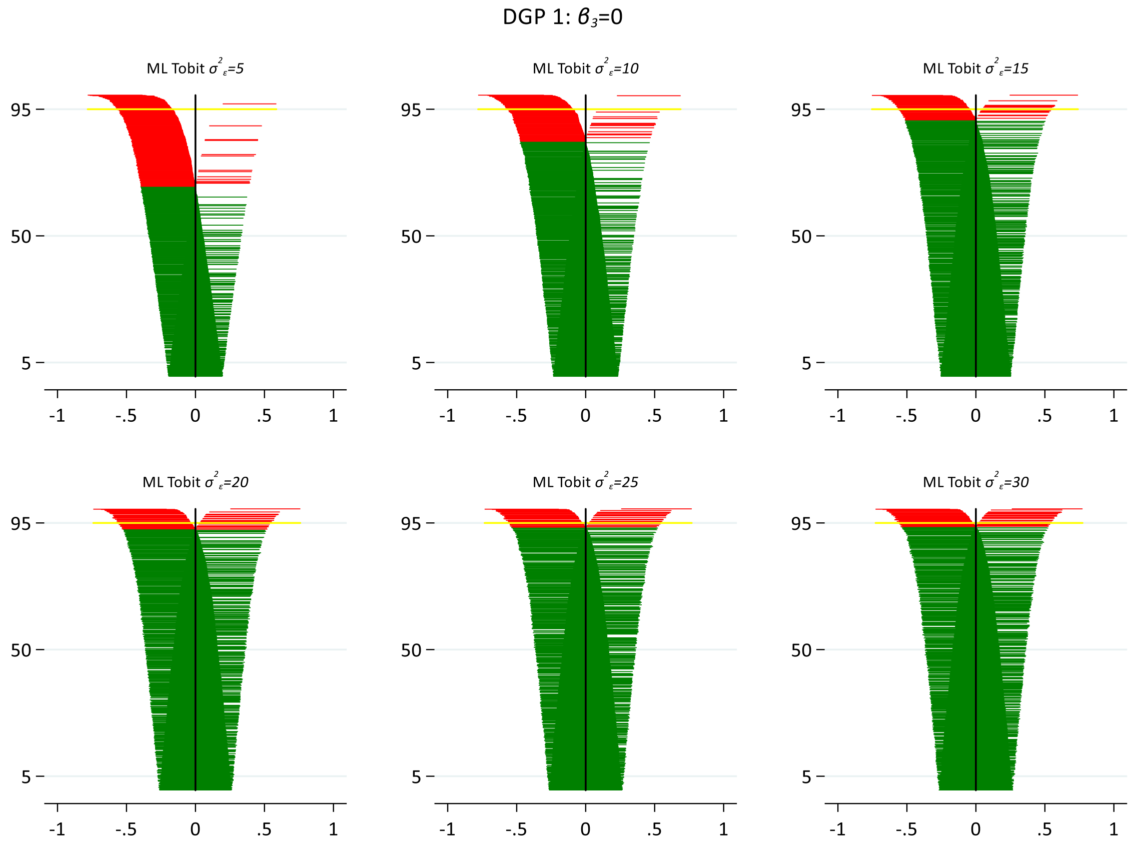

w only

Supplementary.Figure 6: "Zip Plot" of the 1000 95% Confidence intervals for each method of analysis for DGP 1. The vertical axis is the centile of the two-sided p-value against H_0 : β_3=0 associated with the confidence interval for Single level OLS and Tobit models.

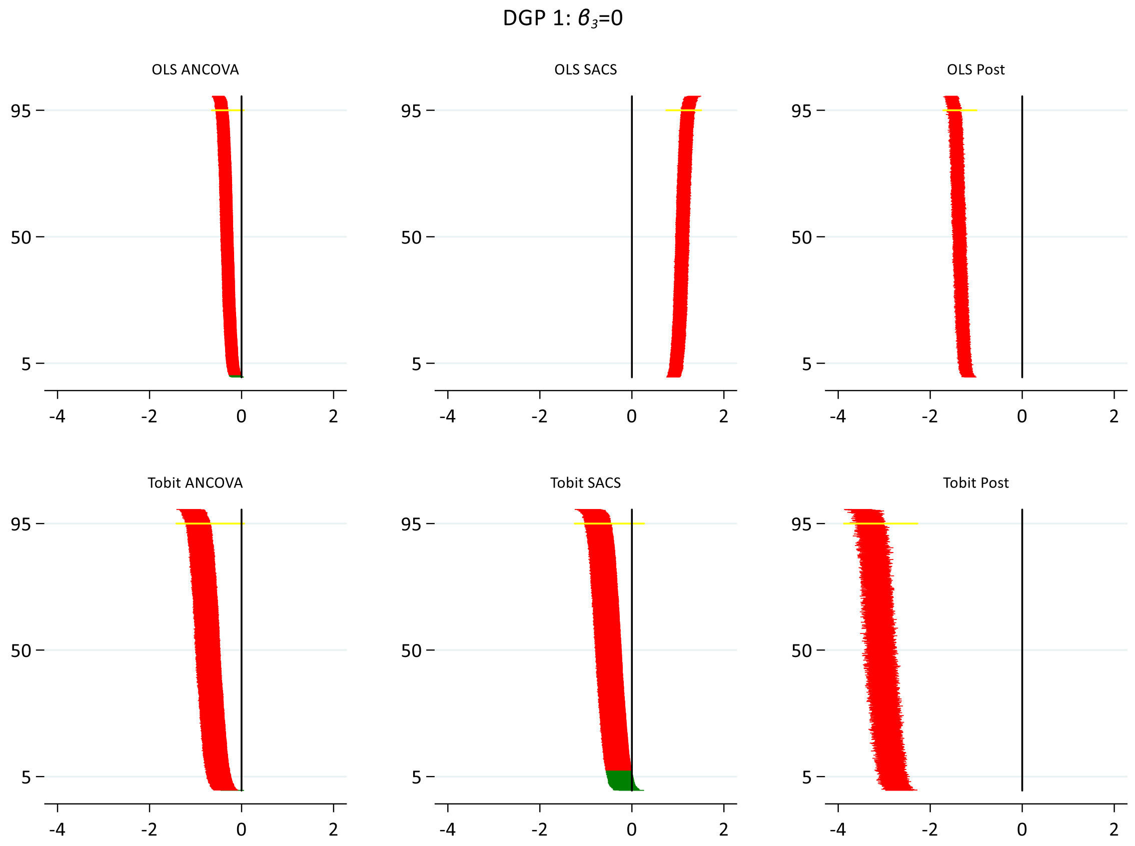

For peer review only - http://bmjopen.bmj.com/site/about/guidelines.xhtml

Supplementary.Figure 7: "Zip Plot" of the 1000 95% Confidence intervals for each method of analysis for DGP 2. The vertical axis is the centile of the two-sided p-value against H_0 : β_3=3 associated with the confidence interval for MLM and ML Tobit models.

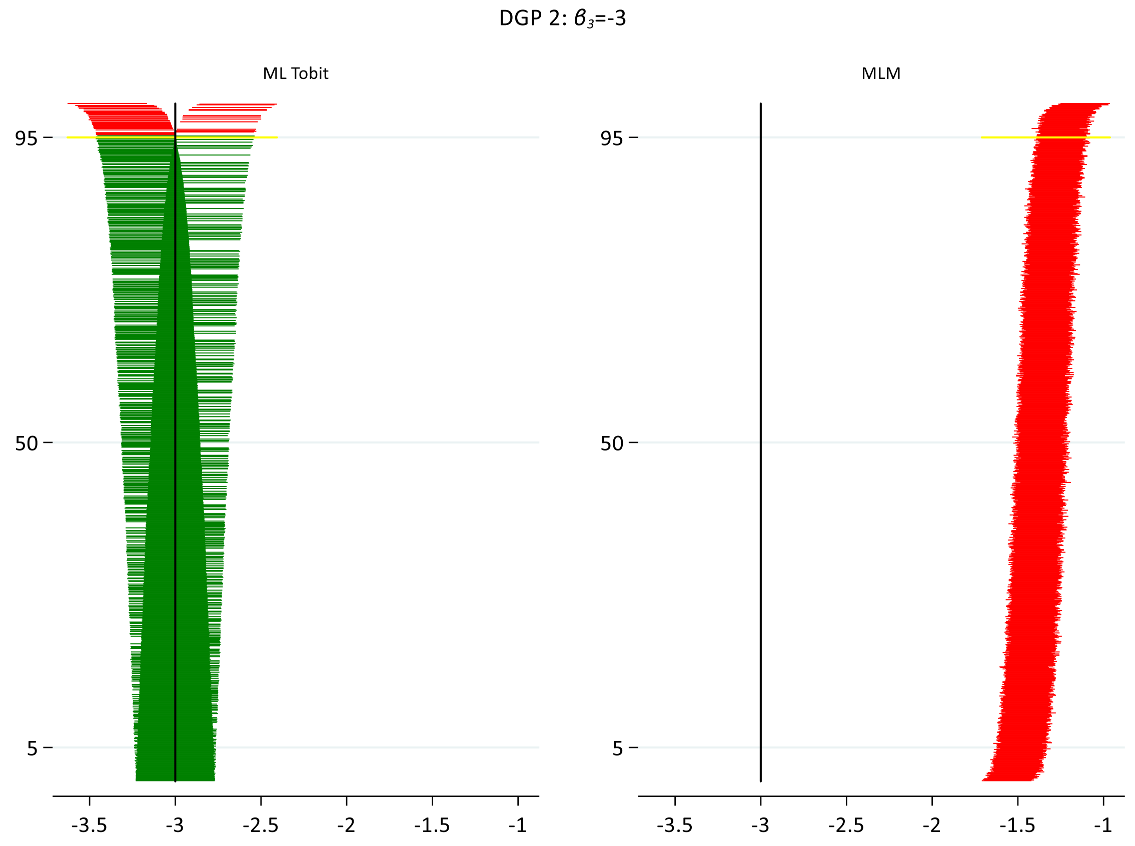

ew only

Supplementary.Figure 8: "Zip Plot" of the 1000 95% Confidence intervals for each method of analysis for DGP 2. The vertical axis is the centile of the two-sided p-value against H_0 : β_3=3 associated with the confidence interval for ML Tobit models with varying constraints of σ_ε^2

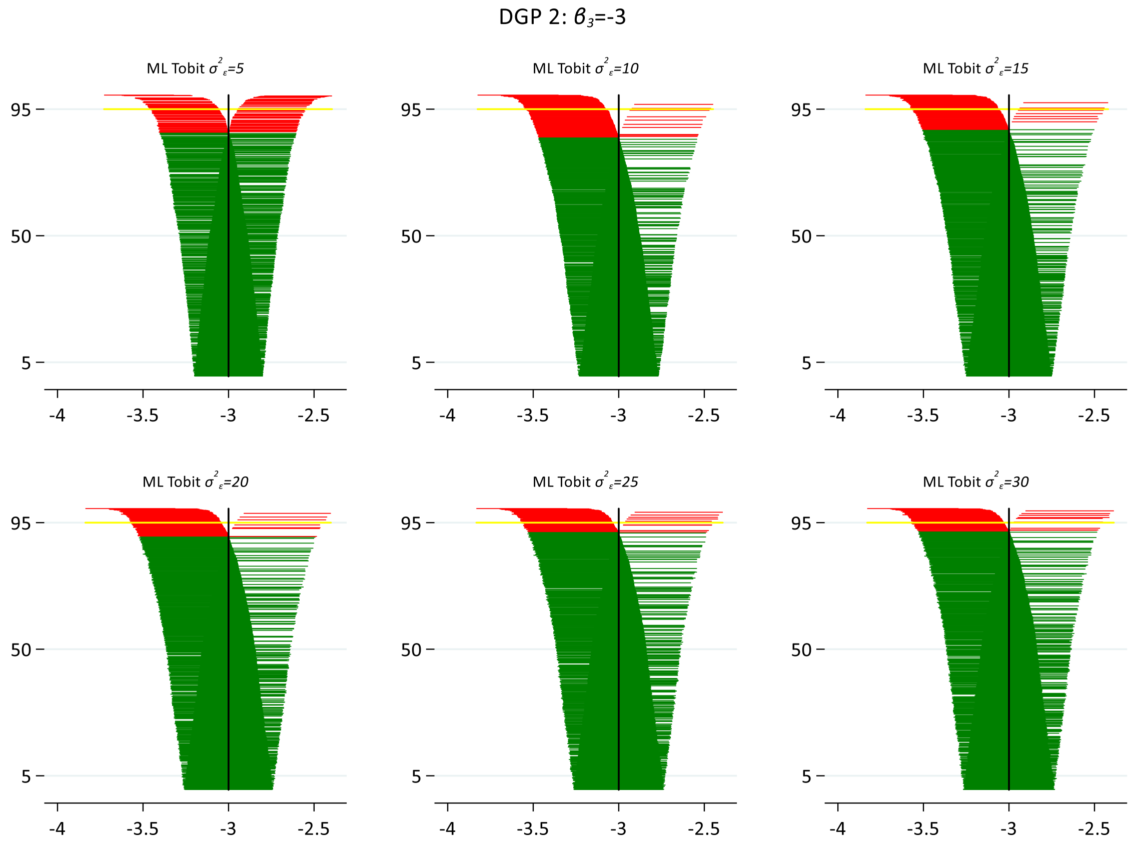

w only

Supplementary.Figure 9: "Zip Plot" of the 1000 95% Confidence intervals for each method of analysis for DGP 2. The vertical axis is the centile of the two-sided p-value against H_0 : β_3=3 associated with the confidence interval for Single level OLS and Tobit models.

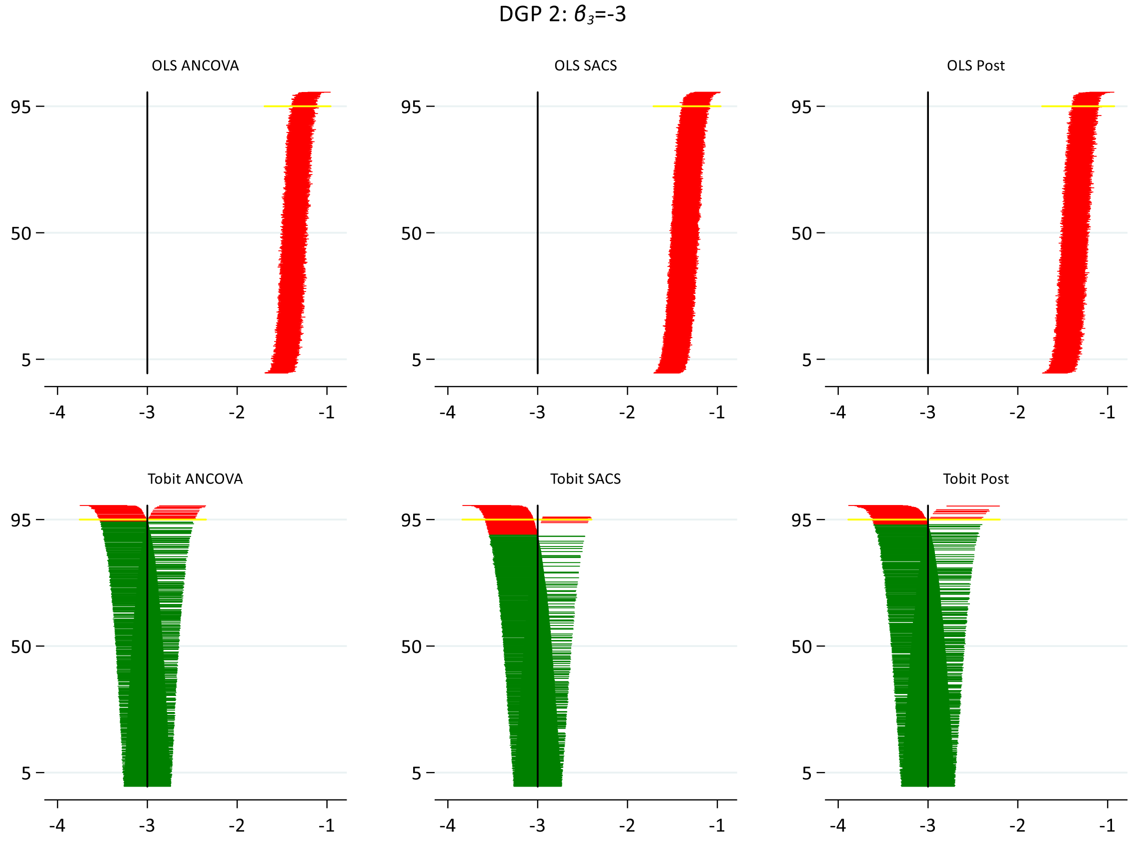

ew only

Supplementary.Figure 10: "Zip Plot" of the 1000 95% Confidence intervals for each method of analysis for DGP 3. The vertical axis is the centile of the two-sided p-value against H_0 : β_3=3 associated with the confidence interval for MLM and ML Tobit models.

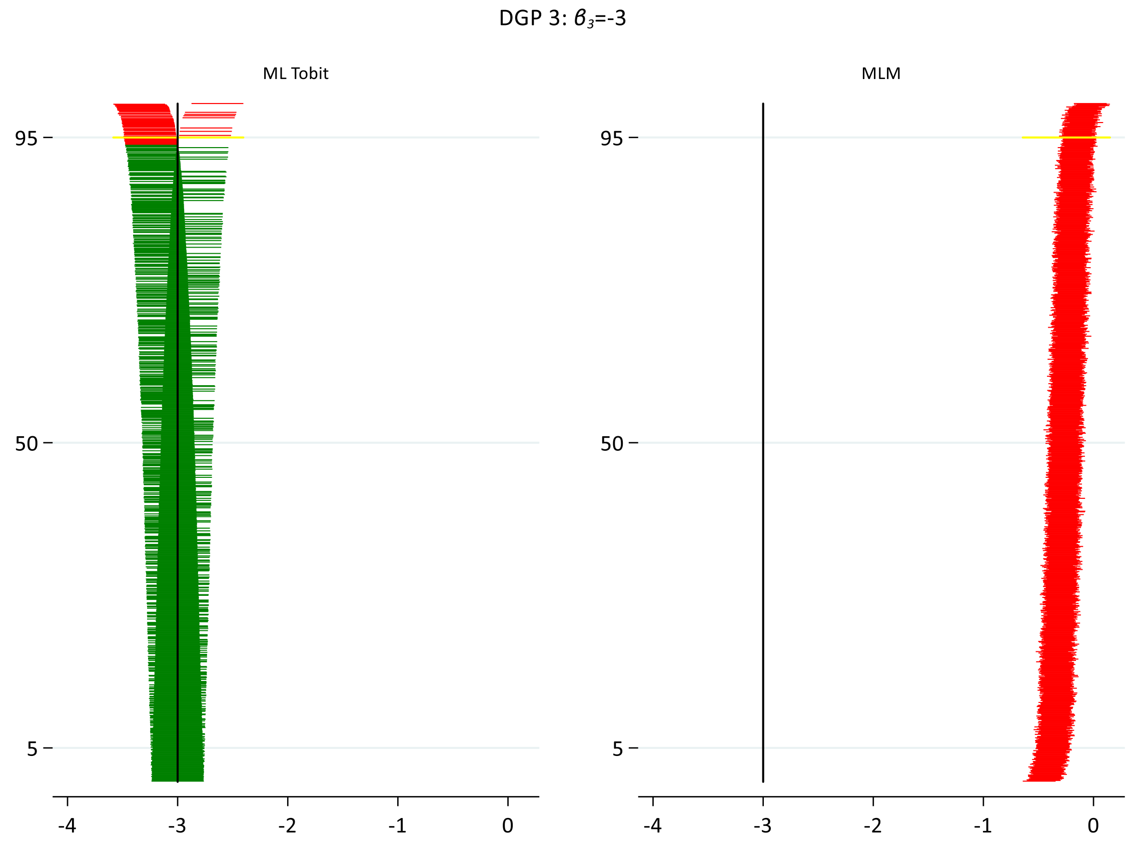

For peer review only - http://bmjopen.bmj.com/site/about/guidelines.xhtml

Supplementary.Figure 11: "Zip Plot" of the 1000 95% Confidence intervals for each method of analysis for DGP 3. The vertical axis is the centile of the two-sided p-value against H_0 : β_3=3 associated with the confidence interval for ML Tobit models with varying constraints of σ_ε^2

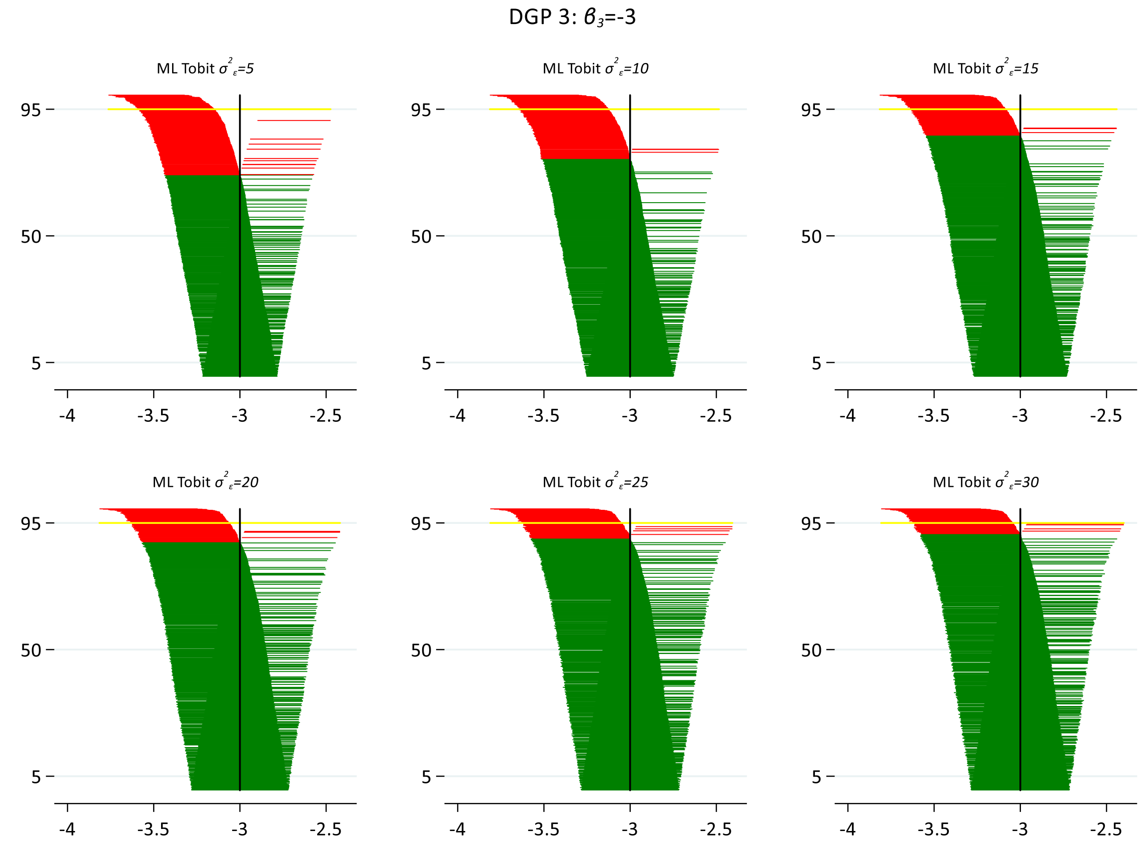

w only

Supplementary.Figure 12: "Zip Plot" of the 1000 95% Confidence intervals for each method of analysis for DGP 3. The vertical axis is the centile of the two-sided p-value against H_0 : β_3=3 associated with the confidence interval for Single level OLS and Tobit models.

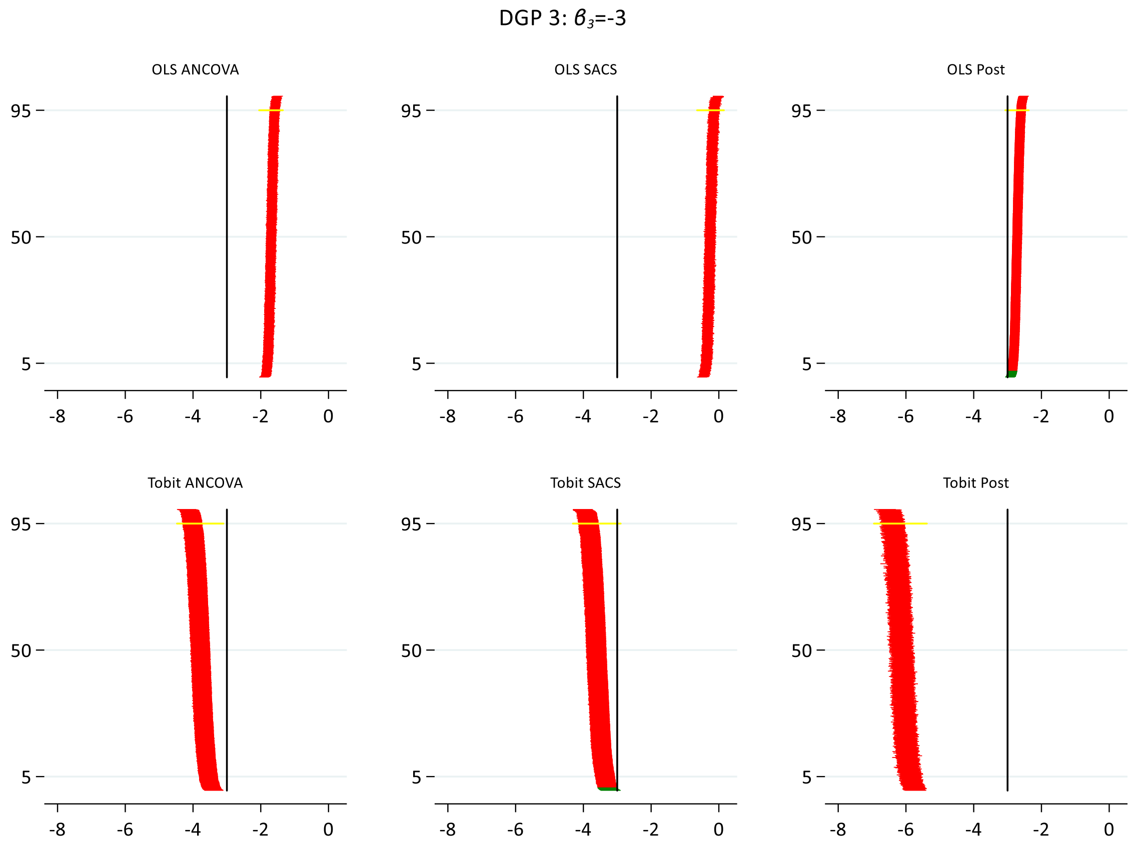

For peer review only - http://bmjopen.bmj.com/site/about/guidelines.xhtml

Supplementary.Figure 13: "Zip Plot" of the 1000 95% Confidence intervals for each method of analysis for DGP 4. The vertical axis is the centile of the two-sided p-value against H_0 : β_3=0 associated with the confidence interval for MLM and ML Tobit models.

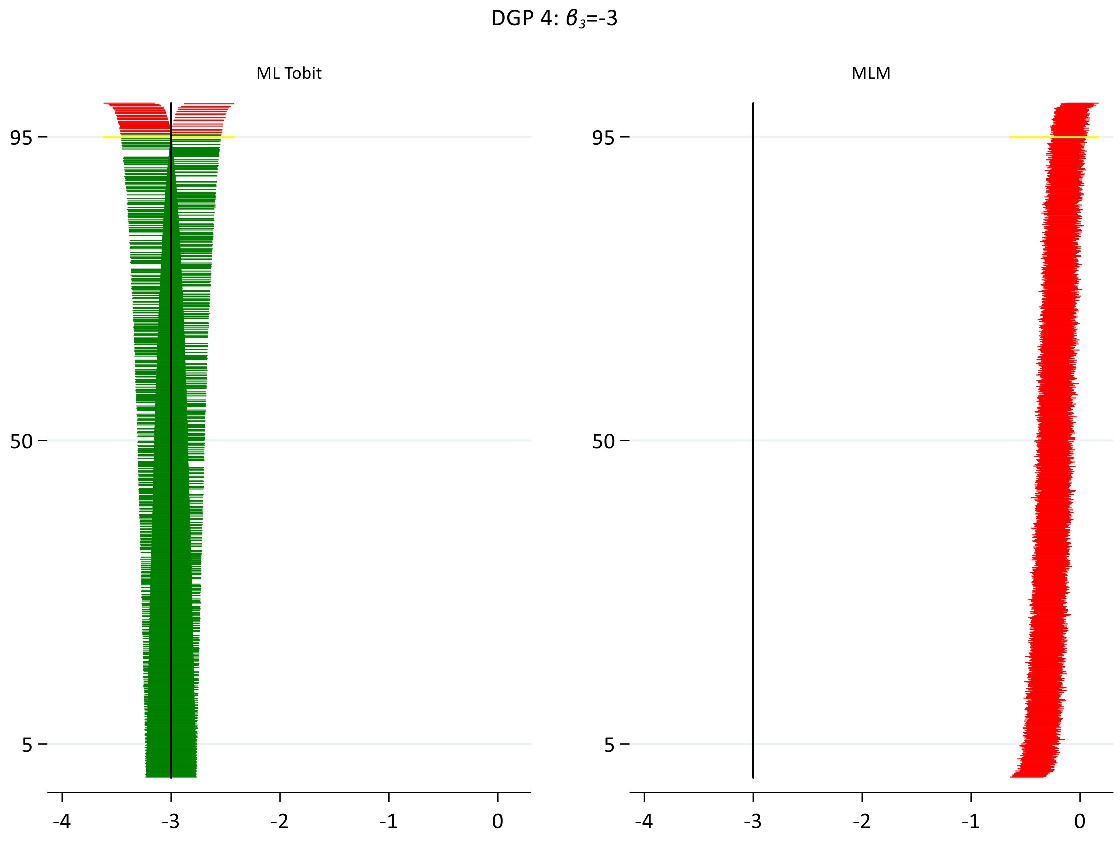

Supplementary.Figure 14: "Zip Plot" of the 1000 95% Confidence intervals for each method of analysis for DGP 4. The vertical axis is the centile of the two-sided p-value against H_0 : β_3=0 associated with the confidence interval for ML Tobit models with varying constraints of σ_ε^2

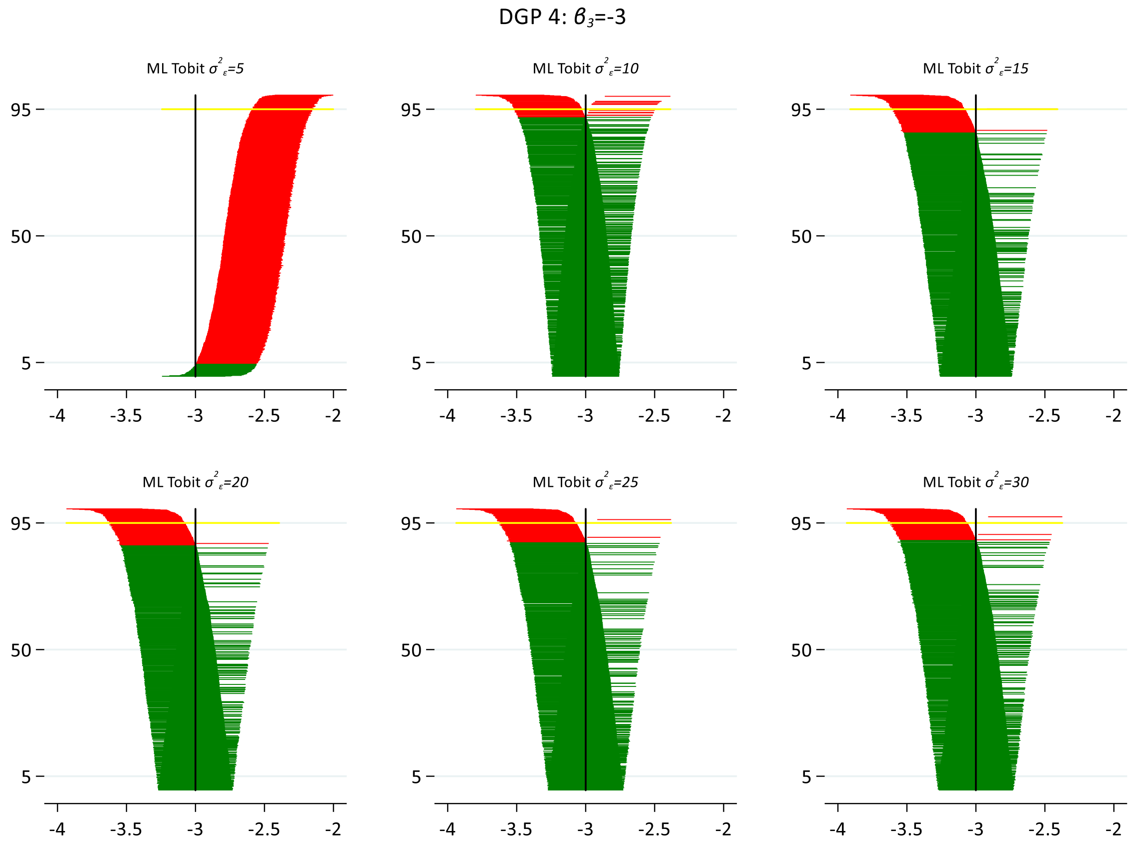

w only

Supplementary.Figure 15: "Zip Plot" of the 1000 95% Confidence intervals for each method of analysis for DGP 4. The vertical axis is the centile of the two-sided p-value against H_0 : β_3=0 associated with the confidence interval for Single-level OLS and Tobit models.

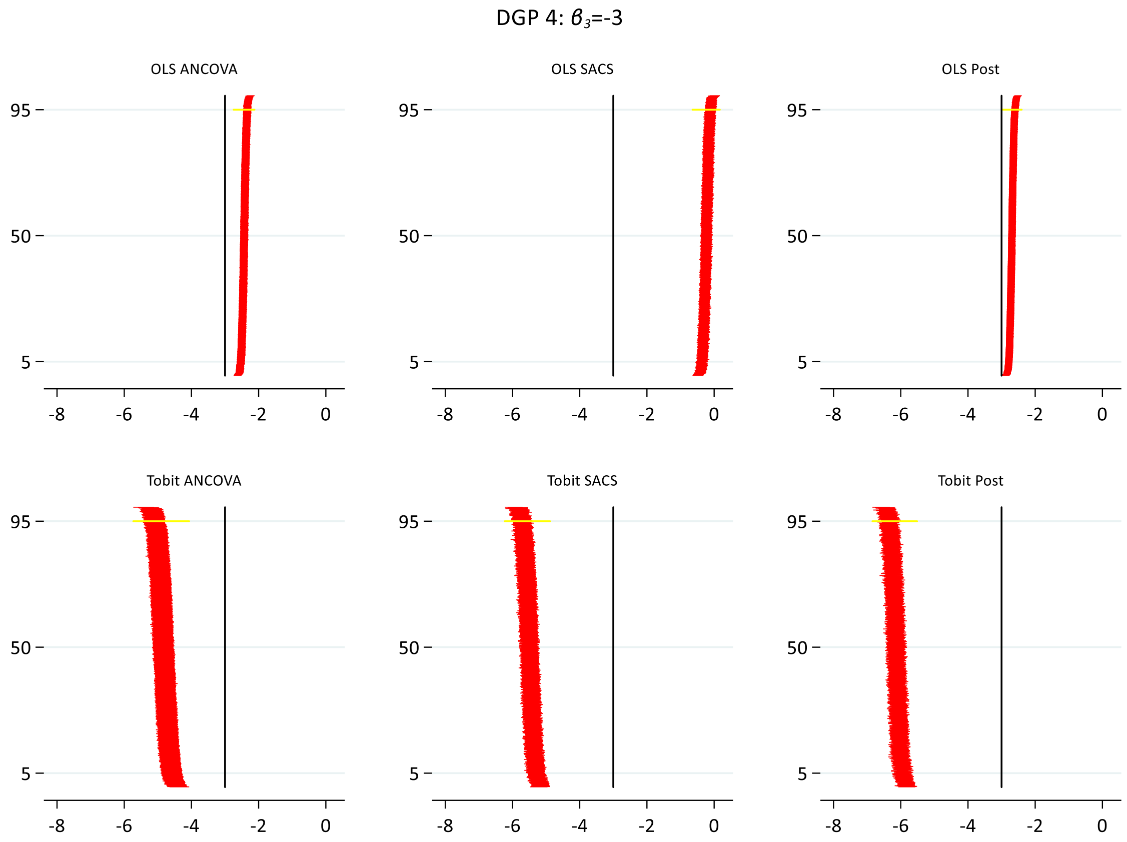

For peer review only - http://bmjopen.bmj.com/site/about/guidelines.xhtml

Supplementary.Figure 16: Marginal effect of level 1 error variance (σ_ε^2) constraint on level 2 variance components

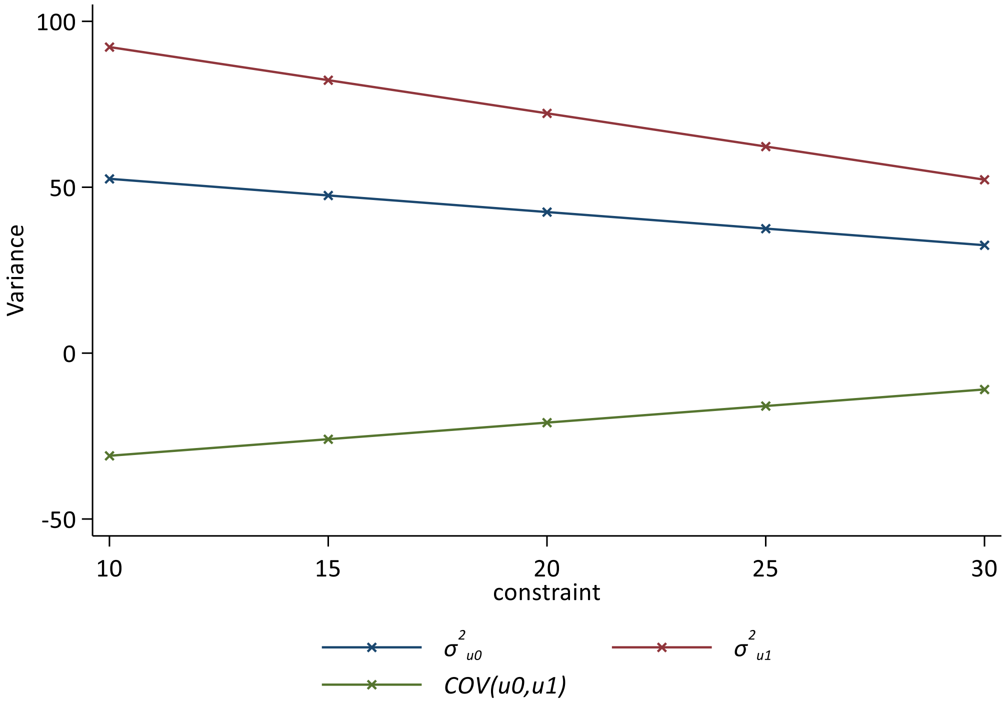

For peer review only - http://bmjopen.bmj.com/site/about/guidelines.xhtml
